# Super-resolution MRI and 2.5D deep learning for intratumoral-peritumoral radiomics in preoperative prediction of rectal cancer perineural invasion

**DOI:** 10.1186/s13244-026-02365-7

**Published:** 2026-08-22

**Authors:** Yuhang Wang, Dandan Dong, Shengming Shi, Yupeng Wu, Apekshya Singh, Jiayi Xie, Qiuyang Chen, Jianwei Zhu, Xiaofu Li

**Affiliations:** 1 Department of Magnetic Resonance Imaging Diagnostic, The Second Affiliated Hospital of Harbin Medical University, Harbin, China; 2 Medical Imaging Center, Zhuhai People’s Hospital (The Affiliated Hospital of Beijing Institute of Technology, Zhuhai Clinical Medical College of Jinan University), Zhuhai, China

**Keywords:** Rectal neoplasms, Perineural invasion, Magnetic resonance imaging, Deep learning, Radiomics

## Abstract

**Objectives:**

Preoperative prediction of perineural invasion (PNI) in rectal cancer (RC) is challenging due to limited MRI resolution and the neglect of extramural microenvironmental features. We developed a noninvasive framework integrating super-resolution MRI, 2.5D deep learning (DL), and intratumoral-peritumoral radiomics for preoperative PNI prediction.

**Materials and methods:**

A dual-center cohort of 312 RC patients with pathologically confirmed PNI status was analyzed. Preoperative MRI underwent 4× super-resolution reconstruction using a generative adversarial network (GAN) to enhance tissue definition. Radiomic features were extracted from the tumor and peritumoral regions (1–5 mm). For the 2.5D DL model, the largest tumor cross-section and adjacent axial slices served as multichannel inputs to a ResNet101 backbone using transfer learning. Slice-level features were aggregated to patient-level predictions via multi-instance learning (MIL). Feature selection employed univariate analysis, Pearson correlation, mRMR, and LASSO regression. Model performance was validated via 5-fold cross-validation and an external testing cohort.

**Results:**

The combined model, integrating MIL features, intratumoral radiomics, the optimal (2-mm) peritumoral radiomics, and clinical variables, achieved an area under the curve (AUC) of 0.912 (95% CI: 0.850–0.974) on internal validation and 0.868 (95% CI: 0.783–0.952) on external testing. Gradient-weighted class activation mapping (Grad-CAM) highlighted the tumor-neural interface, and tumor length was an independent predictor of PNI (OR = 1.062, *p* = 0.032).

**Conclusion:**

We propose a hybrid radiomic-DL framework leveraging super-resolution MRI and 2.5D spatial context to enhance preoperative prediction of PNI in RC. The model shows potential for risk stratification to support personalized neoadjuvant therapy decisions, with external testing suggesting promising generalizability.

**Key Points:**

- **Question** How can super-resolution MRI and 2.5D deep learning address the limitations of conventional imaging in predicting preoperative rectal cancer perineural invasion?
- **Findings** The integrated framework achieved an external testing AUC of 0.868, demonstrating superior performance compared to standalone radiomics and deep learning models.
- **Critical relevance statement** This study demonstrates a noninvasive framework integrating super-resolution MRI and 2.5D deep learning with intratumoral-peritumoral radiomics, offering a novel approach for preoperative rectal cancer perineural invasion prediction and potential assistance in personalized neoadjuvant therapy decisions.

**Graphical Abstract:**

**Figure:**
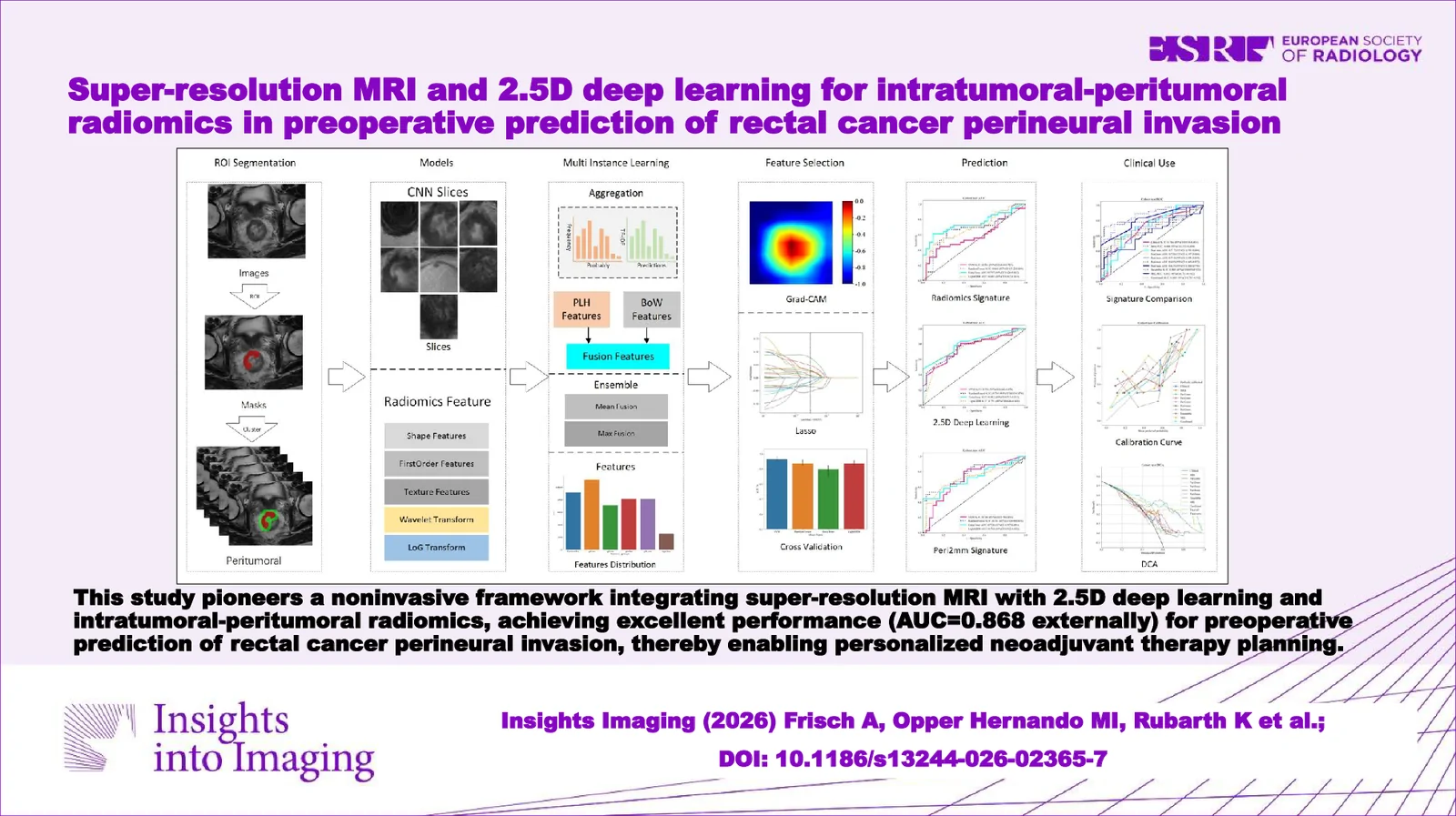

## Introduction

Rectal carcinoma (RC) remains a major global cancer burden, with rising incidence rates in recent decades [1, 2]. Despite diagnostic and therapeutic advances, many patients present with advanced disease and aggressive tumor biology that determines clinical outcomes [3]. Within prognostic determinants, perineural invasion (PNI) has gained prominence as a critical pathological marker. Defined histologically as malignant cell infiltration into neural structures—encompassing endoneurial invasion, > 33% circumferential nerve involvement, or perineurial penetration—PNI independently predicts postoperative recurrence, metastatic dissemination, and diminished survival [4, 5]. Its incorporation into AJCC TNM staging protocols reinforces its clinical significance, particularly in stratifying candidates for neoadjuvant chemoradiation (nCRT) and adjuvant chemotherapy [6, 7].

Current NCCN guidelines [8–10] maintain preoperative nCRT with total mesorectal excision (TME) as standard care for locally advanced RC, though survival outcomes remain suboptimal. Emerging European Society of Medical Oncology (ESMO) recommendations [11, 12] highlight PNI status as a crucial determinant for intensifying neoadjuvant/adjuvant regimens in high-risk Stage II patients. Paradoxically, while nCRT reduces PNI prevalence, postoperative histopathological assessment—the current diagnostic gold standard—fails to characterize pretreatment tumor biology, thereby limiting preoperative risk stratification [13–15]. Conventional MRI and biopsy lack sufficient resolution to detect subtle neural infiltration, necessitating reliance on postoperative histology and delaying personalized therapy [16, 17]. This diagnostic void underscores the urgent need for reliable noninvasive biomarkers to preoperatively assess PNI, potentially revolutionizing treatment personalization and prognostic accuracy.

Contemporary deep learning (DL) advancements have demonstrated notable superiority in medical image analysis [18]. However, conventional 2D/3D approaches present inherent limitations: 2D methods neglect multiplanar contextual information, while 3D architectures demand prohibitive computational resources and extensive annotated datasets [19]. Addressing this dichotomy, Zhang et al [20] pioneered a 2.5D medical imaging paradigm employing orthogonal lesion-centered planes as multichannel inputs. This hybrid framework preserves 3D spatial relationships while circumventing computational bottlenecks, effectively balancing interslice correlation preservation with resource efficiency [19]. Subsequent validation studies, including Takao et al [21], report 2.5D models achieving 88.7% sensitivity with statistically significant false-positive reduction (*p* < 0.001) and enhanced positive predictive values (58.9%). A growing body of research further underscores its potential in medical image segmentation [21–23]. Although radiomic approaches have been employed for PNI prediction [16, 17, 24–27], existing efforts predominantly focus on intratumoral features, neglecting the prognostic significance of extramural perineural invasion (ePNI)—a critical microenvironmental component associated with dismal survival outcomes compared to mural perineural invasion (mPNI) or PNI-negative cases (5-year DSS: 26.4% vs. 63.7% vs. 78.1%) [28].

Notably, the synergistic application of 2.5D deep learning and intratumoral-peritumoral radiomic analysis remains unexplored. This study develops a hybrid radiomic-DL model that integrates dual-region features with super-resolution imaging, investigating the clinical potential of a 2.5D DL-based framework for preoperative PNI prediction in RC.

## Materials and methods

### Study design and ethics

**Fig. 1:**
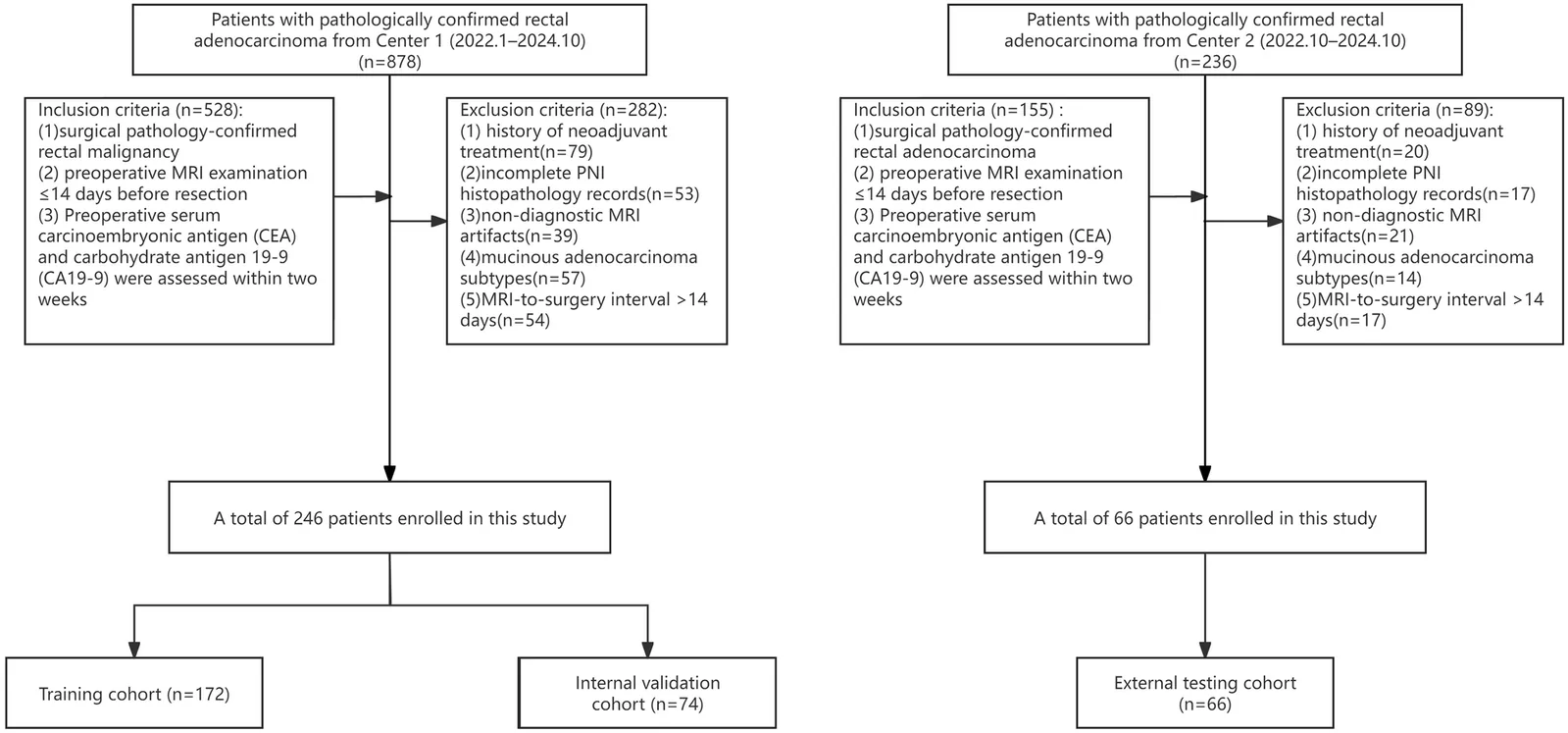
A schematic overview of the selection algorithm, detailing both inclusion thresholds and exclusionary conditions applied in the multicenter research cohort

This dual-center retrospective study was approved by the Institutional Review Boards of the Second Affiliated Hospital of Harbin Medical University (YJSKY2025-086) and Zhuhai People’s Hospital (2025-66); informed consent was waived. We enrolled 312 patients with pathologically confirmed rectal adenocarcinoma: 246 from Center 1 (training/internal validation ratio 7:3) and 66 from Center 2 (external testing). Eligibility criteria mandated: (1) surgical pathology-confirmed rectal malignancy; (2) preoperative MRI examination ≤ 14 days before resection; (3) Preoperative serum carcinoembryonic antigen (CEA) and carbohydrate antigen 19-9 (CA19-9) were assessed within 2 weeks. Exclusion parameters involved: (1) history of neoadjuvant treatment; (2) incomplete PNI histopathology records; (3) non-diagnostic MRI artifacts; (4) mucinous adenocarcinoma subtypes; (5) MRI-to-surgery interval > 14 days. Center 1 cases were stratified into training (*n* = 172) and internal validation (*n* = 74) cohorts at a 7:3 ratio. Conversely, the entire Center 2 cohort (*n* = 66) comprised the external testing cohort (Fig. 1).

Prior to surgical intervention, all enrolled participants were subjected to MRI scans, with technical specifications and imaging workflows comprehensively described in Appendix 1. Preoperative predictors encompassed demographic variables, serum biomarkers (CEA ≥ 5 ng/mL; CA19-9 ≥ 37 U/mL), and MRI-derived parameters (tumor longitudinal diameter, mrT/N staging, mrEMVI/MRF status), systematically retrieved from institutional EHR and PACS databases. The PNI status for all patients was derived from the final postoperative histopathological reports. Both participating institutions follow standardized diagnostic protocols for rectal cancer. PNI was defined according to established criteria [5, 16] as: (1) tumor cells invading any of the three layers of the nerve sheath (epineurium, perineurium, or endoneurium); or (2) tumor cells in direct contact with the nerve and wrapping around ≥ 33% of its circumference. To ensure diagnostic accuracy, S100 immunohistochemical staining was routinely utilized in clinical practice to confirm nerve structures when H&E staining was inconclusive. These clinical reports represent consensus diagnoses reviewed by senior pathologists, serving as the reliable ground truth for this study.

### Image collection and pretreatment

To optimize clarity, we implemented a generative adversarial network (GAN)-enhanced 4× super-resolution reconstruction framework via the OnekeyAI platform (https://github.com/OnekeyAI-Platform/onekey). Initial preprocessing incorporated noise reduction and intensity normalization across the MRI dataset. Subsequently, low-resolution analogs were generated through downsampling, paired with native high-resolution images for adversarial training to capture high-frequency anatomical details. The GAN structure integrated a resolution-enhancing generator and a discriminator evaluating reconstruction fidelity, iteratively refining image quality through competitive optimization (Supplementary Fig. 1). To mitigate “hallucinations” and ensure feature stability, we enforced rigorous quality control, including visual inspection by radiologists and quantitative verification via external validation.

Tumor boundaries were independently annotated by two board-certified radiologists via ITK-SNAP on the super-resolution (SR) reconstructed images, with discordant interpretations adjudicated by a third expert (> 20 years’ experience) to ensure segmentation consensus. All images underwent spatial standardization (1 mm³ isotropic voxels) using a fixed-resolution protocol, ensuring cross-modal uniformity and model robustness. Radiomic feature stability was quantified through interobserver correlation coefficient (ICC) analysis on 30 randomly selected cases. The interobserver reproducibility of the extracted radiomic features was exceptionally high, yielding a median ICC of 0.999 (range: 0.492–1.000) across all features (Supplementary Fig. 2). Specifically, the median ICCs for the DWI and T2WI sequences were 0.999 (range: 0.658–1.000) and 0.999 (range: 0.492–1.000), respectively. To ensure the robustness of the constructed model, features exhibiting ICC ≤ 0.75 were systematically excluded prior to feature selection. Visual examples demonstrating the spatial agreement of ROIs between readers are provided in Supplementary Fig. 3.

### Radiomic features

To evaluate the prognostic relevance of peritumoral microenvironments, concentric peritumoral zones were computationally generated by radially expanding tumor ROIs on GAN-enhanced T2-weighted imaging (T2WI) and diffusion-weighted imaging (DWI) sequences using a mask-based segmentation algorithm. The protocol employed 1 mm incremental expansions to create spatially resolved perilesional feature maps (Supplementary Fig. 4), with each layer independently analyzed for its predictive contribution.

Radiomic analysis encompassed both intratumoral and juxtatumoral regions, extracting morphometric (3D shape descriptors), intensity-based (histogram metrics), and high-order texture features. Texture characterization utilized computational operators including gray-level co-occurrence matrix (GLCM), gray-level run length matrix (GLRLM), gray-level size zone matrix (GLSZM), and neighborhood gray tone difference matrix (NGTDM). Feature quantification was performed using PyRadiomics v3.0.1, adhering to Image Biomarker Standardization Initiative (IBSI) guidelines. Subregional features were aggregated via pre-fusion to optimize discriminative capacity.

Feature selection involved univariate filtering (*p* < 0.05), collinearity removal (Pearson’s r > 0.9), minimum redundancy maximum relevance (mRMR) algorithm (prioritizing 32 features), and LASSO regression with 10-fold cross-validation (λ optimization) to retain statistically significant predictors (*p* < 0.05). For classification, machine learning models—Support Vector Machine (SVM) for linear tasks, Random Forest, ExtraTrees, and LightGBM for nonlinear structures—were optimized via grid search and 5-fold cross-validation. Both intratumoral (Intra) and peritumoral (PeriXmm, where “X” denotes radial distance) signatures were generated using identical pipelines.

### Development of the 2.5D deep learning framework

A 2.5D representation was generated by cropping slices adjacent to the largest tumor cross-section along the superior-inferior axis. The selected slice range included ± 1, ± 2, and ± 4 slices around the central plane to provide rich spatial context. Dual-sequence inputs comprised DWI and T2WI as separate channels, supplemented by a synthetic third channel generated through Mixup fusion: ${c}_{{mixup}}=\frac{{c}_{{DWI}}+{c}_{T2{WI}}}{2}$. These tri-channel inputs were stacked for model training, with each 2D slice assigned patient-level annotations. Transfer learning leveraged pre-trained ImageNet weights (ResNet101/VGG19/DenseNet201) to enhance feature representation efficiency under limited data constraints (Supplementary 1A).

A multi-instance learning (MIL) paradigm aggregated slice-level predictions through prediction likelihood histograms (PLH) and TF-IDF-weighted Bag-of-Words (BoW), harmonizing deep learning outputs with conventional radiomic descriptors (Supplementary 1B). To ensure strict reproducibility and prevent data leakage, all feature selection steps, including the construction of TF-IDF dictionaries and PLH bin definitions, were derived exclusively from the training dataset. Post-aggregation, the MIL signature underwent feature refinement via *t*-tests, Pearson correlation filtering, and LASSO regularization performed on the training data. Classifiers (SVM/ExtraTrees/Random Forest) were optimized using 5-fold cross-validation and grid search hyperparameter tuning. Crucially, the synthetic minority over-sampling technique (SMOTE) was applied only to the training folds within each cross-validation loop to avoid contaminating the validation data. The ExtraTrees classifier was ultimately selected for the final model construction as it yielded the highest performance on the separate internal validation set. Ensemble validation further corroborated MIL robustness (Supplementary 1C).

**Fig. 2:**
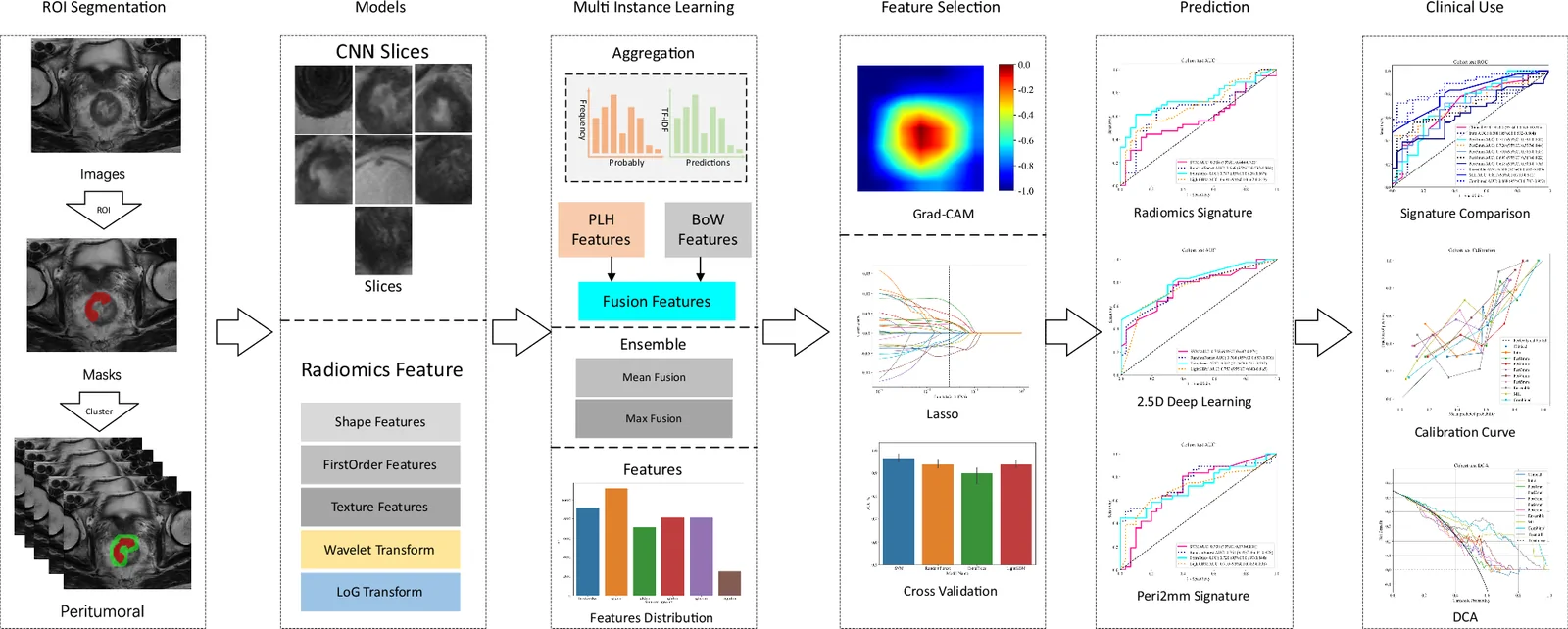
Study workflow schematic

Model evaluation integrated multivariate logistic regression (retaining features with *p* < 0.05 from univariate screening), combining clinical variables, peritumoral radiomics, and MIL outputs. Performance metrics included ROC-AUC analysis, Hosmer–Lemeshow calibration, and decision curve analysis (DCA) for clinical utility quantification. Final model selection prioritized validation cohort performance to ensure comparative fairness. The workflow schematic is shown in Fig. 2.

### Analytical methodology

Statistical analyses utilized Python 3.7.12 (OnekeyAI platform). Categorical variables were compared using chi-square tests, and continuous variables via *t*-tests or Mann–Whitney U tests. Evaluation included ROC-AUC, calibration curves, and DCA. To construct the final Combined Model and avoid the curse of dimensionality, a stepwise integration strategy was adopted. First, independent clinical predictors were identified via univariate and multivariate logistic regression analyses restricted strictly to clinical variables. Only clinical features maintaining statistical significance (*p* < 0.05) in this multivariate analysis were retained. Subsequently, the identified clinical predictor (Tumor Length), the Radiomics Signature, and the MIL Signature were treated as independent biomarkers and entered into a final multivariate logistic regression analysis to generate the nomogram.

## Results

### Cohort demographics and clinical predictors

The study analyzed 312 patients across two centers, comprising 174 (55.8%) PNI-positive and 138 (44.2%) PNI-negative cases. Demographic and clinical characteristics showed no statistically significant differences between the training, internal validation, and external testing cohorts (*p* > 0.05), ensuring balanced randomization (Table 1). In univariate analysis, tumor longitudinal diameter, MRI nodal stage, and histologic differentiation showed potential predictive value. However, in the multivariate logistic regression restricted to clinical variables, only tumor length (OR: 1.062; 95% CI: 1.014–1.112; *p* = 0.032) retained statistical significance as an independent predictor. Consequently, tumor length was the sole clinical variable integrated into the final Combined Model (Table 2).

**Table 1:** Statistical significance values denote comparative analyses between perineural invasion (PNI)-positive and PNI-negative cohorts

Feature name	Train	Val	Test	*p*-value
Age (mean ± SD, years)	64.76 ± 10.17	65.51 ± 7.96	62.32 ± 10.37	0.736
Tumor length (mean ± SD, cm)	4.19 ± 1.41	4.31 ± 1.51	4.17 ± 1.56	0.742
Gender				0.627
Female	63 (36.63)	24 (32.43)	31 (46.97)	
Male	109 (63.37)	50 (67.57)	35 (53.03)	
Tumor location				0.793
Low	67 (38.95)	28 (37.84)	32 (48.48)	
Middle	89 (51.74)	37 (50.00)	28 (42.42)	
High	16 (9.30)	9 (12.16)	6 (9.09)	
mrT_stage				0.066
2	43 (25.00)	22 (29.73)	23 (34.85)	
3	119 (69.19)	42 (56.76)	31 (46.97)	
4	10 (5.81)	10 (13.51)	12 (18.18)	
mrN_stage				0.725
0	33 (19.19)	13 (17.57)	6 (9.09)	
1	56 (32.56)	28 (37.84)	27 (40.91)	
2	83 (48.26)	33 (44.59)	33 (50.00)	
mrMRF				0.88
Negative	108 (62.79)	45 (60.81)	50 (75.76)	
Positive	64 (37.21)	29 (39.19)	16 (24.24)	
mrEMVI				0.845
Negative	110 (63.95)	49 (66.22)	41 (62.12)	
Positive	62 (36.05)	25 (33.78)	25 (37.88)	
Differentiation				0.512
Well	27 (15.70)	9 (12.16)	7 (10.61)	
Moderately	131 (76.16)	56 (75.68)	54 (81.82)	
Poorly	14 (8.14)	9 (12.16)	5 (7.58)	
CEA				0.322
Normal (< 5 ng/mL)	104 (60.47)	39 (52.70)	44 (66.67)	
Elevated (≥ 5 ng/mL)	68 (39.53)	35 (47.30)	22 (33.33)	
CA19_9				0.433
Normal (< 37 U/mL)	150 (87.21)	61 (82.43)	64 (96.97)	
Elevated (≥ 37 U/mL)	22 (12.79)	13 (17.57)	2 (3.03)	

Tumor localization relative to the border of the anus was categorized as: Low: 0–5 cm; Middle: 5.1–10 cm; High: 10.1–15 cm

*PNI* perineural invasion distance, *mrT stage* MRI T stage, *mrN_stage* MRI N stage, *mrMRF* MRI-based mesorectal fascia, *mrEMVI* MRI-based extramural vascular invasion, *CEA* carcinoembryonic antigen, *CA19-9* carbohydrate antigen 19-9

**Table 2:** Investigation of clinical features via univariate and multivariate analysis

Feature name	OR_UNI	95% CI_UNI	*p*_UNI	OR_MULTI	95% CI_MULTI	*p*_MULTI
Gender	0.873	0.7660–0.9950	0.088			
mrEMVI	0.973	0.8530–1.1110	0.733			
Age	0.998	0.9920–1.0040	0.624			
mrMRF	1.022	0.8970–1.1650	0.782			
CEA	1.045	0.9190–1.1900	0.574			
Tumor length	1.085	1.0380–1.1330	< 0.05	1.062	1.0140–1.1120	0.032
Tumor localization	1.099	0.9940–1.2140	0.122			
mrT_stage	1.115	0.9880–1.2590	0.139			
mrN_stage	1.149	1.0610–1.2460	< 0.05	1.09	1.0020–1.1860	0.093
CA19_9	1.207	1.0000–1.4580	0.1			
Differentiation	1.213	1.0660–1.3800	< 0.05	1.164	1.0240–1.3230	0.051

Tumor localization relative to the border of the anus was categorized as: Low: 0–5 cm; Middle: 5.1–10 cm; High: 10.1–15 cm

*OR_UNI* odds ratio from univariate logistic regression, *95% CI_UNI* 95% confidence interval for univariate logistic regression, *p_UNI* *p*-value from univariate logistic regression, *OR_MULTI* odds ratio from multivariate logistic regression, *95% CI_MULTI* 95% confidence interval for multivariate logistic regression, *p_MULTI* *p*-value from multivariate logistic regression, *PNI* perineural invasion distance, *mrT stage* MRI T stage, *mrN_stage* MRI N stage, *mrMRF* MRI-based mesorectal fascia, *mrEMVI* MRI-based extramural vascular invasion, *CEA* carcinoembryonic antigen, *CA19-9* carbohydrate antigen 19-9

### Deep learning architecture selection and feature visualization

**Fig. 3:**
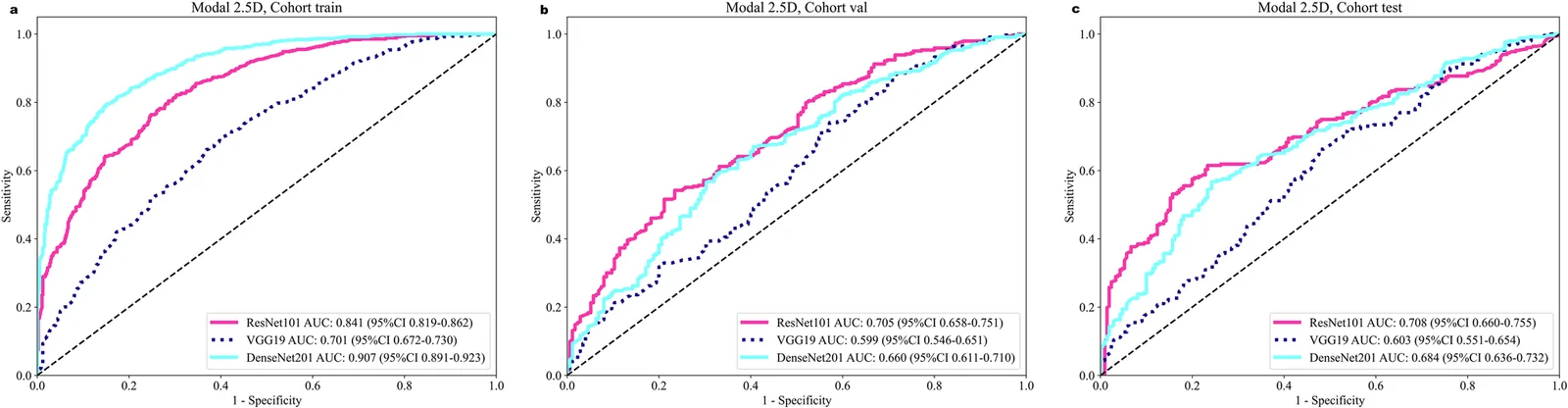
ROC curves illustrating the slice-level predictive performance of different architectures in the training (**a**), internal validation (**b**), and external testing group (**c**)

For the 2.5D deep learning component, three architectures were benchmarked (Table 3). ResNet101 demonstrated the optimal balance between learning capacity and generalization. In the internal validation cohort, it achieved an AUC of 0.705 (95% CI: 0.658–0.751), with a sensitivity of 0.539 and specificity of 0.766 (Fig. 3b). Importantly, this performance remained robust in the external testing cohort (AUC = 0.708; 95% CI: 0.660–0.755; accuracy = 0.682), confirming its stability (Fig. 3c). Conversely, DenseNet201 showed high training metrics (Fig. 3a) but poor validation performance (AUC = 0.660), suggesting overfitting. Based on these results, ResNet101 was selected as the backbone. To interpret the model’s focus, gradient-weighted class activation mapping (Grad-CAM) was employed, providing qualitative interpretability that suggested the model’s attention was focused on the tumor-neural interface (Supplementary Fig. 5).

**Table 3:** Slice-level results of different CNN models

Model name	Accuracy	AUC	95% CI	Sensitivity	Specificity	PPV	NPV	Cohort
resnet101	0.761	0.841	0.8191–0.8625	0.820	0.697	0.744	0.783	train
resnet101	0.616	0.705	0.6581–0.7512	0.539	0.766	0.819	0.459	val
resnet101	0.682	0.708	0.6605–0.7545	0.611	0.767	0.759	0.622	test
vgg19	0.648	0.701	0.6717–0.7300	0.690	0.602	0.651	0.645	train
vgg19	0.633	0.599	0.5464–0.6511	0.738	0.429	0.717	0.455	val
vgg19	0.595	0.603	0.5508–0.6543	0.655	0.524	0.623	0.558	test
densenet201	0.820	0.907	0.8906–0.9226	0.791	0.850	0.850	0.792	train
densenet201	0.618	0.660	0.6109–0.7099	0.589	0.674	0.780	0.456	val
densenet201	0.652	0.684	0.6362–0.7325	0.563	0.757	0.736	0.591	test

*AUC* area under the curve, *CI* confidence interval, *PPV* positive predictive value, *NPV* negative predictive value

### MIL fusion and radiomics signature construction

**Fig. 4:**
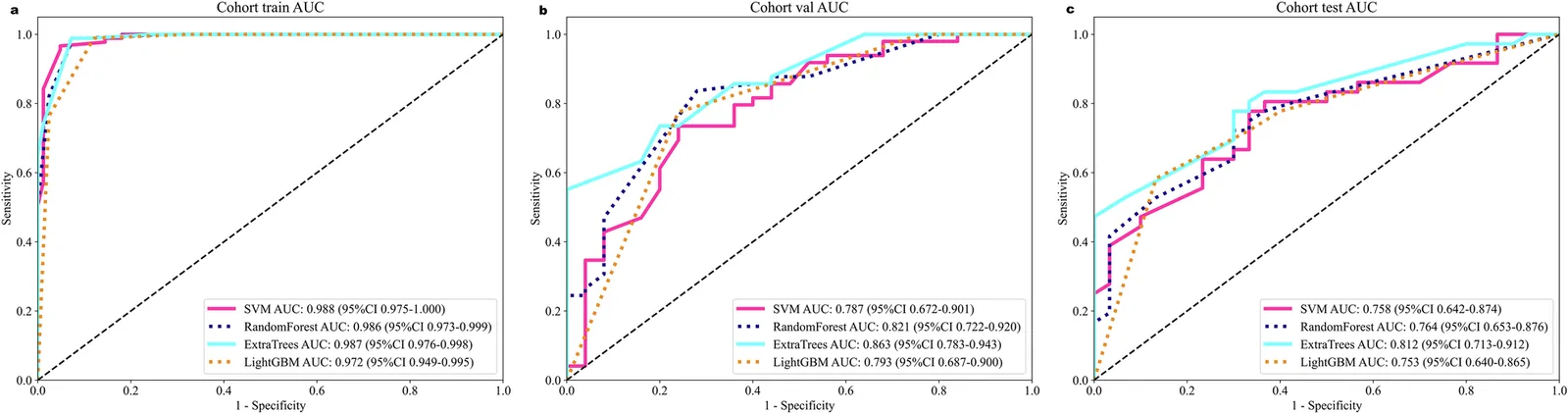
ROC curve analysis of subgroup-specific models in 2.5D multi-instance learning (MIL) features across the training (**a**), internal validation (**b**), and external testing cohort (**c**). AUC, area under the curve; CI, confidence interval

Regarding the fusion of multi-instance learning (MIL) features, the ExtraTrees classifier outperformed SVM, Random Forest, and LightGBM. In the validation phase, ExtraTrees achieved the highest AUC of 0.863 (95% CI: 0.783–0.943), with an accuracy of 0.770 and sensitivity of 0.857, significantly surpassing the Random Forest (AUC = 0.821) and SVM (AUC = 0.787) models (Fig. 4 and Table 4). Simultaneously, radiomic analysis identified the “Peri2mm” model (tumor + 2 mm margin) as the most effective signature among the tested expansions (1–5 mm). The Peri2mm model yielded an AUC of 0.798 in validation, offering a superior sensitivity-specificity balance compared to the intratumoral-only model (AUC = 0.744) (Supplementary 2A).

**Table 4:** Evaluation results of different models on the 2.5D MIL models

Model name	Accuracy	AUC	95% CI	Sensitivity	Specificity	PPV	NPV	Cohort
SVM	0.919	0.988	0.975–1.000	0.978	0.855	0.879	0.973	train
SVM	0.770	0.787	0.672–0.901	0.918	0.480	0.776	0.750	val
SVM	0.697	0.758	0.642–0.874	0.722	0.667	0.722	0.667	test
RandomForest	0.959	0.986	0.973–0.999	0.989	0.928	0.936	0.987	train
RandomForest	0.770	0.821	0.722–0.920	0.857	0.600	0.808	0.682	val
RandomForest	0.712	0.764	0.653–0.876	0.722	0.700	0.743	0.677	test
ExtraTrees	0.959	0.987	0.976–0.998	0.989	0.928	0.936	0.987	train
ExtraTrees	0.770	0.863	0.783–0.943	0.857	0.600	0.808	0.682	val
ExtraTrees	0.727	0.812	0.713–0.912	0.750	0.700	0.750	0.700	test
LightGBM	0.936	0.972	0.949–0.995	0.989	0.880	0.898	0.986	train
LightGBM	0.757	0.793	0.687–0.900	0.857	0.560	0.792	0.667	val
LightGBM	0.697	0.753	0.640–0.865	0.722	0.667	0.722	0.667	test

*AUC* area under the curve, *CI* confidence interval, *PPV* positive predictive value, *NPV* negative predictive value

### Performance of the combined model

**Fig. 5:**
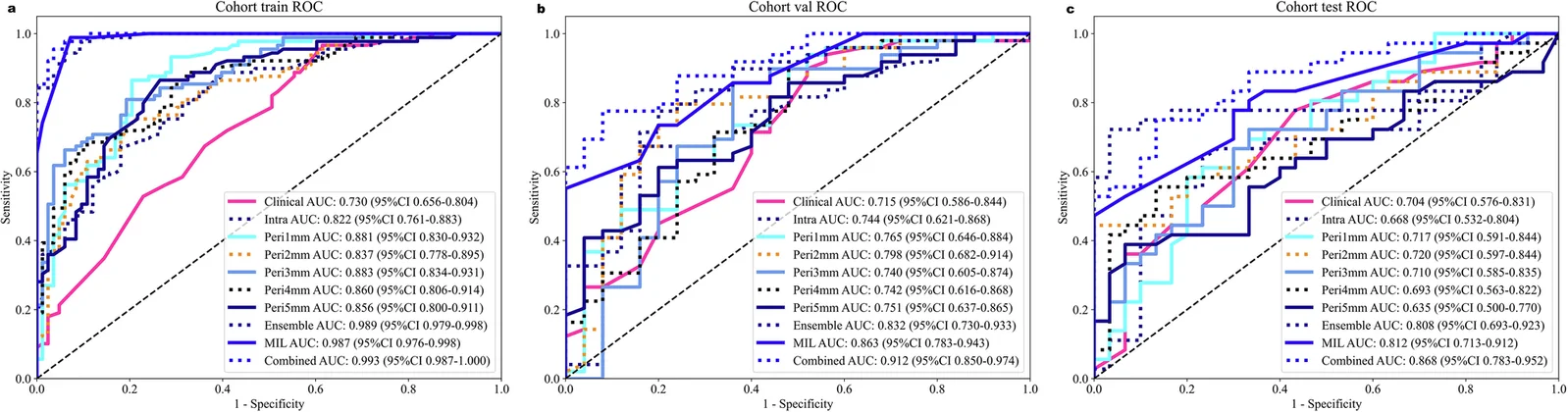
AUCs of ROC curves for different signatures across the training (**a**), internal validation (**b**), and external testing cohort (**c**)

The final Combined Model, integrating the MIL signature, intratumoral/Peri2mm radiomics, and tumor length, demonstrated exceptional discriminative ability across all datasets. In the training set, it achieved near-perfect stratification (AUC = 0.993; 95% CI: 0.987–1.000). Crucially, this advantage translated to the internal validation cohort, where the Combined Model attained an AUC of 0.912 (95% CI: 0.850–0.974), an accuracy of 0.811, and a specificity of 0.920 (Fig. 5b). In the independent external testing cohort, the model maintained high performance with an AUC of 0.868 (95% CI: 0.783–0.952), achieving higher AUC values compared to the standalone MIL (AUC = 0.812) and radiomics sub-models, although the difference between the Combined Model and the MIL model did not reach statistical significance (Fig. 5c). Detailed performance metrics are provided in Table 5.

**Table 5:** Prediction performance of peritumoral region-based rad signatures

Signature	Accuracy	AUC	95% CI	Sensitivity	Specificity	PPV	NPV	Cohort
Clinical	0.680	0.730	0.6557–0.8043	0.944	0.398	0.627	0.868	train
Intra	0.738	0.822	0.7612–0.8831	0.663	0.819	0.797	0.694	train
Peri1mm	0.826	0.881	0.8302–0.9321	0.854	0.795	0.817	0.835	train
Peri2mm	0.767	0.837	0.7778–0.8954	0.742	0.795	0.795	0.742	train
Peri3mm	0.802	0.883	0.8343–0.9310	0.798	0.807	0.816	0.788	train
Peri4mm	0.779	0.860	0.8064–0.9143	0.674	0.892	0.870	0.718	train
Peri5mm	0.785	0.856	0.7999–0.9114	0.798	0.771	0.789	0.780	train
Ensemble	0.936	0.989	0.9788–0.9984	0.933	0.940	0.943	0.929	train
MIL	0.860	0.987	0.9759–0.9981	0.742	0.988	0.985	0.781	train
Combined	0.953	0.993	0.9866–1.0000	0.944	0.964	0.966	0.941	train
Clinical	0.757	0.715	0.5860–0.8442	0.918	0.440	0.763	0.733	val
Intra	0.716	0.744	0.6209–0.8681	0.653	0.840	0.889	0.553	val
Peri1mm	0.770	0.765	0.6460–0.8838	0.918	0.480	0.776	0.750	val
Peri2mm	0.770	0.798	0.6821–0.9138	0.776	0.760	0.864	0.633	val
Peri3mm	0.770	0.740	0.6054–0.8737	0.837	0.640	0.820	0.667	val
Peri4mm	0.770	0.742	0.6162–0.8678	0.898	0.520	0.786	0.722	val
Peri5mm	0.662	0.751	0.6371–0.8650	0.592	0.800	0.853	0.500	val
Ensemble	0.770	0.832	0.7302–0.9335	0.755	0.800	0.881	0.625	val
MIL	0.689	0.863	0.7832–0.9433	0.531	1.000	1.000	0.521	val
Combined	0.811	0.912	0.8499–0.9737	0.755	0.920	0.949	0.657	val
Clinical	0.636	0.704	0.5760–0.8314	0.611	0.667	0.687	0.588	test
Intra	0.682	0.668	0.5316–0.8036	0.611	0.767	0.759	0.622	test
Peri1mm	0.667	0.717	0.5906–0.8437	0.556	0.800	0.769	0.600	test
Peri2mm	0.667	0.720	0.5972–0.8435	0.389	1.000	1.000	0.577	test
Peri3mm	0.682	0.710	0.5852–0.8352	0.694	0.667	0.714	0.645	test
Peri4mm	0.682	0.693	0.5632–0.8220	0.528	0.867	0.826	0.605	test
Peri5mm	0.606	0.635	0.4996–0.7699	0.333	0.933	0.857	0.538	test
Ensemble	0.818	0.808	0.6932–0.9235	0.694	0.967	0.962	0.725	test
MIL	0.727	0.812	0.7127–0.9123	0.750	0.700	0.750	0.700	test
Combined	0.788	0.868	0.7834–0.9518	0.722	0.867	0.867	0.722	test

PeriXmm: “ X ” denotes the peritumoral region

*AUC* area under the curve, *CI* confidence interval, *PPV* positive predictive value, *NPV* negative predictive value, *Intra* intratumoral, *MIL* multi-instance learning

### Validation and clinical utility

**Fig. 6:**
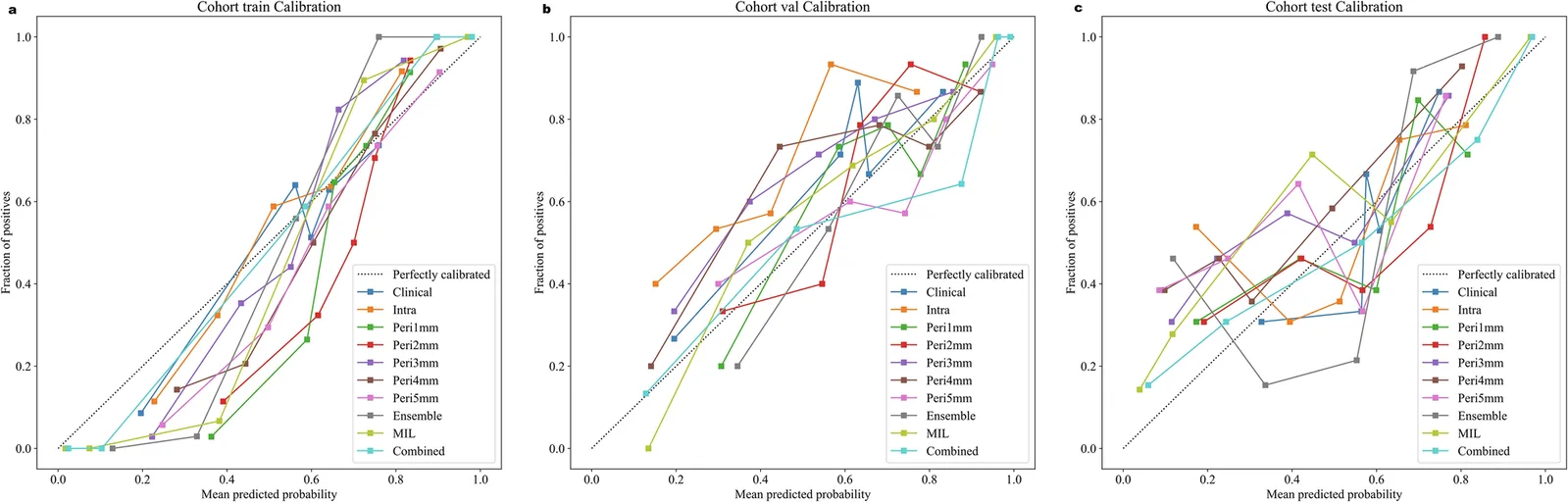
The calibration curves of all models in the training (**a**), internal validation (**b**), and external testing cohorts (**c**)

Calibration curves for the Combined Model showed high concordance between predicted probabilities and observed PNI status (Fig. 6). Decision curve analysis (DCA) demonstrated that the Combined Model provided a higher net benefit than treat-all or treat-none strategies across a wide range of threshold probabilities (Supplementary Fig. 6). DeLong tests confirmed statistically significant performance gains for the Combined Model. In the internal validation cohort, the Combined Model significantly outperformed the Clinical model (*p* = 0.005), the Intratumoral radiomics model (*p* = 0.003), and the Peri2mm radiomics model (*p* = 0.022). Similarly, in the external testing cohort, the Combined Model maintained significant superiority over the Clinical (*p* = 0.005), Intratumoral (*p* = 0.003), and Peri2mm (*p* = 0.011) models (Supplementary Fig. 7). A nomogram visually delineated the contributory weights of tumor length, intratumoral (Intra) features, peritumoral (Peri2mm) radiomics, and multi-instance learning (MIL) components in predicting pathological PNI status (Supplementary Fig. 8).

## Discussion

The noninvasive prognostic assessment of rectal cancer (RC) remains a significant clinical challenge [29, 30]. In particular, accurate preoperative identification of perineural invasion (PNI) is critical for personalized RC management, as PNI-positive patients often require intensified neoadjuvant chemoradiotherapy (nCRT) to improve survival [31–33]. We hypothesized that a framework combining super-resolution MRI, 2.5D deep learning, and peritumoral radiomics would outperform conventional methods. Our results validated this, with the combined model achieving superior discriminative performance. It is worth noting that the high AUC in the training set (0.993) is an expected outcome of the cascaded fusion architecture, where the final classifier learns from the outputs of sub-models already optimized on the training data. Importantly, the performance in the external testing cohort (AUC 0.868) suggests that the model exhibits promising generalizability and has avoided overfitting. This robustness is underpinned by our rigorous implementation of strictly isolated validation cohorts to prevent data leakage, complexity control via LASSO regularization, and data augmentation strategies (e.g., random cropping and flipping) during the deep learning training process.

This study investigated the utility of a 2.5D-GAN hybrid architecture. By utilizing orthogonal tomographic sections and super-resolution reconstruction, we mitigated the Z-axis resolution limitations of standard MRI [34]. ResNet101 was identified as the optimal backbone, balancing sensitivity and specificity. The subsequent multi-instance learning (MIL) elevated diagnostic precision, while gradient-weighted class activation mapping (Grad-CAM) elucidated region-specific saliency at tumor-neural interfaces, enhancing interpretability. Notably, tumor length was established as an independent PNI predictor. While statistically significant, the modest OR (1.062) of tumor length implies limited standalone clinical utility. However, it acts as a stable macroscopic baseline complementing microscopic imaging features; its integration into the Combined Model enhanced generalizability (AUC 0.868) over deep learning alone (AUC 0.812), thereby justifying its inclusion. These insights collectively underscore the potential of hybrid radiomic-deep learning paradigms.

Our methodology introduces three principal innovations. First, we addressed MRI resolution anisotropy via a 2.5D-GAN architecture. Regarding the super-resolution reconstruction, a primary concern in GAN-based methods is the potential introduction of artificial textures or “hallucinations.” However, our study provides robust evidence of feature stability: the extracted radiomic signature achieved high predictive accuracy in the independent external testing cohort (AUC = 0.868). This confirms that the super-resolution process enhanced genuine, generalizable tumor heterogeneity rather than generating random noise, thereby ensuring the reliability of the downstream analysis. Second, our 2.5D approach synergizes 2D efficiency with 3D fidelity. Zhang et al [19] demonstrated that 2.5D architectures require fewer parameters than 3D CNNs while preserving spatial context. Similarly, Chakrabarty et al [35] successfully employed 2.5D models for glioma genotyping. By aggregating orthogonal planes, our framework captures interslice correlations [36] without the prohibitive computational costs of 3D models [37]. A notable challenge in applying 2.5D deep learning to PNI prediction is the potential for label noise introduced by slice-level labeling. Because ground-truth PNI status is determined at the patient level via pathology, assigning this global label to every MRI slice implies that all cross-sections of a PNI-positive tumor exhibit predictive features. In reality, PNI is heterogeneous; specific slices may not visually depict neural invasion, creating a “weakly supervised” learning scenario. We addressed this by implementing a Multi-Instance Learning (MIL) strategy combined with Ensemble Fusion. By treating the patient as a “bag” of slices and aggregating features via prediction likelihood histograms (PLH) and Bag-of-Words (BoW) alongside ensemble pooling, the model focuses on the distributional patterns of malignancy across the entire tumor volume. This aggregation effectively filters out noise from non-informative slices, enabling robust patient-level predictions. Third, unlike prior studies focusing solely on intratumoral features [16, 17, 24–27], we integrated the extramural microenvironment. Since neural structures often localize beyond the muscularis mucosae, extramural PNI correlates with dismal survival [28]. We systematically analyzed the intratumoral and five concentric peritumoral zones. The 2 mm peritumoral model (Peri2mm) achieved optimal performance. Anatomically, this selection reflects a balance between sensitivity and specificity. A 2 mm margin captures the immediate “invasive front” and the tumor-host interface relevant to perineural invasion, whereas a narrower margin (1 mm) may miss subtle microenvironmental changes. Conversely, given the limited thickness of the mesorectum, wider margins (3–5 mm) risk including non-specific tissues—such as the mesorectal fascia or healthy adipose tissue—thereby introducing background noise that dilutes tumor-specific signals. Technically, the application of super-resolution GAN was pivotal for this analysis; by minimizing partial volume effects and sharpening tumor boundaries, the SR reconstruction ensured that this precise 2 mm zone could be delineated with high geometric fidelity, despite the potential concerns regarding generative artifacts. Comparatively, while Li et al [38] developed a CT-based nomogram, our MRI-based framework offers superior soft-tissue contrast without ionizing radiation. Furthermore, our 2.5D deep learning architecture overcomes the inherent limitations of traditional radiomics, such as poor interpretability and manual feature dependency [39]. Finally, multicenter validation across heterogeneous scanners enhanced model robustness, a critical advance over single-center studies.

Several limitations warrant consideration. First, this is a retrospective two-center study. While the multicenter cohort (*N* = 312) is relatively large, the external testing cohort (*n* = 66) is relatively small, which may limit the ability to definitively claim broad clinical generalizability. Second, the current protocol relies on manual ROI delineation, which may introduce interobserver variability and increase the workload in clinical practice. We aim to address this by integrating automated segmentation algorithms in future work. Third, while the model shows promising predictive performance, this study lacks a prospective clinical impact assessment. Therefore, the ability of the model to directly influence neoadjuvant therapy decisions and improve patient outcomes remains to be validated. Large-scale prospective clinical trials are essential to confirm its clinical utility and facilitate its translation into routine practice.

In conclusion, we established a noninvasive framework harmonizing 2.5D DL, super-resolution MRI, and dual-region radiomics for PNI prediction. The combined model demonstrates promising calibration and predictive generalizability, outperforming existing methods. Clinically, this tool may assist in the identification of high-risk patients, potentially supporting personalized neoadjuvant therapy decisions. However, further large-scale prospective validation is required to confirm its clinical impact.

## Supplementary information

ELECTRONIC SUPPLEMENTARY MATERIAL

13244_2026_2365_MOESM2_ESM

## Data Availability

The datasets generated and analyzed during the current study are not publicly available due to the PACS system regulations by the Second Affiliated Hospital of Harbin Medical University and Zhuhai People’s Hospital but are available from the corresponding author upon reasonable request.

## Abbreviations

AJCC: American Joint Committee on Cancer
AUC: Area under the receiver operating characteristic curve
BoW: Bag-of-Words
CA19-9: Carbohydrate antigen 19-9
CEA: Carcinoembryonic antigen
CI: Confidence interval
DCA: Decision curve analysis
DL: Deep learning
DWI: Diffusion-weighted imaging
ePNI: Extramural perineural invasion
ESMO: European Society of Medical Oncology
GAN: Generative adversarial network
Grad-CAM: Gradient-weighted class activation mapping
ICC: Interobserver correlation coefficient
LASSO: Least absolute shrinkage and selection operator
MIL: Multi-instance learning
mPNI: Mural perineural invasion
mrEMVI: MRI-based extramural venous invasion
mRMR: minimum redundancy maximum relevance
mrMRF: MRI-based mesorectal fascia
mrN stage: MRI N stage
mrT stage: MRI T stage
NCCN: National Comprehensive Cancer Network
nCRT: Neoadjuvant chemoradiation
NGTDM: Neighborhood gray tone difference matrix
NPV: Negative predictive value
OR: Odds ratio
PLH: Prediction likelihood histograms
PNI: Perineural invasion
PPV: Positive predictive value
RC: Rectal cancer
ROC: Receiver operating characteristic
ROI: Region of interest
SR: Super-resolution
SVM: Support vector machine
T2WI: T2-weighted imaging
TF-IDF: Term frequency-inverse document frequency
TME: Total mesorectal excision
