# Supplementary material for: Super-resolution MRI and 2.5D deep learning for intratumoral-peritumoral radiomics in preoperative prediction of rectal cancer perineural invasion
Source: Insights Imaging. 2026 Aug 22;17:205. doi: 10.1186/s13244-026-02365-7 (PMC13499786; doi:10.1186/s13244-026-02365-7)
Supplement: Supplementary file 1 [file 13244_2026_2365_MOESM1_ESM.pdf]

Super-Resolution MRI and 2.5D Deep Learning for Intratumoral-Peritumoral Radiomics in Preoperative Prediction of Rectal Cancer Perineural Invasion

ELECTRONIC SUPPLEMENTARY MATERIAL

Appendix 1

This study utilized two GE Discovery MR750w 3.0T MRI scanners from Center 1 (the Second Affiliated Hospital of Harbin Medical University) and Center 2 (Zhuhai People's Hospital) to perform standardized magnetic resonance imaging (MRI) examinations, acquiring multiplanar images including oblique axial, sagittal, and coronal views. The analysis focused on high-resolution oblique axial T2-weighted imaging (T2WI) and diffusion-weighted imaging (DWI) sequences. Prior to the MRI scans, a water enema was administered to minimize artifacts caused by intestinal contents.For the scanner at Center 1, the oblique axial T2WI parameters were as follows: repetition time (TR)/echo time (TE) = 5,990 ms/125.7 ms, field of view (FOV) = 20 cm, slice thickness = 3.6 mm, and slice spacing = 0.3 mm. The DWI parameters were: TR/TE = 4,881 ms/70.2 ms, FOV = 28 cm, slice thickness = 3.6 mm, and slice spacing = 0.3 mm.For the scanner at Center 2, the oblique axial T2WI parameters were: TR/TE = 5,500 ms/120 ms, FOV = 20 cm, slice thickness = 3.6 mm, and slice spacing = 0.3 mm. The DWI parameters were: TR/TE = 5,000 ms/80 ms, FOV = 30 cm, slice thickness = 3.6 mm, and slice spacing = 0.3 mm.

Supplementary 1A. Details of Deep Learning training Process

Data Augmentation: In this study, we applied Z-score normalization to standardize the intensity distribution across RGB channels in images, which were then used as input for our model. During the training phase, we employed real-time data augmentation techniques, such as random cropping and horizontal and vertical flipping. For the test images, we restricted the preprocessing to normalization only, without applying any augmentation.

Data Normalization: We normalized the grayscale values of the image slices using a min-max transformation to adjust the range to [-1, 1]. Each cropped subregion image was resized to 224 × 224 pixels using nearest neighbor interpolation, ensuring compatibility with the input requirements of our deep learning models.

training Parameters: To optimize the learning process for our specific image dataset, we adjusted the learning rate using a cosine decay strategy, detailed in the following equation:

$$\eta_t = \eta_{min}^i + \frac{1}{2}\left(\eta_{max}^i - \eta_{min}^i\right)\left(1 + \cos\left(\frac{T_{cur}}{T_i}\pi\right)\right)$$

where η_{min}^i is set to 0, and η_{max}^i to 0.01, with T_i representing the number of iteration epochs. We utilized SGD (Stochastic Gradient Descent) as the optimizer, and softmax cross entropy as the loss function.

Supplementary 1B. Details Multi-Instance Learning-Based Feature Fusion

In our study, we implemented two multi-instance learning fusion techniques. Using 2.5D deep learning models, we created Predict Likelihood Histograms (PLH) that map out the predictive probabilities and labels for each slice, offering a probabilistic summary of the prediction landscape. We also applied a Bag of Words (BoW) approach, segmenting each image into slices and extracting

data to compile seven predictive results per sample, using the Term Frequency-Inverse Document Frequency (TF-IDF) method for analysis. Additionally, we enhanced our model by integrating PLH and BoW features with radiomic data, leveraging diverse data sources to improve the representational power and accuracy of our classification tasks.

Our multi-instance learning approach aimed to enhance predictive accuracy by integrating various data points from a single sample into a comprehensive feature set, involving:

1. **Slice Prediction**: Each slice was analyzed using the deep learning model to derive probabilities and labels, denoted as $Slice_{prob}$ and $Slice_{pred}$, retained to two decimal places.

2. **Multi Instance Learning Feature Aggregation**:

   – **Histogram Feature Aggregation**:

     - Distinct numbers were treated as "bins" to count occurrences across types. Crucially, the definition of these bins (e.g., value ranges) was determined solely based on the distribution of the training set to ensure that the feature construction process remained independent of the validation/test data.

     - Frequencies of $Slice_{prob}$ and $Slice_{pred}$ in each bin were tallied and normalized using min-max normalization, resulting in $Histo_{prob}$ and $Histo_{pred}$.

   – **Bag of Words (BoW) Feature Aggregation**:

     - A dictionary was constructed from unique elements in $\mathrm{Slice}_{\mathrm{prob}}$ and $\mathrm{Slice}_{\mathrm{pred}}$. To ensure strict reproducibility and prevent data leakage, this vocabulary dictionary was built using only the training cohort. Any new elements appearing in the validation or test sets were ignored.

- Each slice was represented as a vector noting the frequency of each dictionary element, with a TF-IDF transformation applied to emphasize informative features. The Inverse Document Frequency (IDF) weights were also calculated exclusively from the training corpus.
- This resulted in a BoW feature representation for each slice, encapsulating both the presence and significance of features.

3. **Feature Early Fusion**: We integrated $Histo_{prob}$, $Histo_{pred}$, Bow_{prob}, and Bow_{pred} using a feature concatenation method ($\oplus$), combining these into a single comprehensive feature vector:

$$feature_{fusion} = Histo_{prob} \oplus Histo_{pred} \oplus Bow_{prob} \oplus Bow_{pred}$$

Supplementary 1C. Details Different Ensemble Fusion Method

Leveraging the concept of ensemble methods, we combined the predicted probabilities from different cross-sectional images of the same patient to enhance predictive performance and robustness. Specifically, we implemented two distinct approaches to perform this ensemble fusion: using the maximum and the average values of the predictions.

The first approach involves taking the maximum predicted probability among all slices for a given patient. This method tends to highlight the most abnormal features detected across slices, which might indicate the presence of pathological findings more assertively. The mathematical representation for the maximum ensemble method is as follows:

$$P_{\text{max}} = \max\left(P_{+1}, P_{+2}, P_{+4}, P_{max\_roi}, P_{-1}, P_{-2}, P_{-4}\right) P_{\text{max}} = \max\left(P_{+1}, P_{+2}, P_{+4}, P_{max\_roi}, P_{-1}, P_{-2}, P_{-4}\right)$$

The second approach averages the predicted probabilities across all slices. This method provides a more balanced view that incorporates contributions from all slices, potentially smoothing out any

anomalies that might appear in a single slice. The formula for calculating the average predicted probability is:

$$P_{\text{mean}} = \frac{1}{n}\sum\left(P_{+1}, P_{+2}, P_{+4}, P_{max\_roi}, P_{-1}, P_{-2}, P_{-4}\right)$$

where P_{max} is the maximum probability , whereP_{mean} represents the average probability and$P_{+1}, P_{+2}, P_{+4}, P_{max_roi}, P_{-1}, P_{-2}, P_{-4}$are the predicted probabilities for each slice.

Detailed comparisons between these two ensemble methods are available, offering insights into their respective advantages and limitations in the context of medical image analysis. These comparative analyses help in selecting the most appropriate ensemble strategy based on specific clinical and diagnostic requirements.

Results of Ensemble Fusion

Method	Acc	AUC	95% CI	Sensitivity	Specificity	PPV	NPV	Cohort
mean	0.930	0.989	0.9788-0.9984	0.978	0.880	0.897	0.973	train
mean	0.797	0.832	0.7302-0.9335	0.898	0.600	0.815	0.750	val
mean	0.758	0.808	0.6932-0.9235	0.778	0.733	0.778	0.733	test
max	0.587	0.938	0.9045-0.9706	1.000	0.145	0.556	1.000	train
max	0.676	0.804	0.6989-0.9092	1.000	0.040	0.671	1.000	val
max	0.591	0.812	0.7017-0.9224	0.917	0.200	0.579	0.667	test

Table S1. Metrics of different ensemble fusion methods

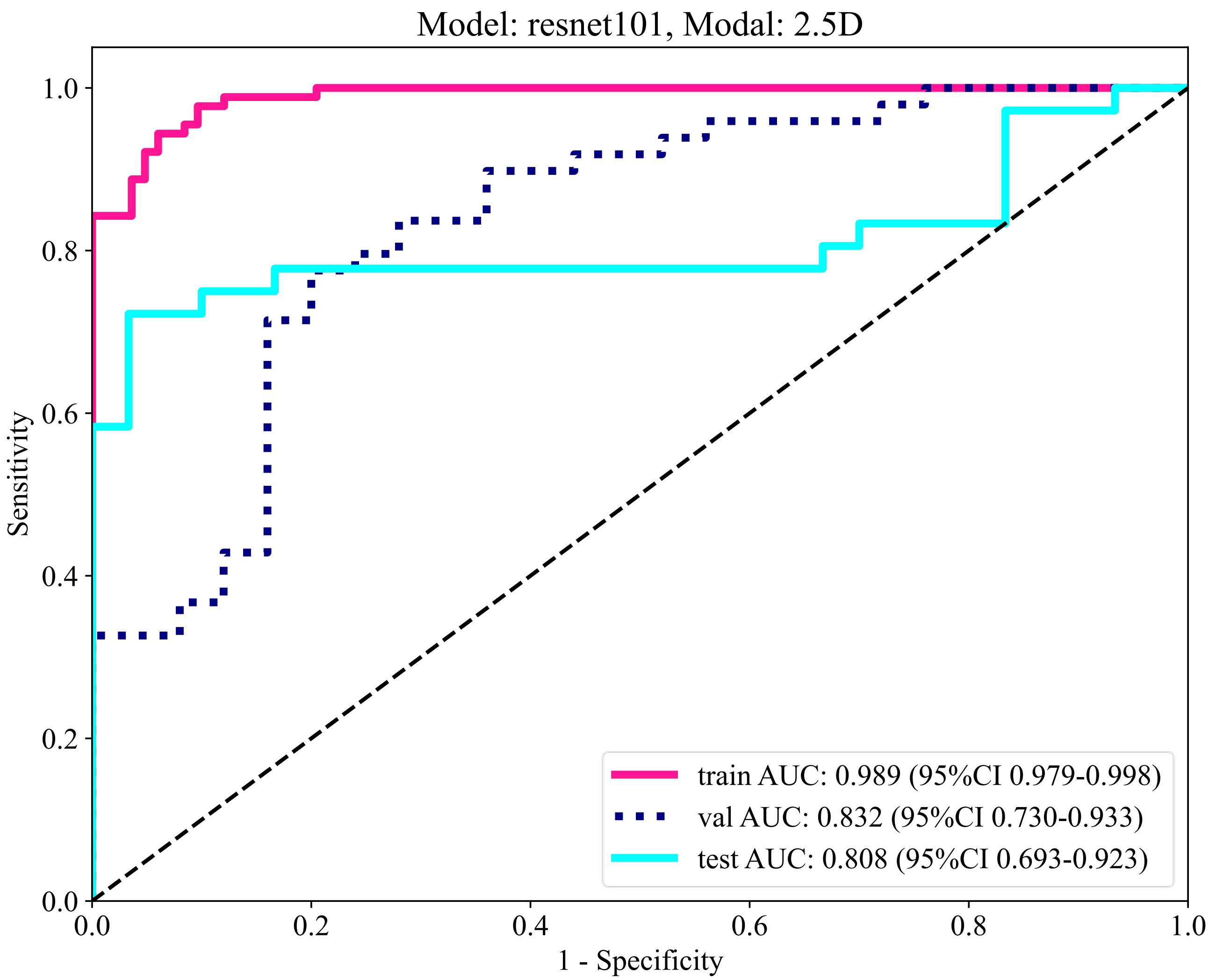
Model: resnet101, Modal: 2.5D
Sensitivity
1 - Specificity
0.0
0.2
0.4
0.6
0.8
1.0
train AUC: 0.989 (95%CI 0.979-0.998)
val AUC: 0.832 (95%CI 0.730-0.933)
test AUC: 0.808 (95%CI 0.693-0.923)

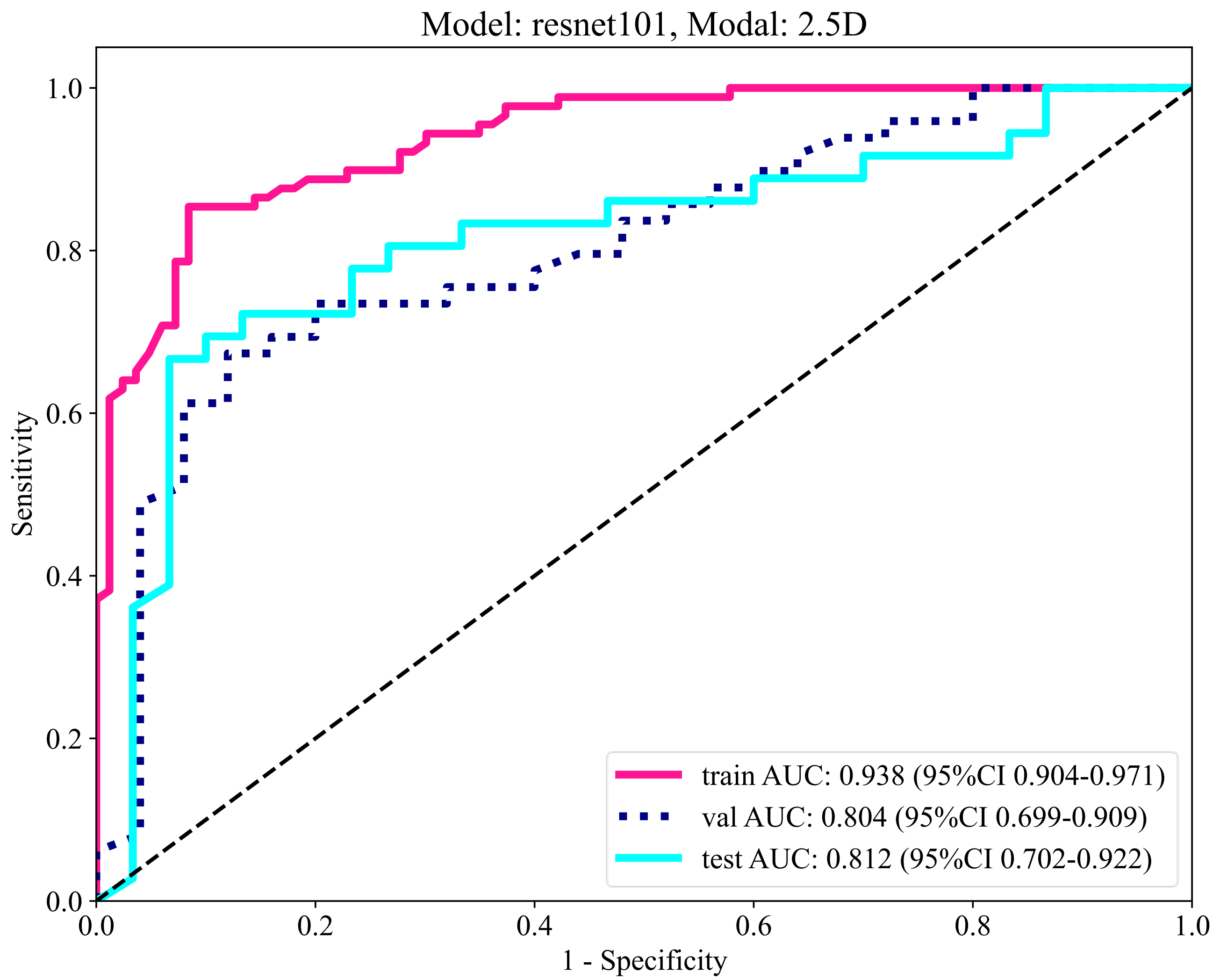

Figure S9. ROC curves of different ensemble fusion methods.

Supplementary 2A. Details of Peritumoral Signature

Peri1mm

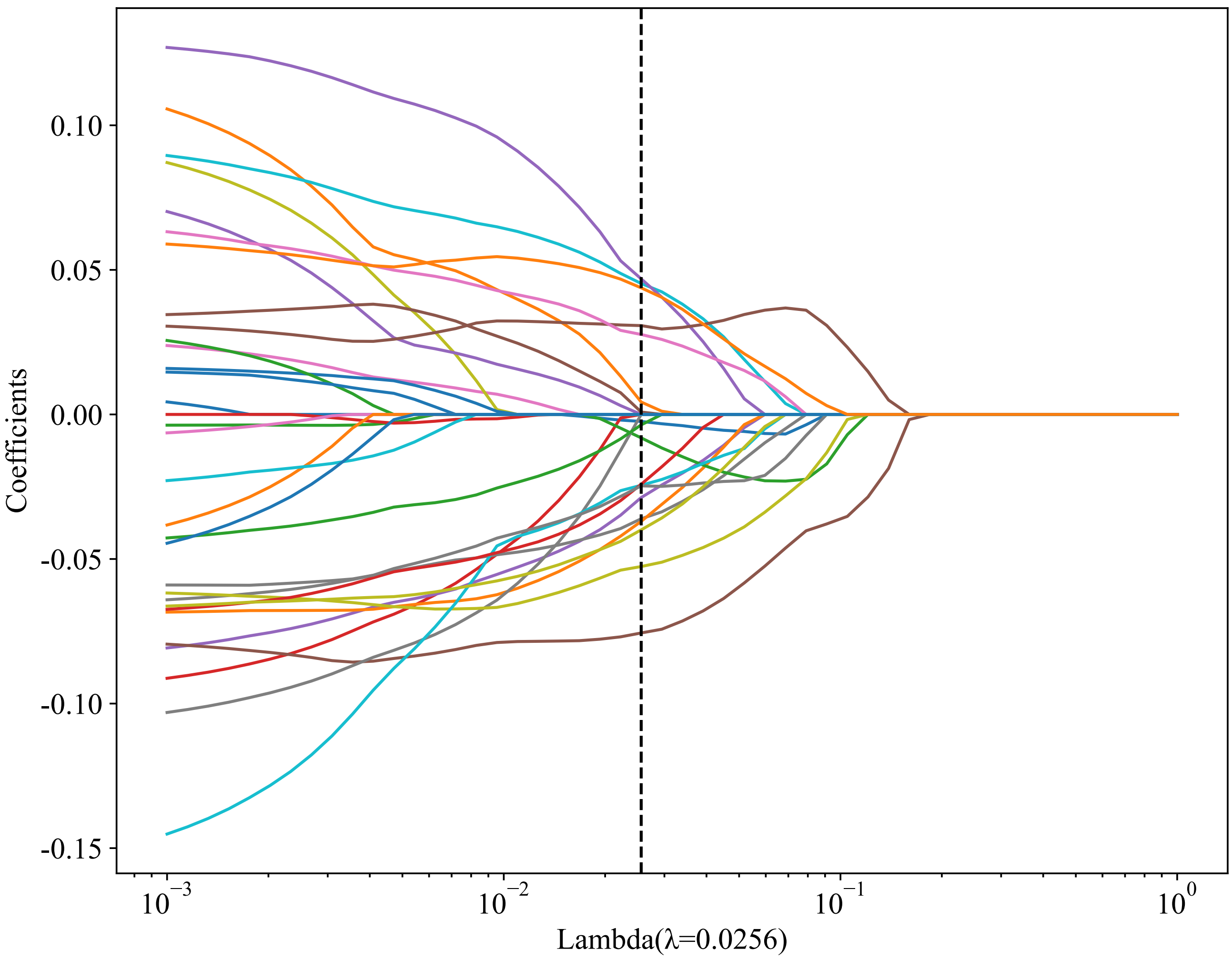

a

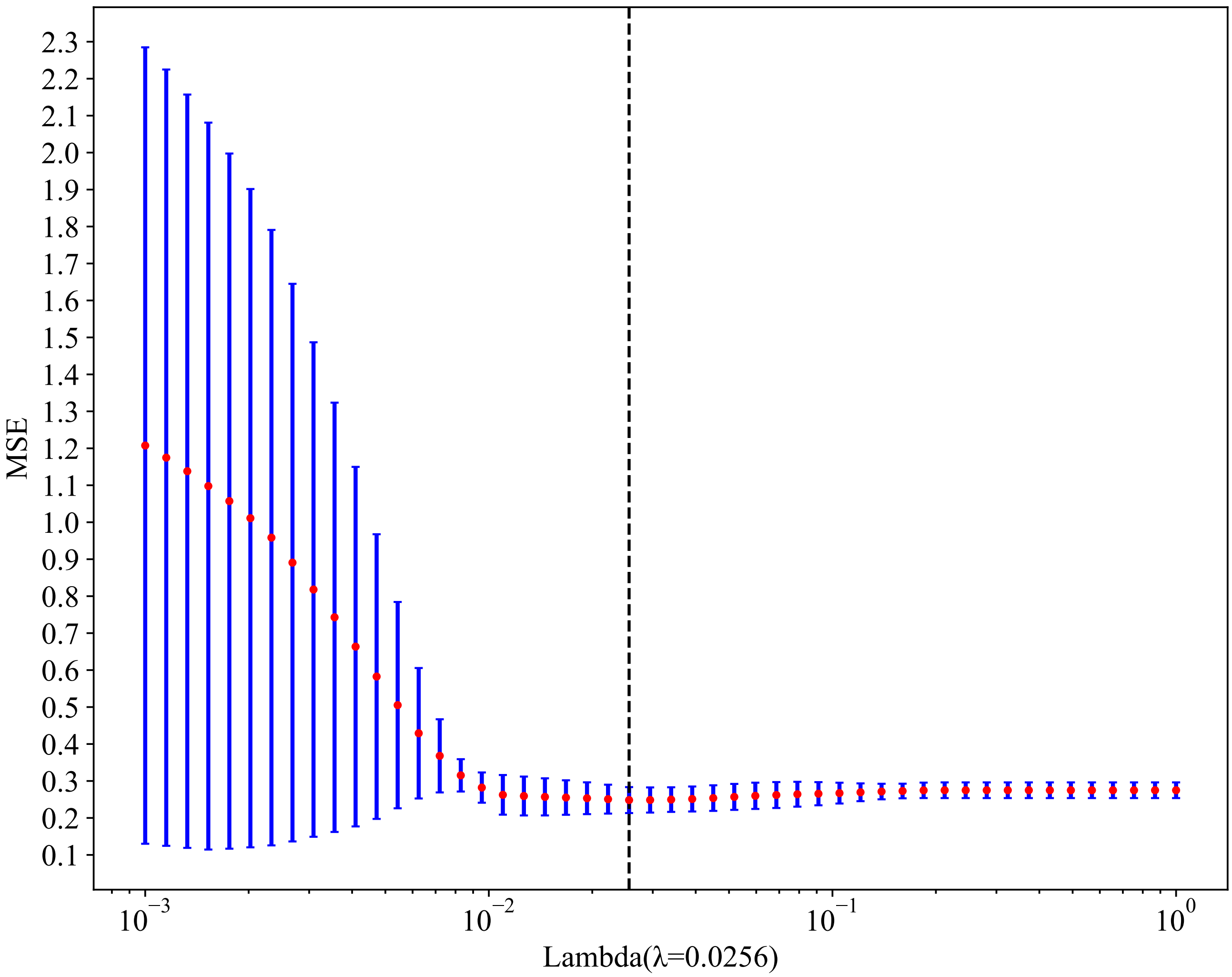
MSE
Lambda(λ=0.0256)

b

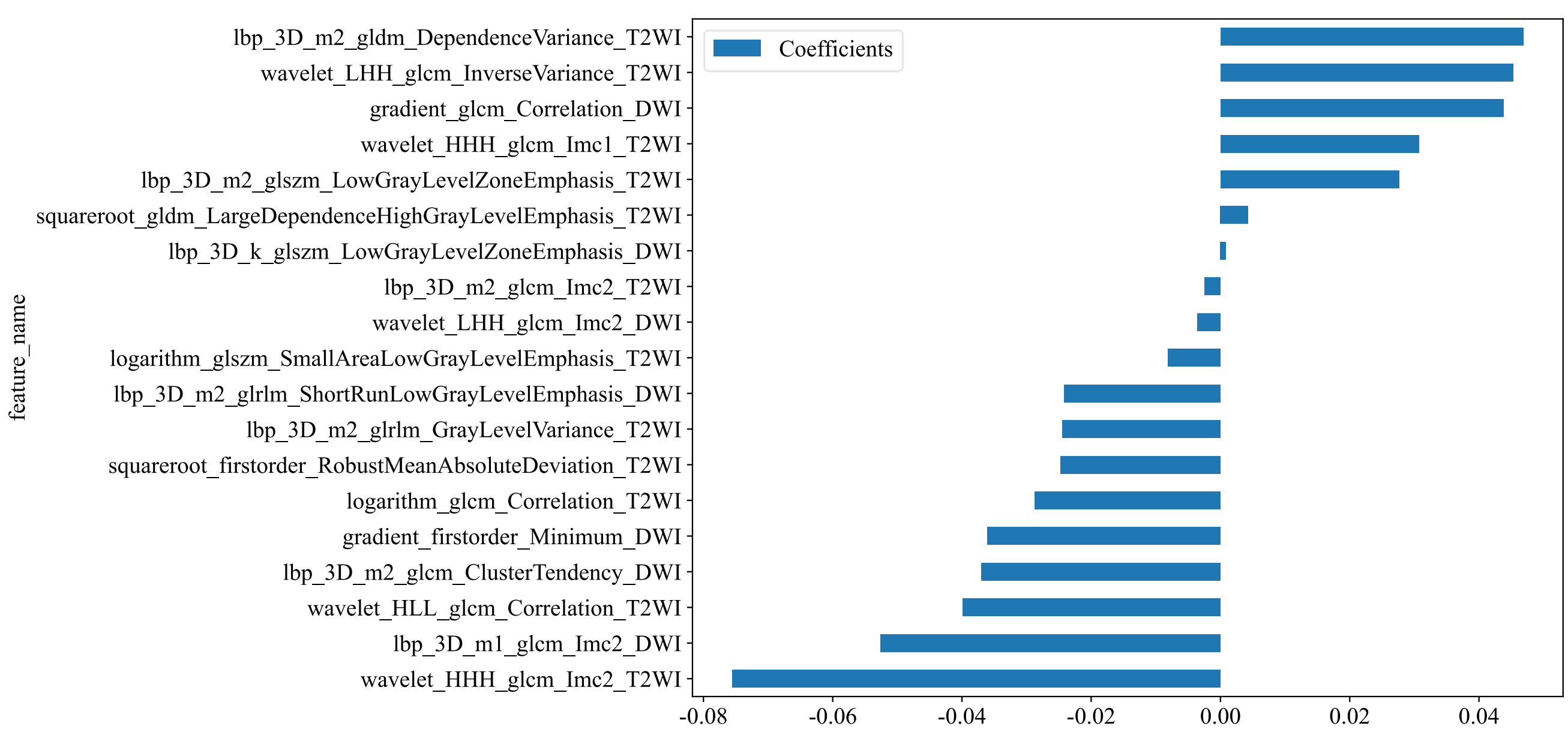
Coefficients
feature_name
lbp_3D_m2_gldm_DependenceVariance_T2WI
wavelet_LHH_glcm_InverseVariance_T2WI
gradient_glcm_Correlation_DWI
wavelet_HHH_glcm_Imc1_T2WI
lbp_3D_m2_glszm_LowGrayLevelZoneEmphasis_T2WI
squareroot_gldm_LargeDependenceHighGrayLevelEmphasis_T2WI
lbp_3D_k_glszm_LowGrayLevelZoneEmphasis_DWI
lbp_3D_m2_glcm_Imc2_T2WI
wavelet_LHH_glcm_Imc2_DWI
logarithm_glszm_SmallAreaLowGrayLevelEmphasis_T2WI
lbp_3D_m2_glrlm_ShortRunLowGrayLevelEmphasis_DWI
lbp_3D_m2_glrlm_GrayLevelVariance_T2WI
squareroot_firstorder_RobustMeanAbsoluteDeviation_T2WI
logarithm_glcm_Correlation_T2WI
gradient_firstorder_Minimum_DWI
lbp_3D_m2_glcm_ClusterTendency_DWI
wavelet_HLL_glcm_Correlation_T2WI
lbp_3D_m1_glcm_Imc2_DWI
wavelet_HHH_glcm_Imc2_T2WI

c

Figure S10. Coefficients of 10-fold cross-validation (a), MSE of 10-fold cross-validation (b), the histogram of the Rad-score based on the selected features (c) for Peri1mm.

model_name	Accuracy	AUC	95% CI	Sensitivity	Specificity	PPV	NPV	Cohort
SVM	0.686	0.766	0.696 - 0.836	0.528	0.855	0.797	0.628	train
SVM	0.730	0.651	0.511 - 0.791	0.857	0.480	0.764	0.632	val
SVM	0.621	0.610	0.471 - 0.749	0.472	0.800	0.739	0.558	test
RandomForest	0.826	0.885	0.837 - 0.934	0.854	0.795	0.817	0.835	train
RandomForest	0.730	0.708	0.583 - 0.834	0.878	0.440	0.754	0.647	val
RandomForest	0.652	0.700	0.574 - 0.826	0.722	0.567	0.667	0.630	test
ExtraTrees	0.826	0.881	0.830 - 0.932	0.854	0.795	0.817	0.835	train
ExtraTrees	0.770	0.765	0.646 - 0.884	0.918	0.480	0.776	0.750	val
ExtraTrees	0.667	0.717	0.591 - 0.844	0.556	0.800	0.769	0.600	test
LightGBM	0.901	0.972	0.953 - 0.990	0.876	0.928	0.929	0.875	train
LightGBM	0.716	0.718	0.586 - 0.850	0.755	0.640	0.804	0.571	val
LightGBM	0.561	0.625	0.489 - 0.760	0.250	0.933	0.818	0.509	test

Table S2. Model performance of different machine learning algorithms (Peri1mm).

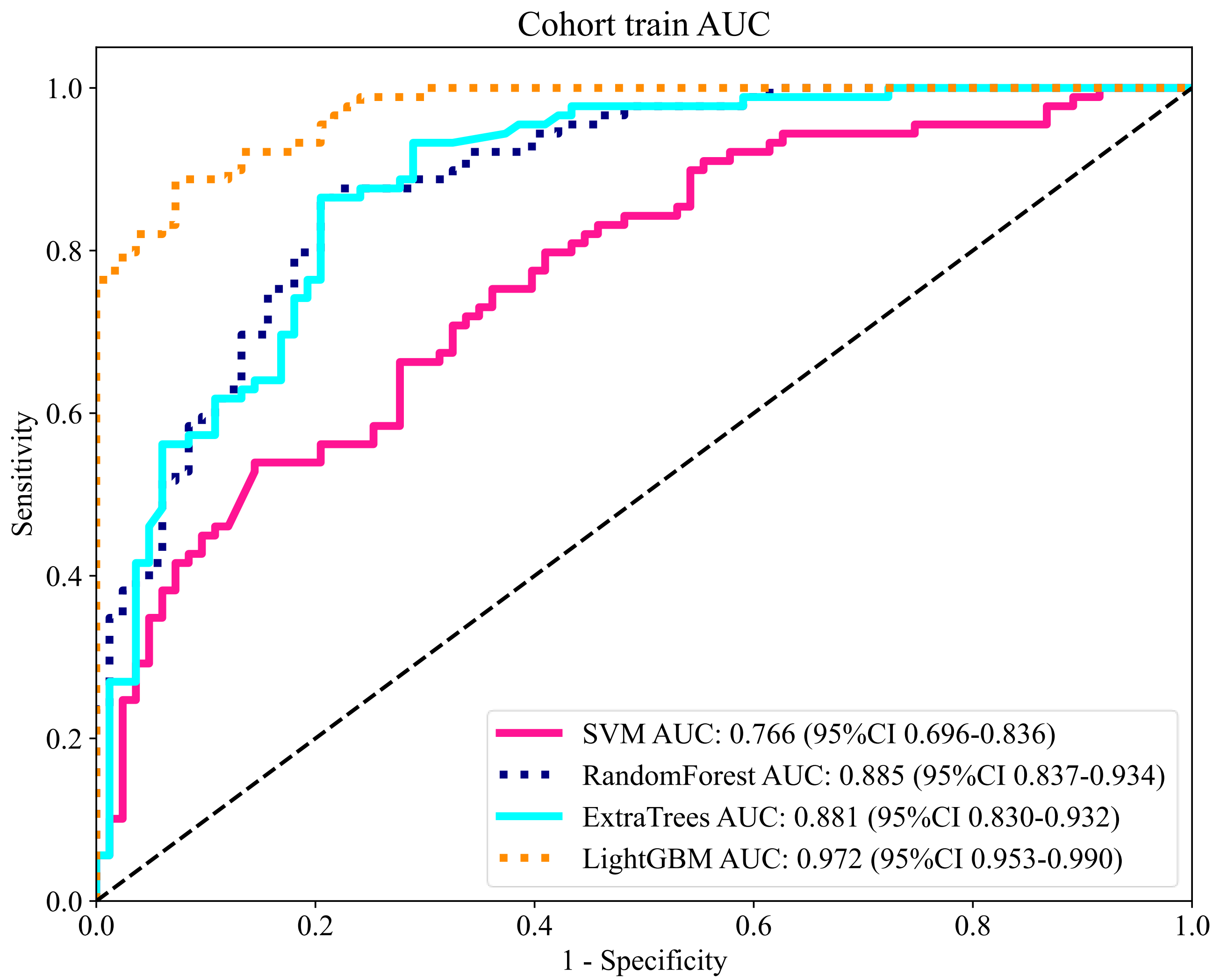
Cohort train AUC
1.0
0.8
0.6
0.4
0.2
0.0
Sensitivity
0.0
0.2
0.4
0.6
0.8
1.0
1 - Specificity
SVM AUC: 0.766 (95%CI 0.696-0.836)
RandomForest AUC: 0.885 (95%CI 0.837-0.934)
ExtraTrees AUC: 0.881 (95%CI 0.830-0.932)
LightGBM AUC: 0.972 (95%CI 0.953-0.990)

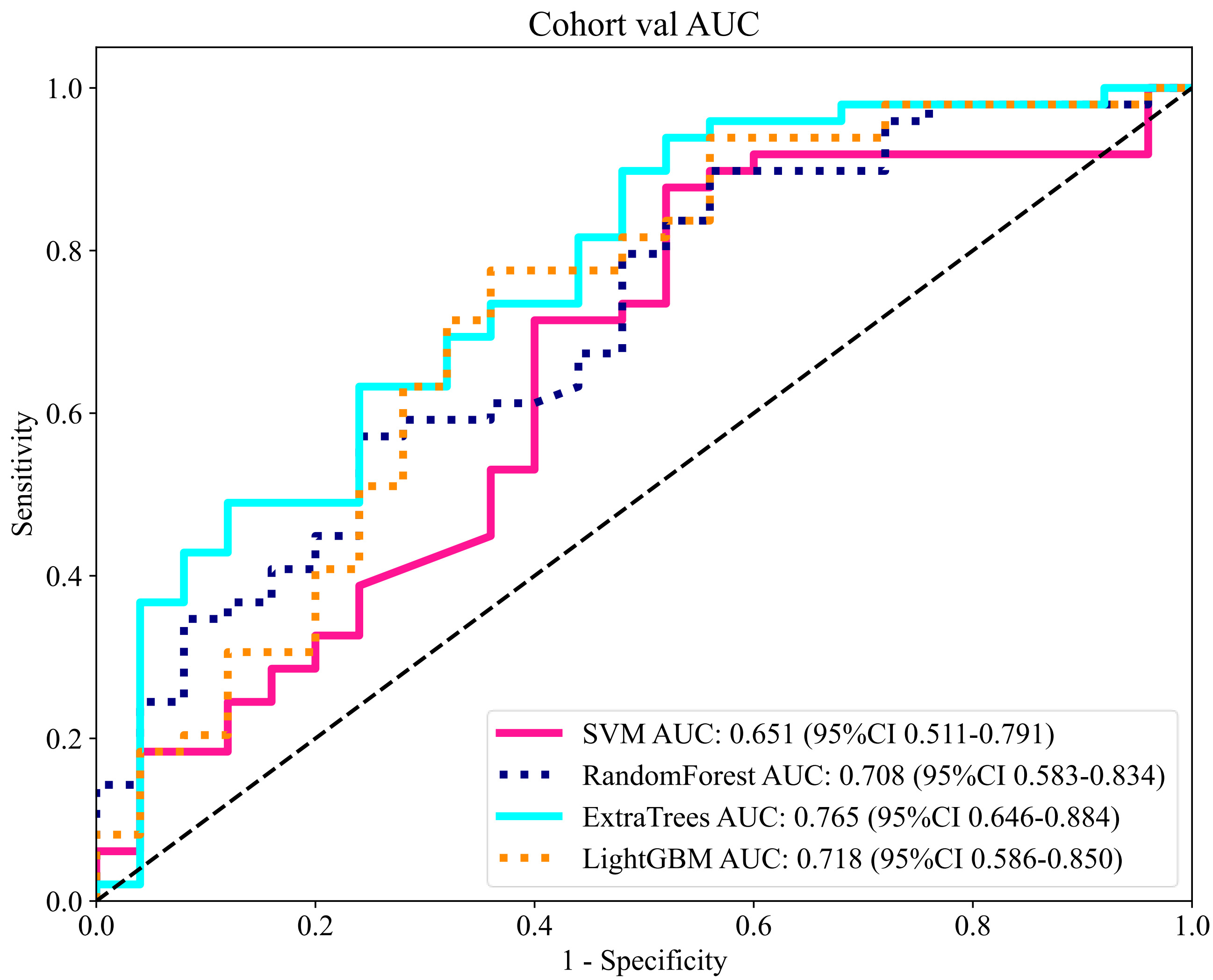
Cohort val AUC
1.0
0.8
0.6
0.4
0.2
0.0
Sensitivity
0.0
0.2
0.4
0.6
0.8
1.0
1 - Specificity
SVM AUC: 0.651 (95%CI 0.511-0.791)
RandomForest AUC: 0.708 (95%CI 0.583-0.834)
ExtraTrees AUC: 0.765 (95%CI 0.646-0.884)
LightGBM AUC: 0.718 (95%CI 0.586-0.850)

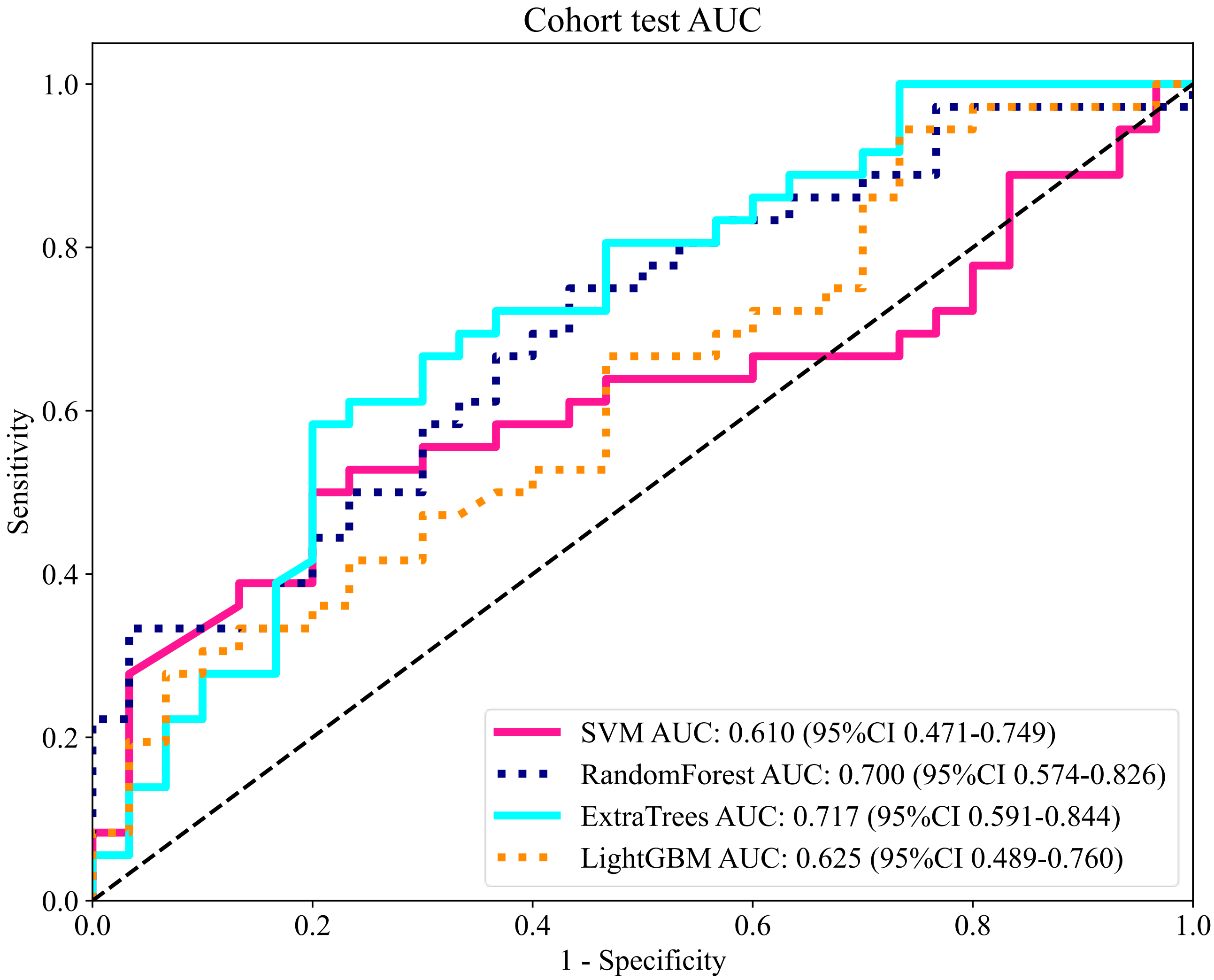

Figure S11. ROC curves of different machine learning models for Peri1mm.

Peri2mm

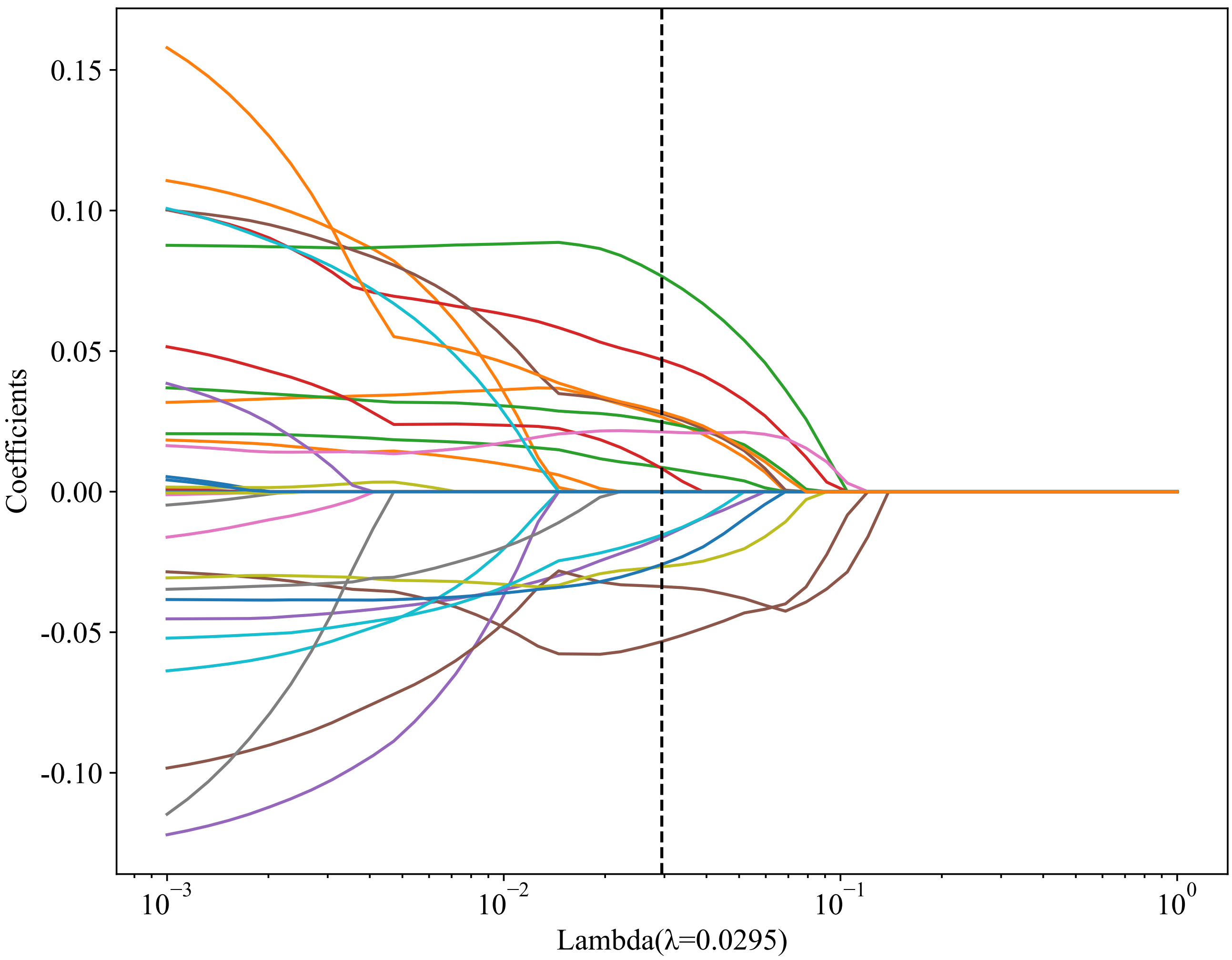

0.15
0.10
0.05
0.00
-0.05
-0.10
Coefficients
10^{-3}
10^{-2}
10^{-1}
10^{0}
Lambda(λ=0.0295)

a

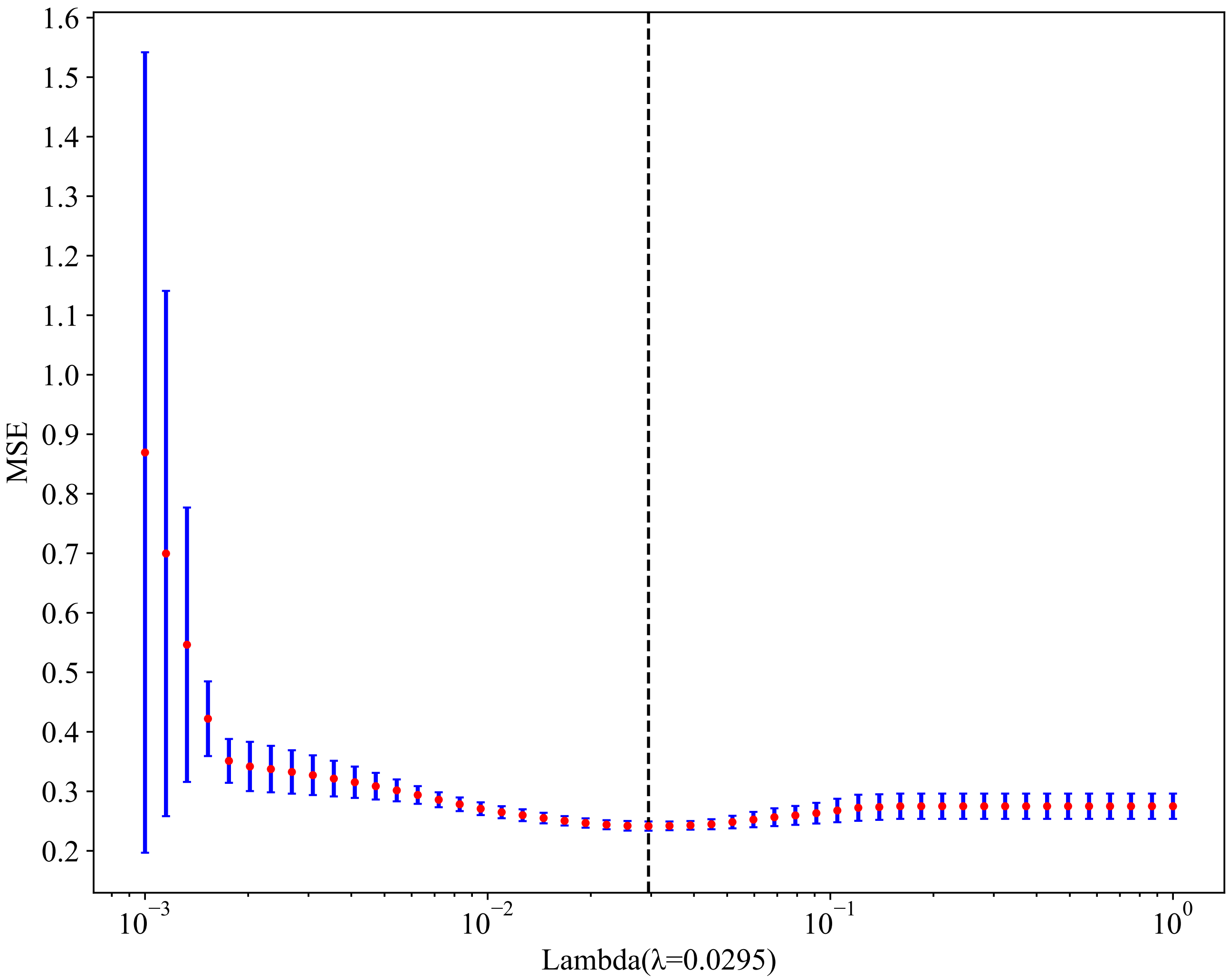

MSE
Lambda(λ=0.0295)

b

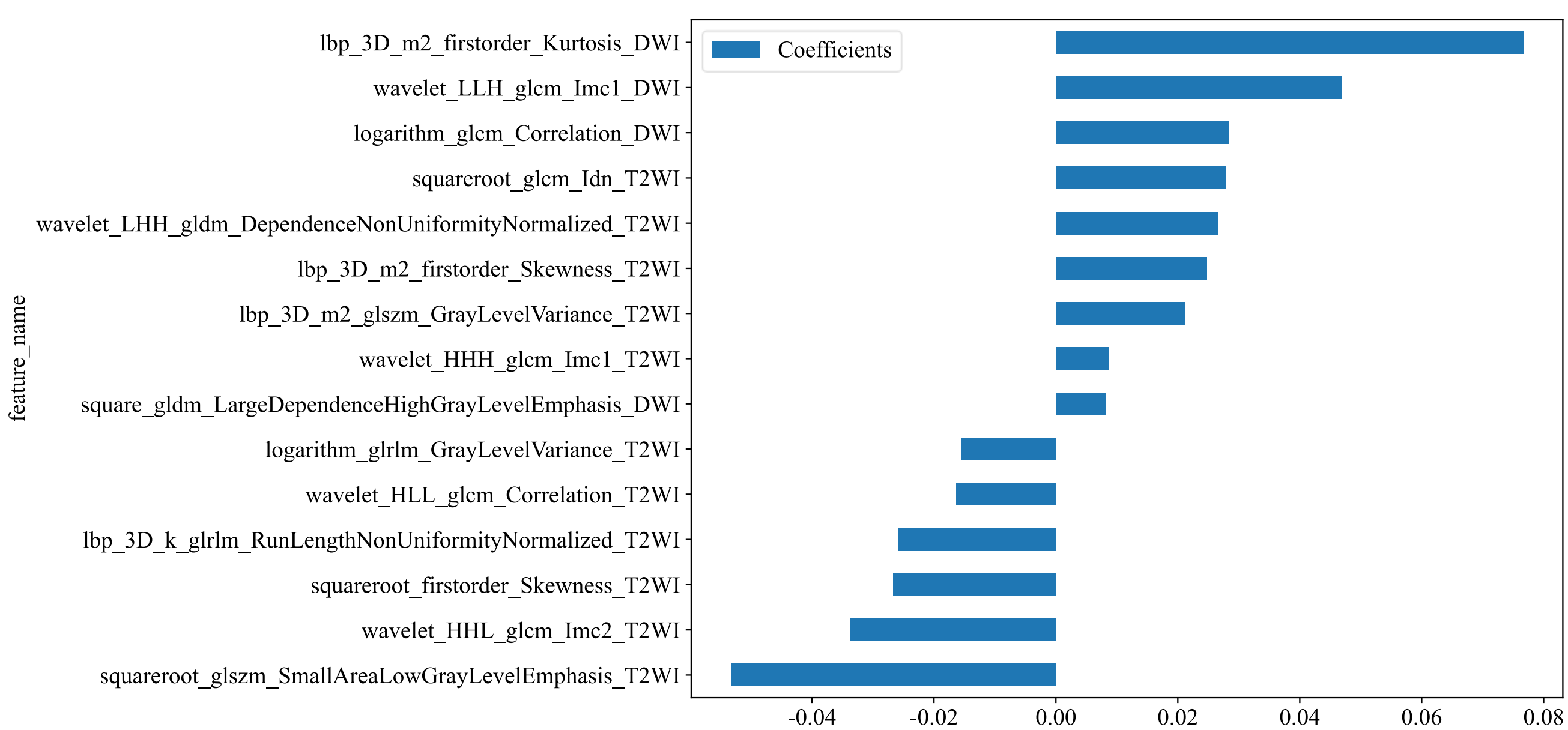

Coefficients
feature_name
lbp_3D_m2_firstorder_Kurtosis_DWI
wavelet_LLH_glcm_Imc1_DWI
logarithm_glcm_Correlation_DWI
squareroot_glcm_Idn_T2WI
wavelet_LHH_gldm_DependenceNonUniformityNormalized_T2WI
lbp_3D_m2_firstorder_Skewness_T2WI
lbp_3D_m2_glszm_GrayLevelVariance_T2WI
wavelet_HHH_glcm_Imc1_T2WI
square_gldm_LargeDependenceHighGrayLevelEmphasis_DWI
logarithm_glrlm_GrayLevelVariance_T2WI
wavelet_HLL_glcm_Correlation_T2WI
lbp_3D_k_glrlm_RunLengthNonUniformityNormalized_T2WI
squareroot_firstorder_Skewness_T2WI
wavelet_HHL_glcm_Imc2_T2WI
squareroot_glszm_SmallAreaLowGrayLevelEmphasis_T2WI

c

Figure S12. Coefficients of 10-fold cross-validation (a), MSE of 10-fold cross-validation (b), the histogram of the Rad-score based on the selected features (c) for Peri2mm.

model_name	Accuracy	AUC	95% CI	Sensitivity	Specificity	PPV	NPV	Cohort
SVM	0.674	0.711	0.634 - 0.788	0.472	0.892	0.824	0.612	train
SVM	0.703	0.667	0.533 - 0.801	0.673	0.760	0.846	0.543	val
SVM	0.697	0.708	0.579 - 0.838	0.778	0.600	0.700	0.692	test
RandomForest	0.820	0.896	0.851 - 0.942	0.921	0.711	0.774	0.894	train
RandomForest	0.730	0.727	0.598 - 0.856	0.755	0.680	0.822	0.586	val
RandomForest	0.697	0.761	0.644 - 0.878	0.472	0.967	0.944	0.604	test
ExtraTrees	0.767	0.837	0.778 - 0.895	0.742	0.795	0.795	0.742	train
ExtraTrees	0.770	0.798	0.682 - 0.914	0.776	0.760	0.864	0.633	val
ExtraTrees	0.667	0.720	0.597 - 0.844	0.389	1.000	1.000	0.577	test
LightGBM	0.709	0.851	0.794 - 0.907	0.506	0.928	0.882	0.636	train
LightGBM	0.689	0.738	0.608 - 0.869	0.633	0.800	0.861	0.526	val
LightGBM	0.667	0.710	0.582 - 0.838	0.500	0.867	0.818	0.591	test

Table S3. Model performance of different machine learning algorithms (Peri2mm).

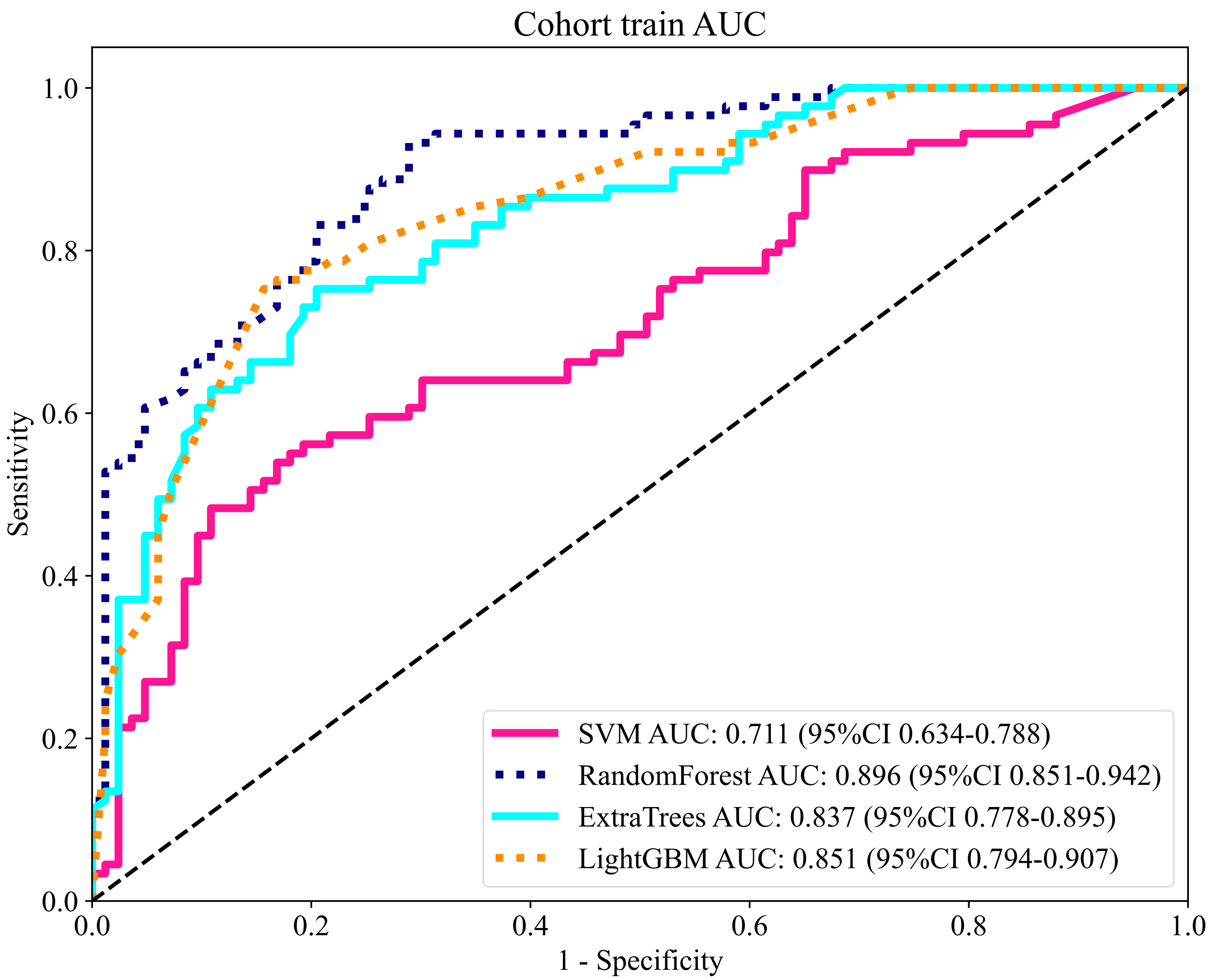
Cohort train AUC
1.0
0.8
0.6
0.4
0.2
0.0
Sensitivity
0.0
0.2
0.4
0.6
0.8
1.0
1 - Specificity
SVM AUC: 0.711 (95%CI 0.634-0.788)
RandomForest AUC: 0.896 (95%CI 0.851-0.942)
ExtraTrees AUC: 0.837 (95%CI 0.778-0.895)
LightGBM AUC: 0.851 (95%CI 0.794-0.907)

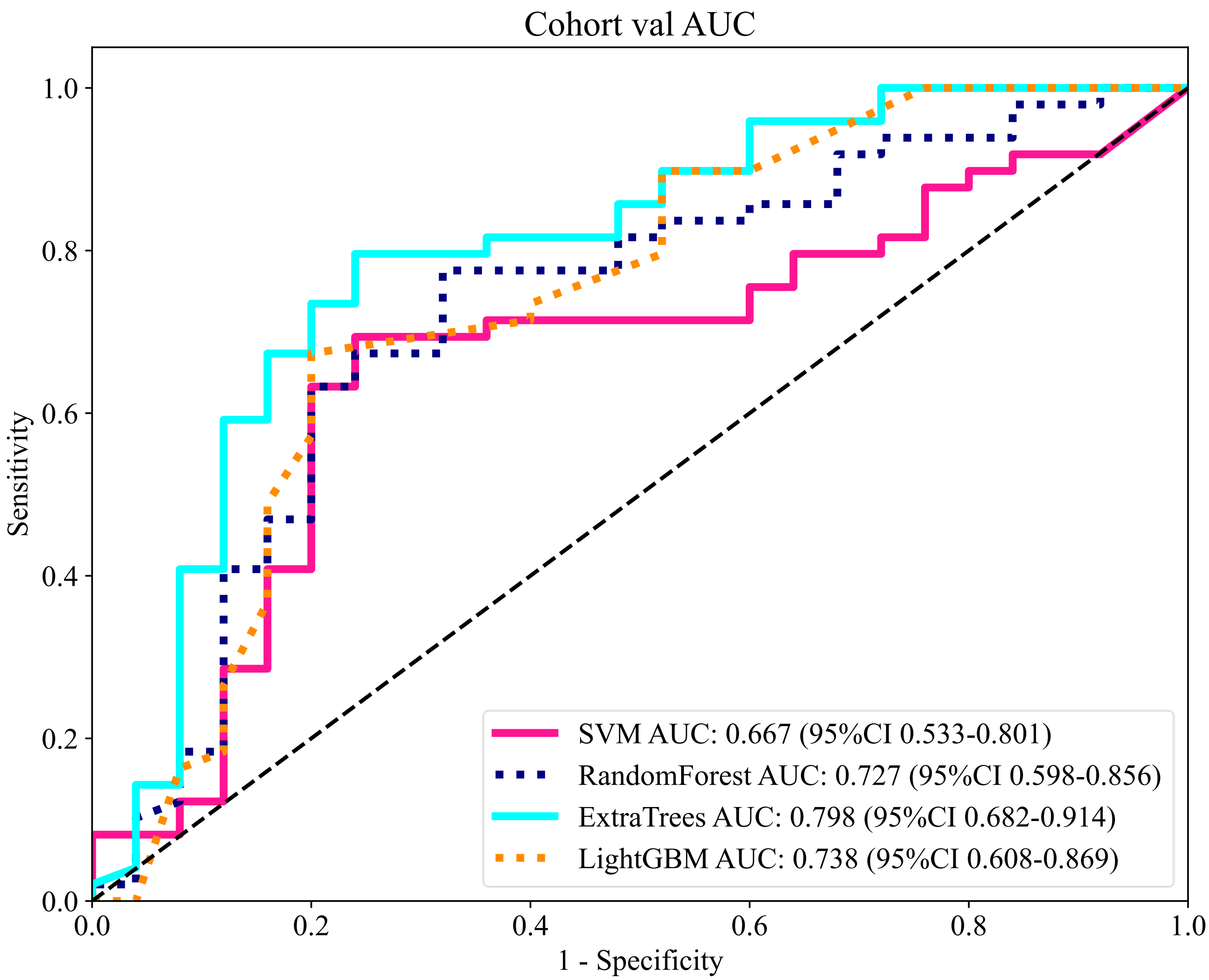
Cohort val AUC
1.0
0.8
0.6
0.4
0.2
0.0
Sensitivity
0.0
0.2
0.4
0.6
0.8
1.0
1 - Specificity
SVM AUC: 0.667 (95%CI 0.533-0.801)
RandomForest AUC: 0.727 (95%CI 0.598-0.856)
ExtraTrees AUC: 0.798 (95%CI 0.682-0.914)
LightGBM AUC: 0.738 (95%CI 0.608-0.869)

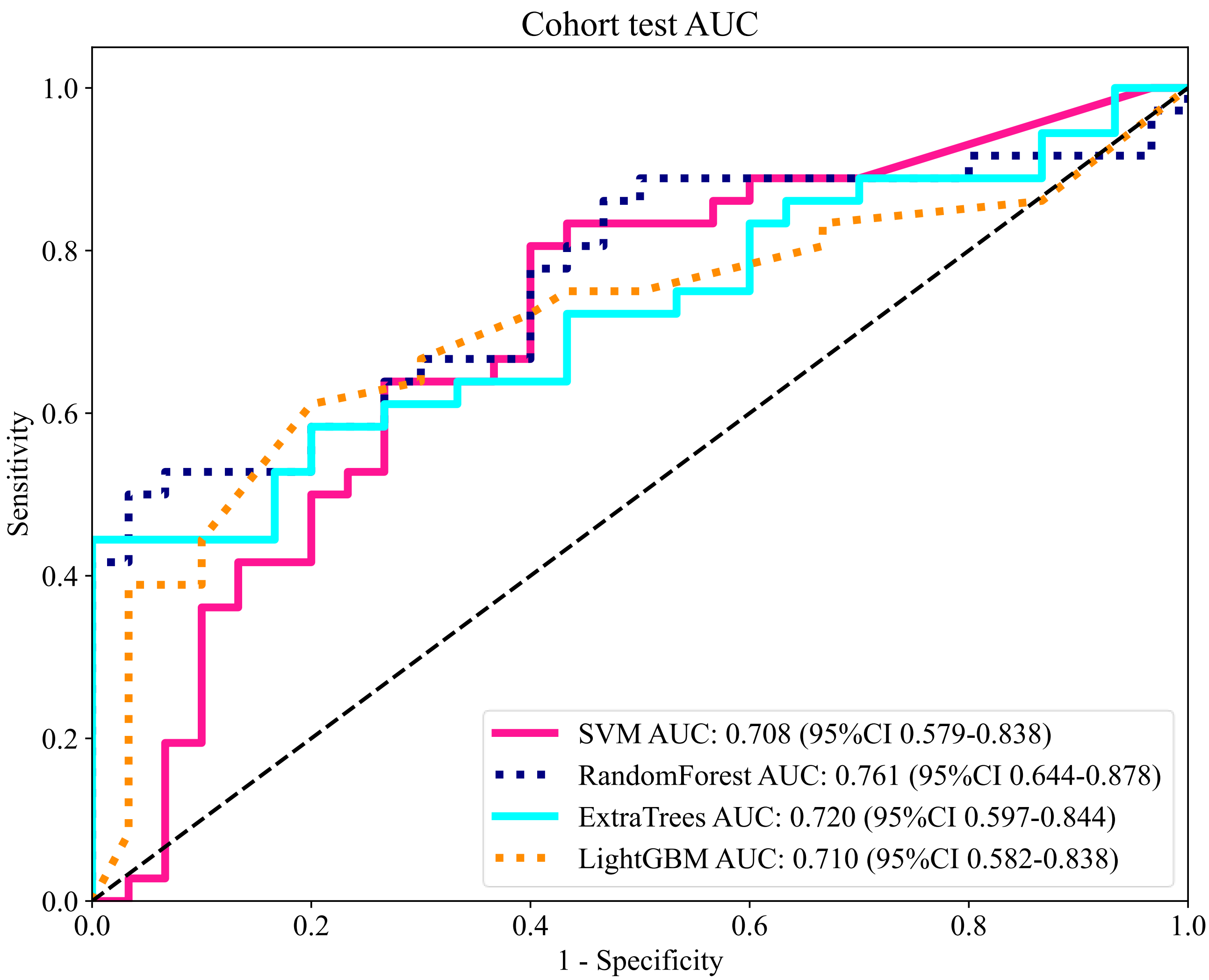

Figure S13. ROC curves of different machine learning models for Peri2mm.

Peri3mm

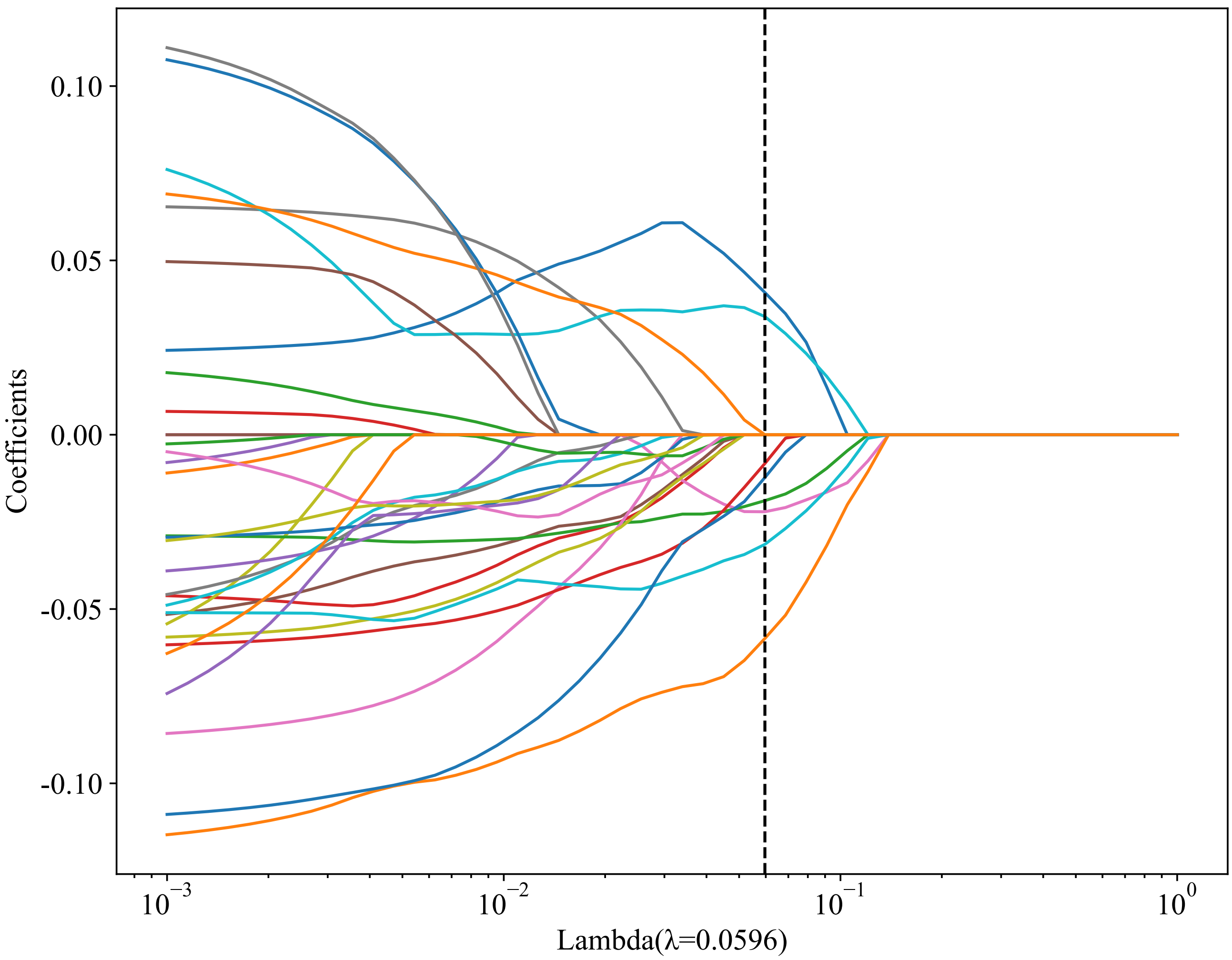

0.10
0.05
0.00
-0.05
-0.10
Coefficients
10^{-3}
10^{-2}
10^{-1}
10^{0}
Lambda(λ=0.0596)

a

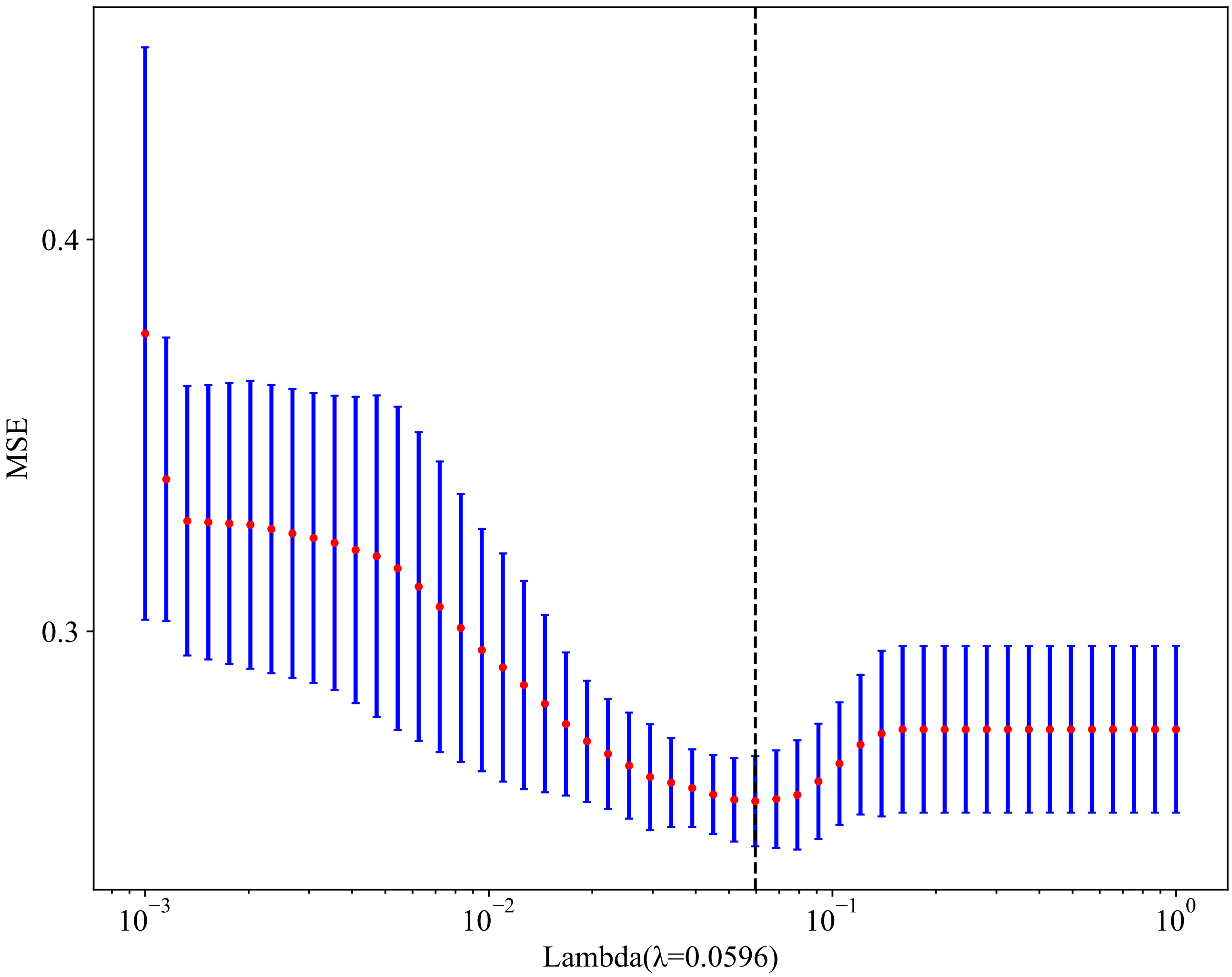

b

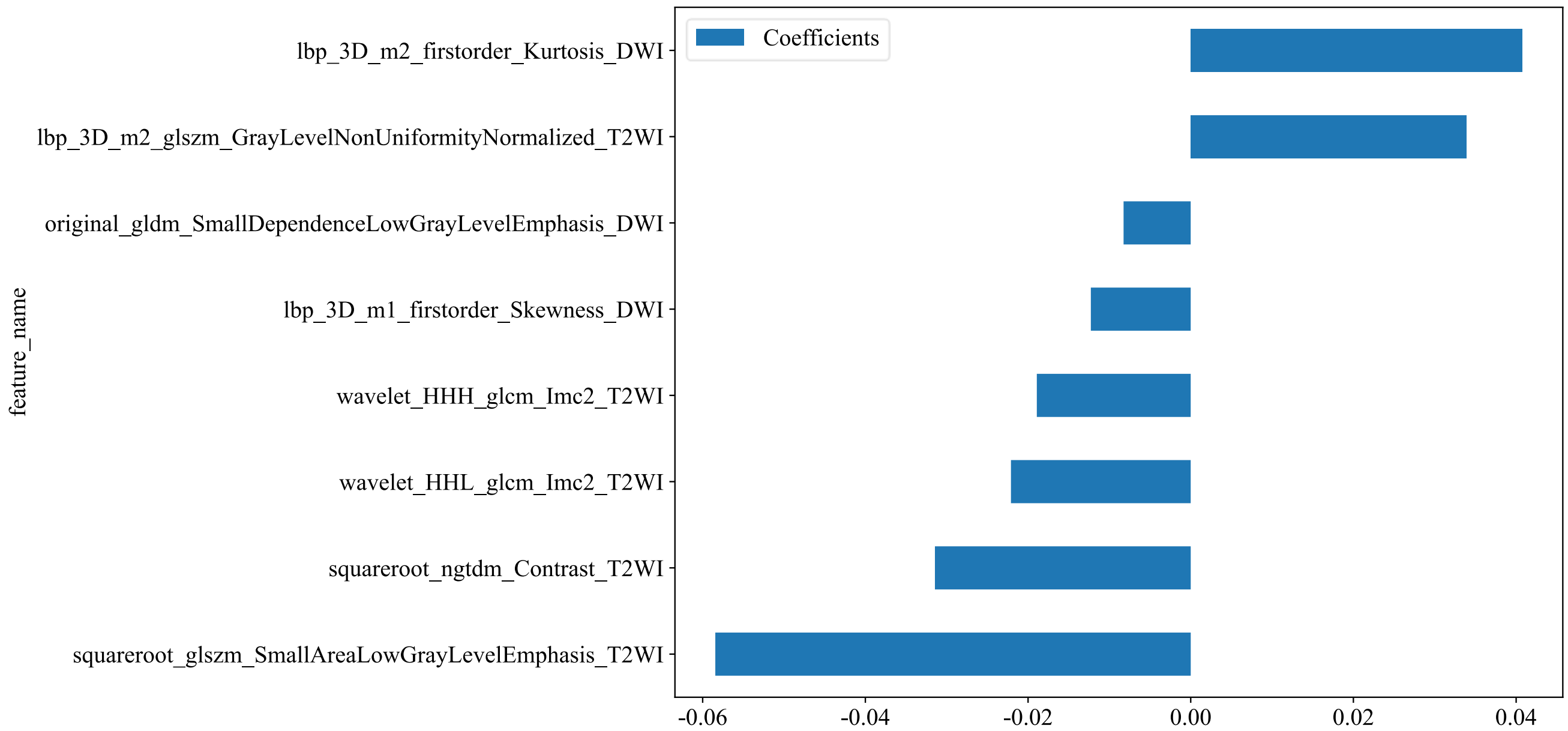

c

Figure S14. Coefficients of 10-fold cross-validation (a), MSE of 10-fold cross-validation (b), the histogram of the Rad-score based on the selected features (c) for Peri3mm.

model_name	Accuracy	AUC	95% CI	Sensitivity	Specificity	PPV	NPV	Cohort
SVM	0.645	0.687	0.609 - 0.766	0.764	0.518	0.630	0.672	train
SVM	0.635	0.599	0.457 - 0.741	0.653	0.600	0.762	0.469	val
SVM	0.667	0.666	0.532 - 0.799	0.528	0.833	0.792	0.595	test
RandomForest	0.802	0.883	0.834 - 0.931	0.798	0.807	0.816	0.788	train
RandomForest	0.770	0.740	0.605 - 0.874	0.837	0.640	0.820	0.667	val
RandomForest	0.682	0.710	0.585 - 0.835	0.694	0.667	0.714	0.645	test
ExtraTrees	0.692	0.768	0.699 - 0.837	0.618	0.771	0.743	0.653	train
ExtraTrees	0.635	0.718	0.584 - 0.853	0.653	0.600	0.762	0.469	val
ExtraTrees	0.591	0.619	0.480 - 0.758	0.417	0.800	0.714	0.533	test
LightGBM	0.797	0.885	0.836 - 0.933	0.764	0.831	0.829	0.767	train

model_name	Accuracy	AUC	95% CI	Sensitivity	Specificity	PPV	NPV	Cohort
LightGBM	0.730	0.729	0.596 - 0.862	0.735	0.720	0.837	0.581	val
LightGBM	0.667	0.702	0.576 - 0.828	0.639	0.700	0.719	0.618	test

Table S4. Model performance of different machine learning algorithms (Peri3mm).

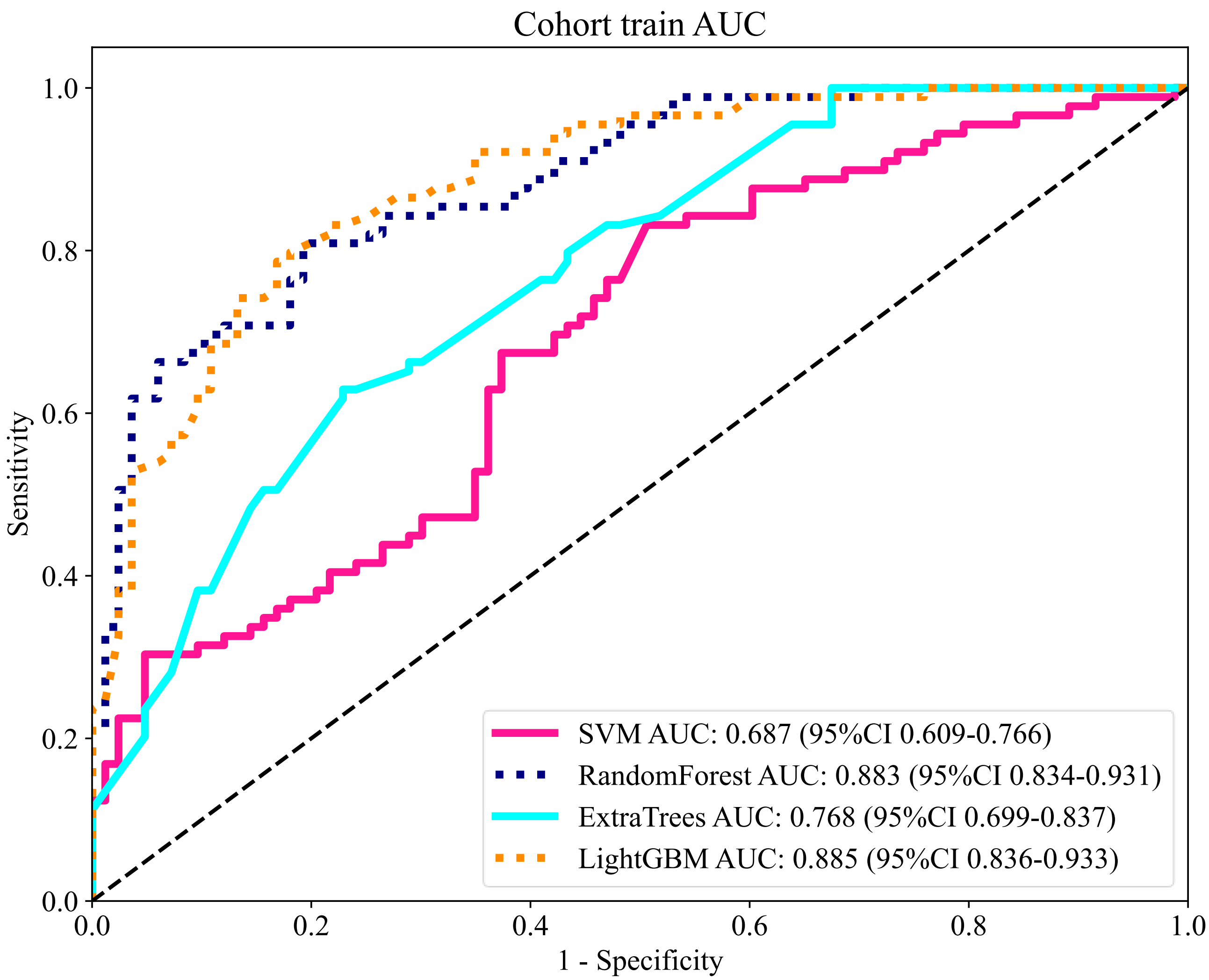
Cohort train AUC
1.0
0.8
0.6
0.4
0.2
0.0
Sensitivity
0.0
0.2
0.4
0.6
0.8
1.0
1 - Specificity
SVM AUC: 0.687 (95%CI 0.609-0.766)
RandomForest AUC: 0.883 (95%CI 0.834-0.931)
ExtraTrees AUC: 0.768 (95%CI 0.699-0.837)
LightGBM AUC: 0.885 (95%CI 0.836-0.933)

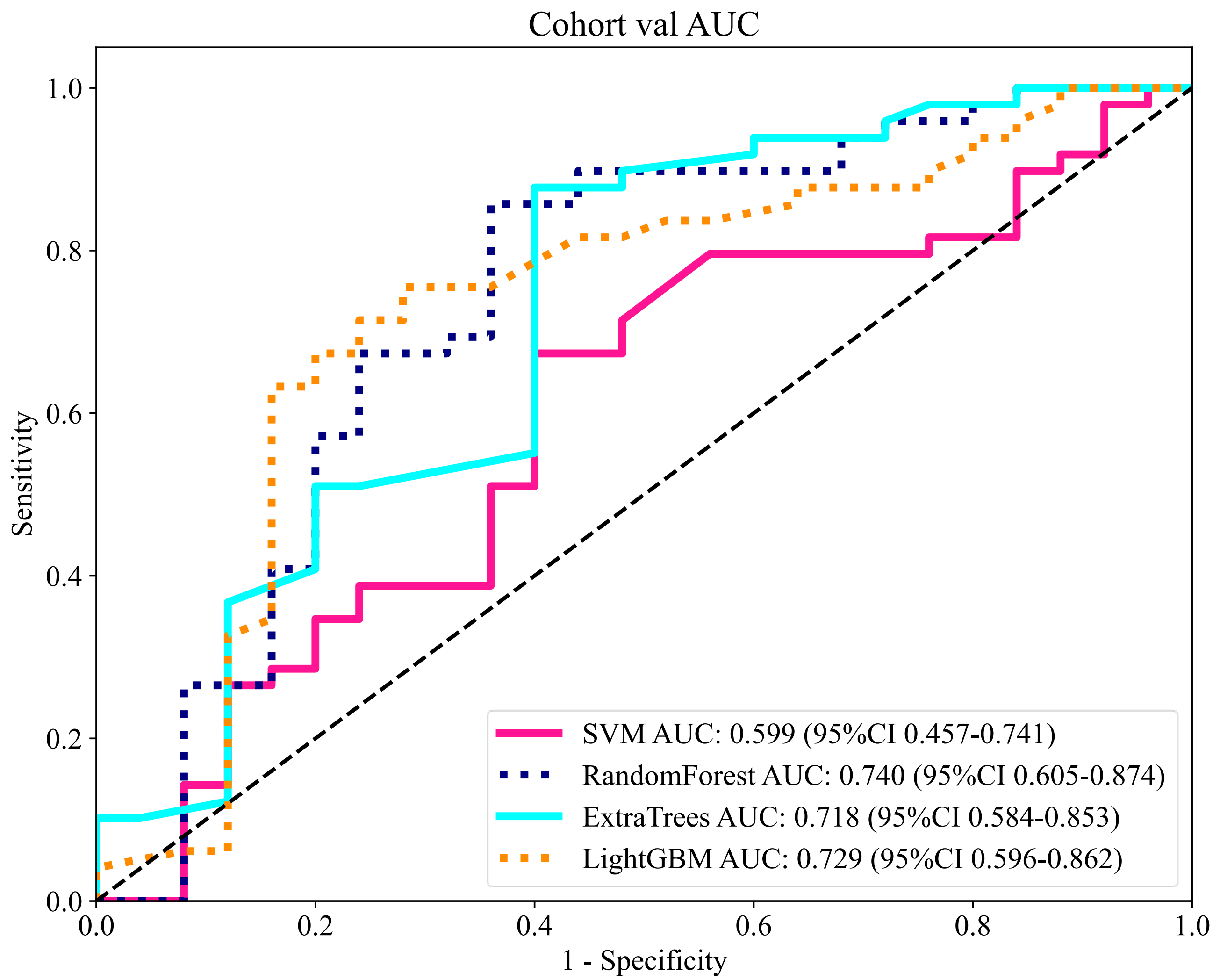
Cohort val AUC
Sensitivity
1 - Specificity
0.0
0.2
0.4
0.6
0.8
1.0
SVM AUC: 0.599 (95%CI 0.457-0.741)
RandomForest AUC: 0.740 (95%CI 0.605-0.874)
ExtraTrees AUC: 0.718 (95%CI 0.584-0.853)
LightGBM AUC: 0.729 (95%CI 0.596-0.862)

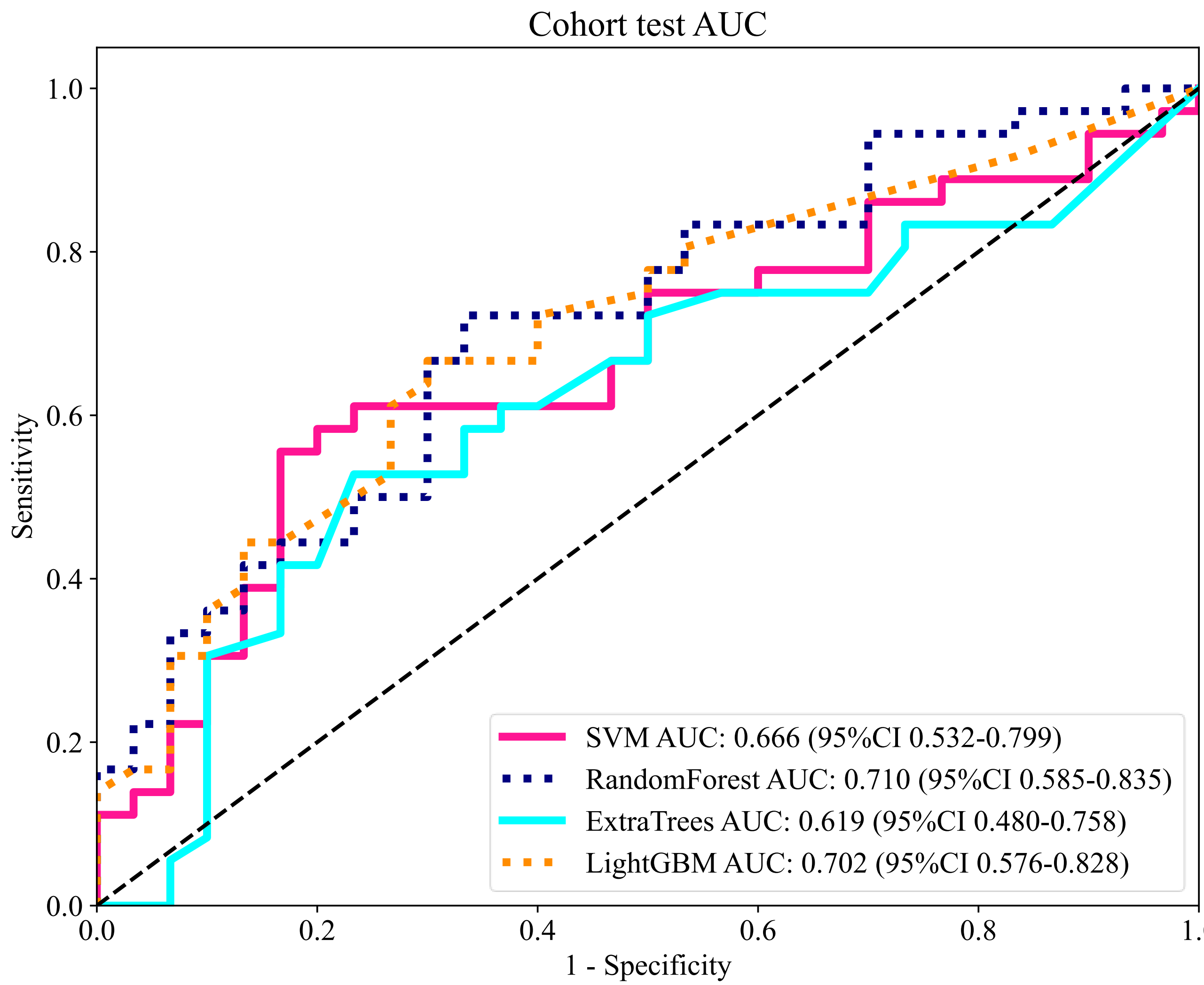

Figure S15. ROC curves of different machine learning models for Peri3mm.

Peri4mm

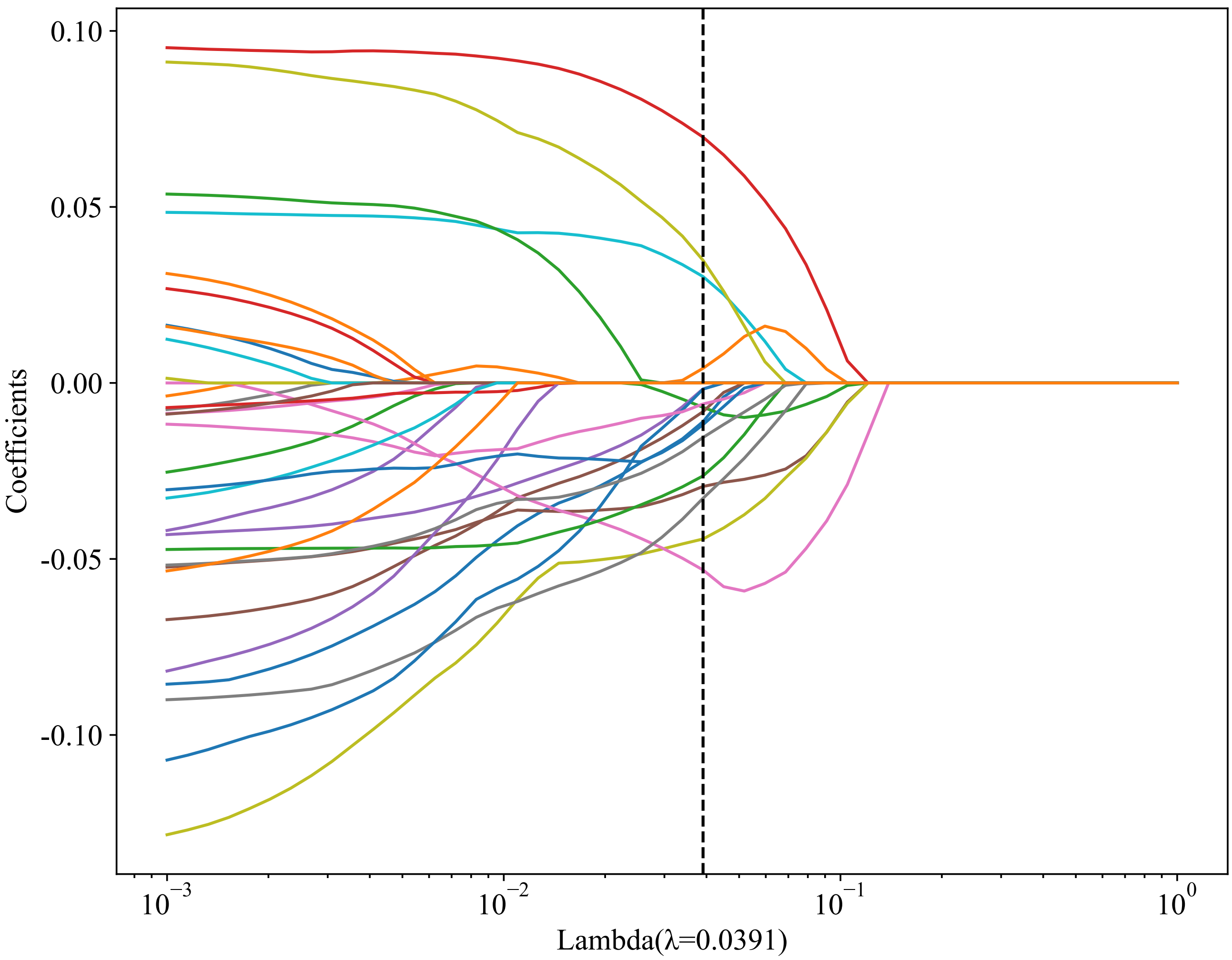

0.10
0.05
0.00
-0.05
-0.10
Coefficients
10^{-3}
10^{-2}
10^{-1}
10^{0}
Lambda(λ=0.0391)

a

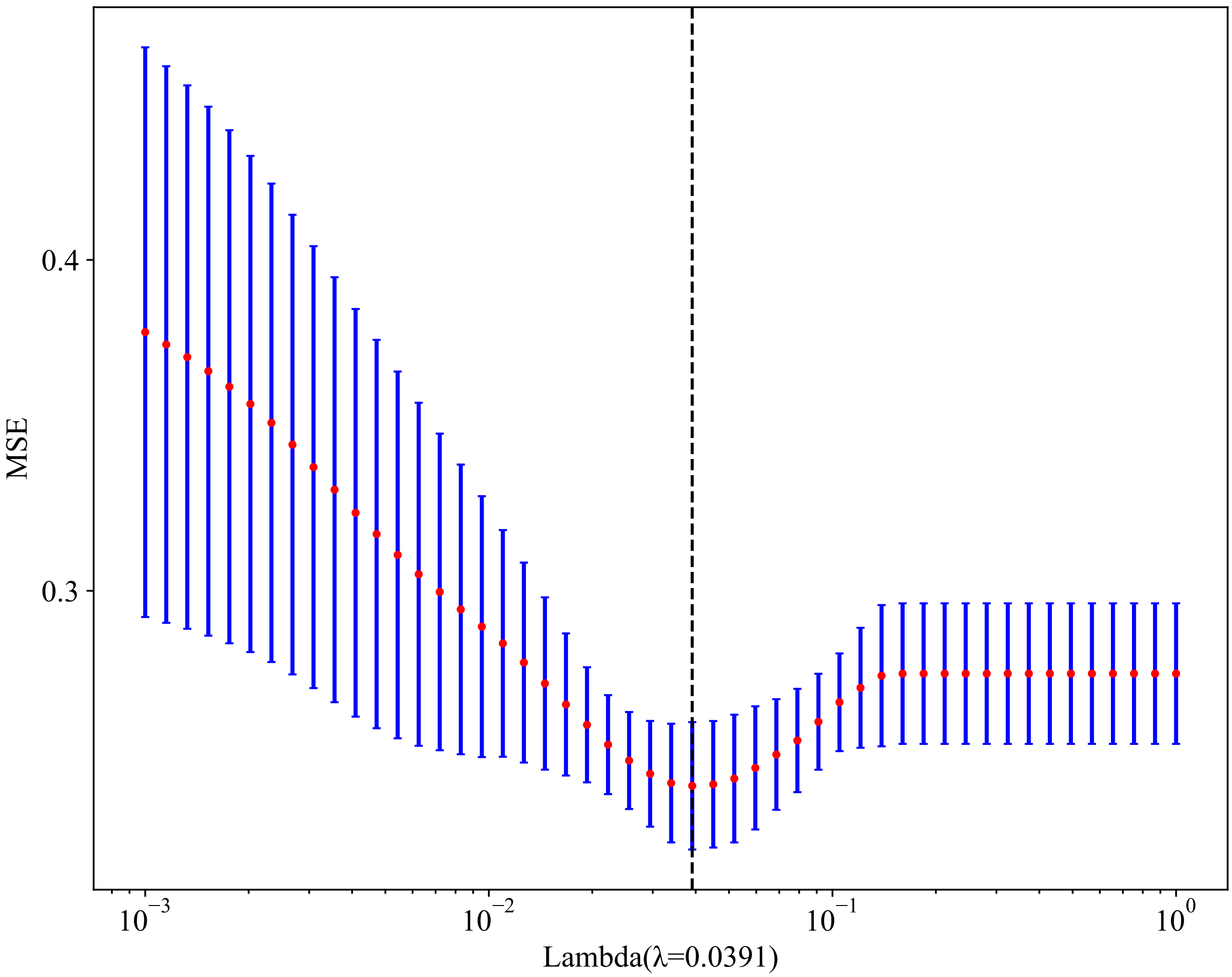
MSE
0.4
0.3
10^{-3}
10^{-2}
10^{-1}
10^{0}
Lambda(λ=0.0391)

b

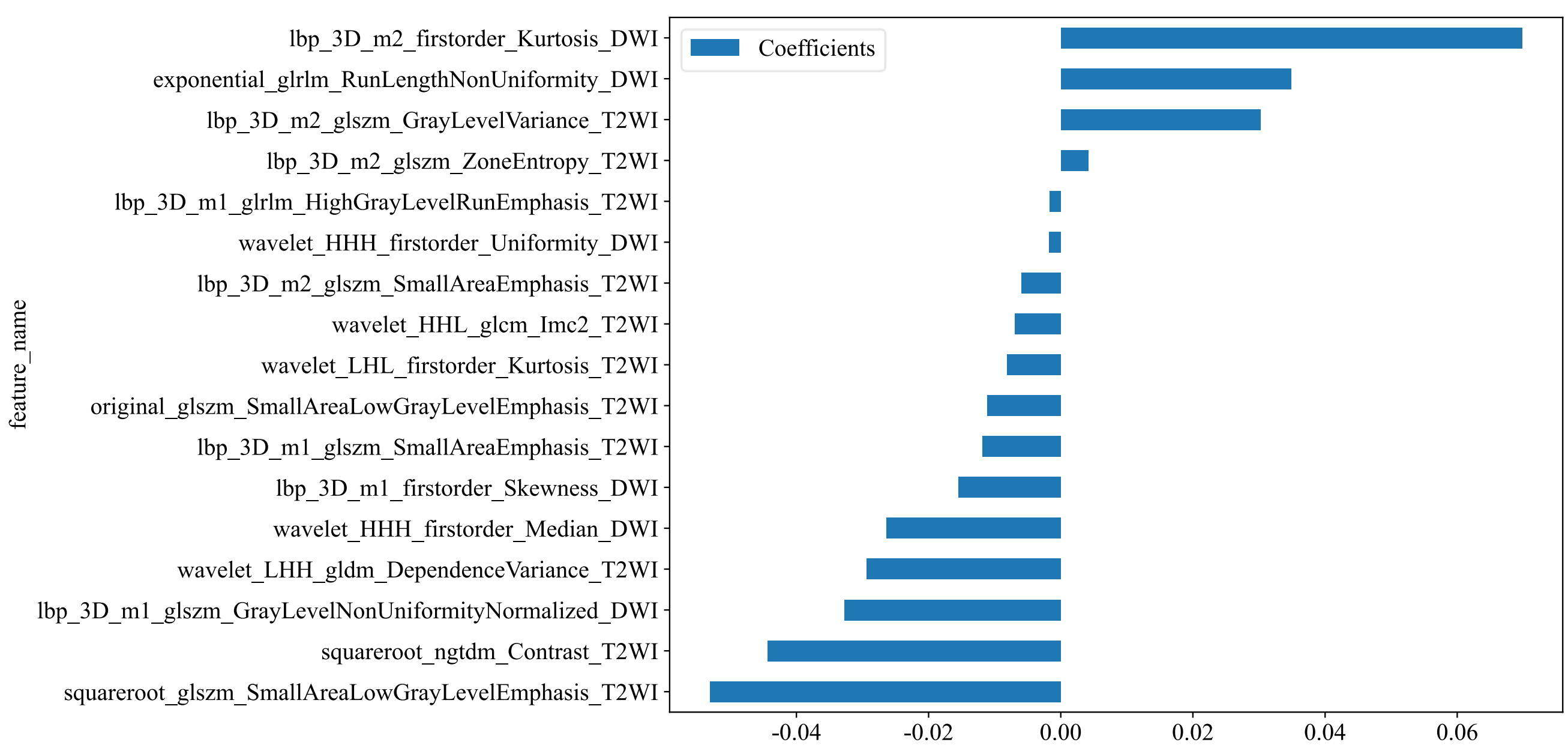
feature_name
Coefficients
lbp_3D_m2_firstorder_Kurtosis_DWI
exponential_glrlm_RunLengthNonUniformity_DWI
lbp_3D_m2_glszm_GrayLevelVariance_T2WI
lbp_3D_m2_glszm_ZoneEntropy_T2WI
lbp_3D_m1_glrlm_HighGrayLevelRunEmphasis_T2WI
wavelet_HHH_firstorder_Uniformity_DWI
lbp_3D_m2_glszm_SmallAreaEmphasis_T2WI
wavelet_HHL_glcm_Imc2_T2WI
wavelet_LHL_firstorder_Kurtosis_T2WI
original_glszm_SmallAreaLowGrayLevelEmphasis_T2WI
lbp_3D_m1_glszm_SmallAreaEmphasis_T2WI
lbp_3D_m1_firstorder_Skewness_DWI
wavelet_HHH_firstorder_Median_DWI
wavelet_LHH_gldm_DependenceVariance_T2WI
lbp_3D_m1_glszm_GrayLevelNonUniformityNormalized_DWI
squareroot_ngtdm_Contrast_T2WI
squareroot_glszm_SmallAreaLowGrayLevelEmphasis_T2WI
-0.04
-0.02
0.00
0.02
0.04
0.06

c

Figure S16. Coefficients of 10-fold cross-validation (a), MSE of 10-fold cross-validation (b), the histogram of the Rad-score based on the selected features (c) for Peri4mm.

model_name	Accuracy	AUC	95% CI	Sensitivity	Specificity	PPV	NPV	Cohort
SVM	0.779	0.860	0.806 - 0.914	0.674	0.892	0.870	0.718	train
SVM	0.770	0.742	0.616 - 0.868	0.898	0.520	0.786	0.722	val
SVM	0.682	0.693	0.563 - 0.822	0.528	0.867	0.826	0.605	test
RandomForest	0.820	0.881	0.831 - 0.931	0.888	0.747	0.790	0.861	train
RandomForest	0.662	0.732	0.606 - 0.858	0.592	0.800	0.853	0.500	val
RandomForest	0.742	0.744	0.621 - 0.868	0.722	0.767	0.788	0.697	test
ExtraTrees	0.733	0.789	0.721 - 0.857	0.697	0.771	0.765	0.703	train
ExtraTrees	0.730	0.681	0.542 - 0.820	0.776	0.640	0.809	0.593	val
ExtraTrees	0.621	0.656	0.524 - 0.788	0.528	0.733	0.704	0.564	test
LightGBM	0.802	0.888	0.841 - 0.935	0.719	0.892	0.877	0.747	train
LightGBM	0.703	0.635	0.492 - 0.778	0.776	0.560	0.776	0.560	val
LightGBM	0.773	0.780	0.665 - 0.894	0.750	0.800	0.818	0.727	test

Table S5. Model performance of different machine learning algorithms (Peri4mm).

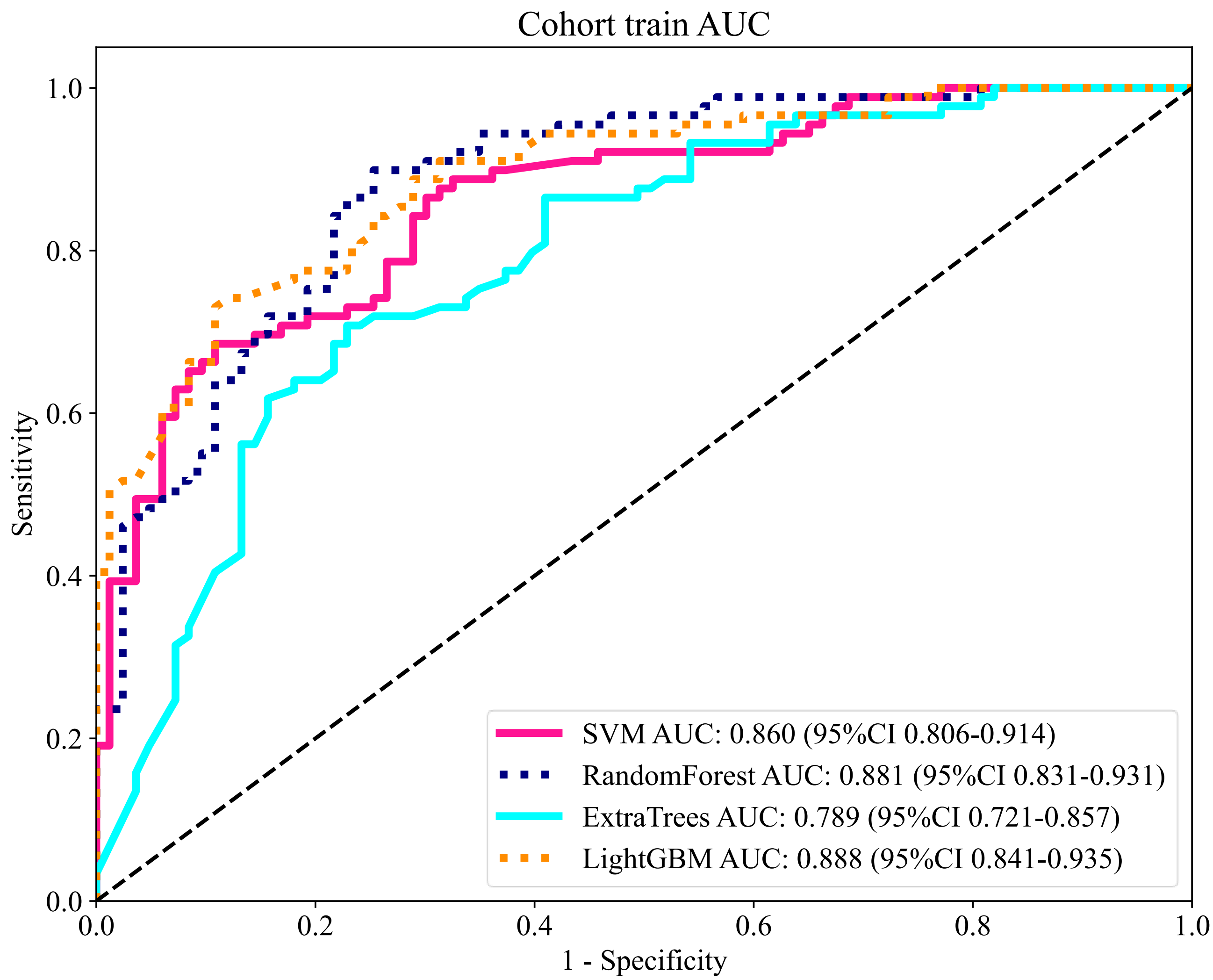

Cohort train AUC
Sensitivity
1 - Specificity
0.0
0.2
0.4
0.6
0.8
1.0
SVM AUC: 0.860 (95%CI 0.806-0.914)
RandomForest AUC: 0.881 (95%CI 0.831-0.931)
ExtraTrees AUC: 0.789 (95%CI 0.721-0.857)
LightGBM AUC: 0.888 (95%CI 0.841-0.935)

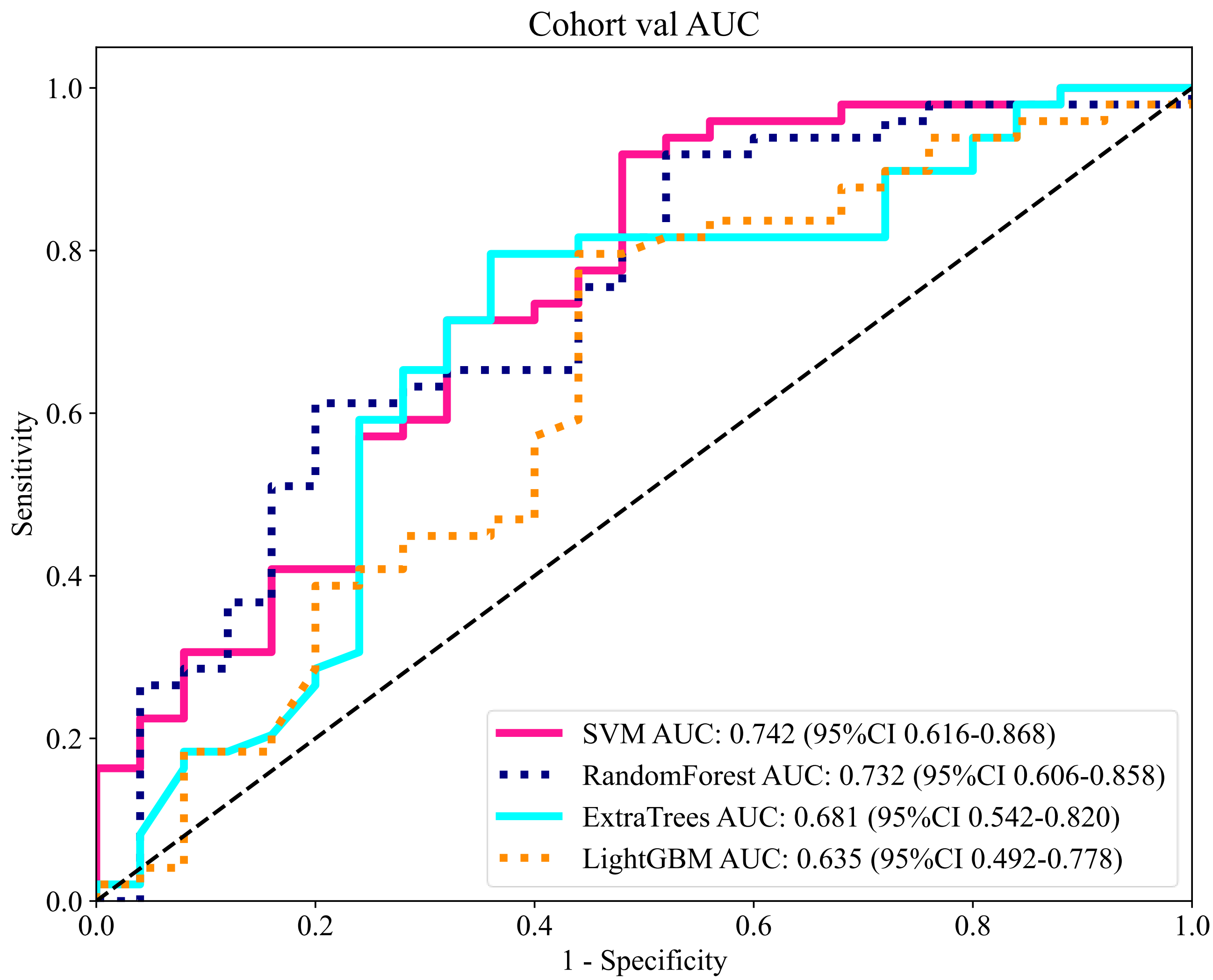

Cohort val AUC
Sensitivity
1 - Specificity
0.0
0.2
0.4
0.6
0.8
1.0
SVM AUC: 0.742 (95%CI 0.616-0.868)
RandomForest AUC: 0.732 (95%CI 0.606-0.858)
ExtraTrees AUC: 0.681 (95%CI 0.542-0.820)
LightGBM AUC: 0.635 (95%CI 0.492-0.778)

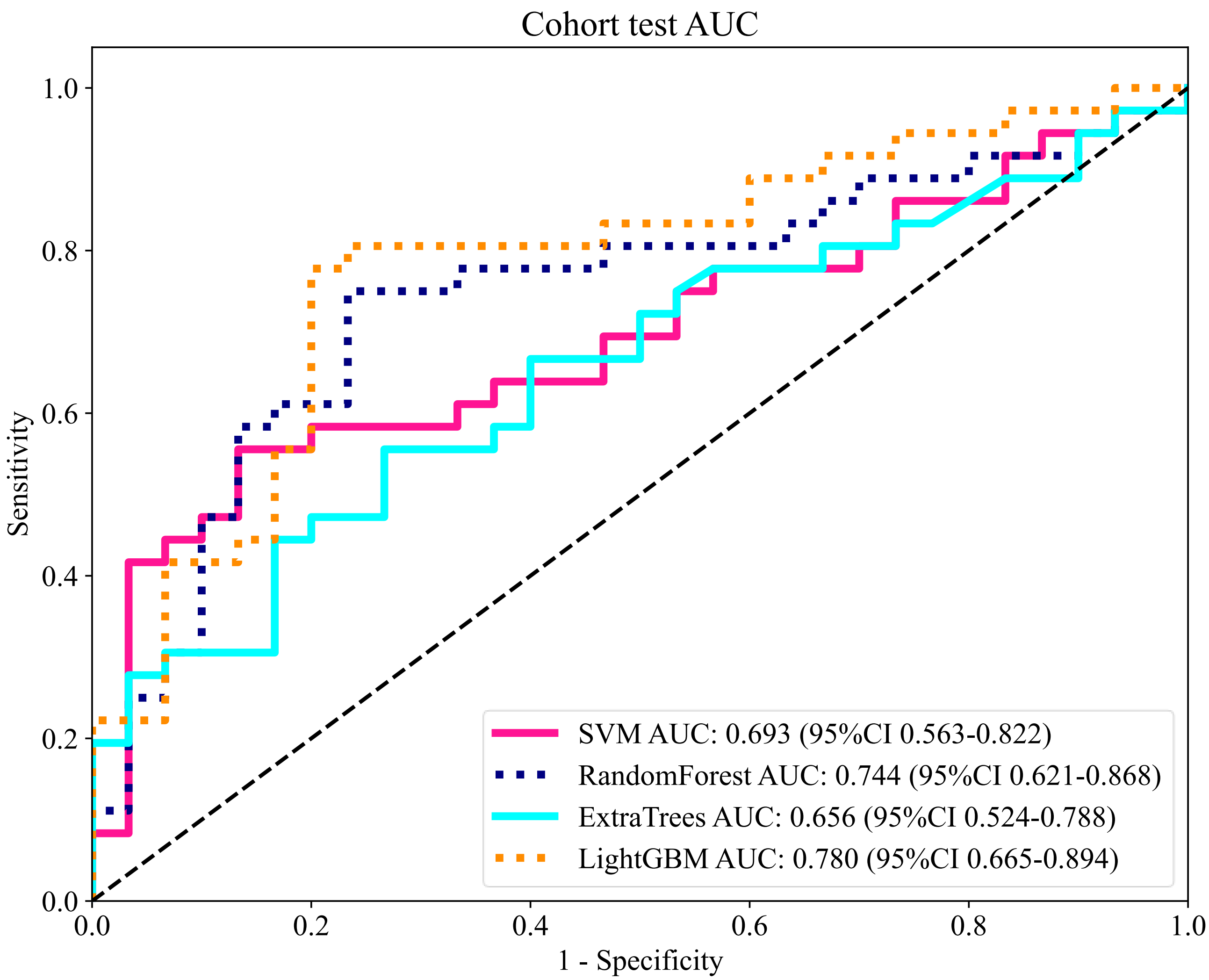

Figure S17. ROC curves of different machine learning models for Peri4mm.

Peri5mm

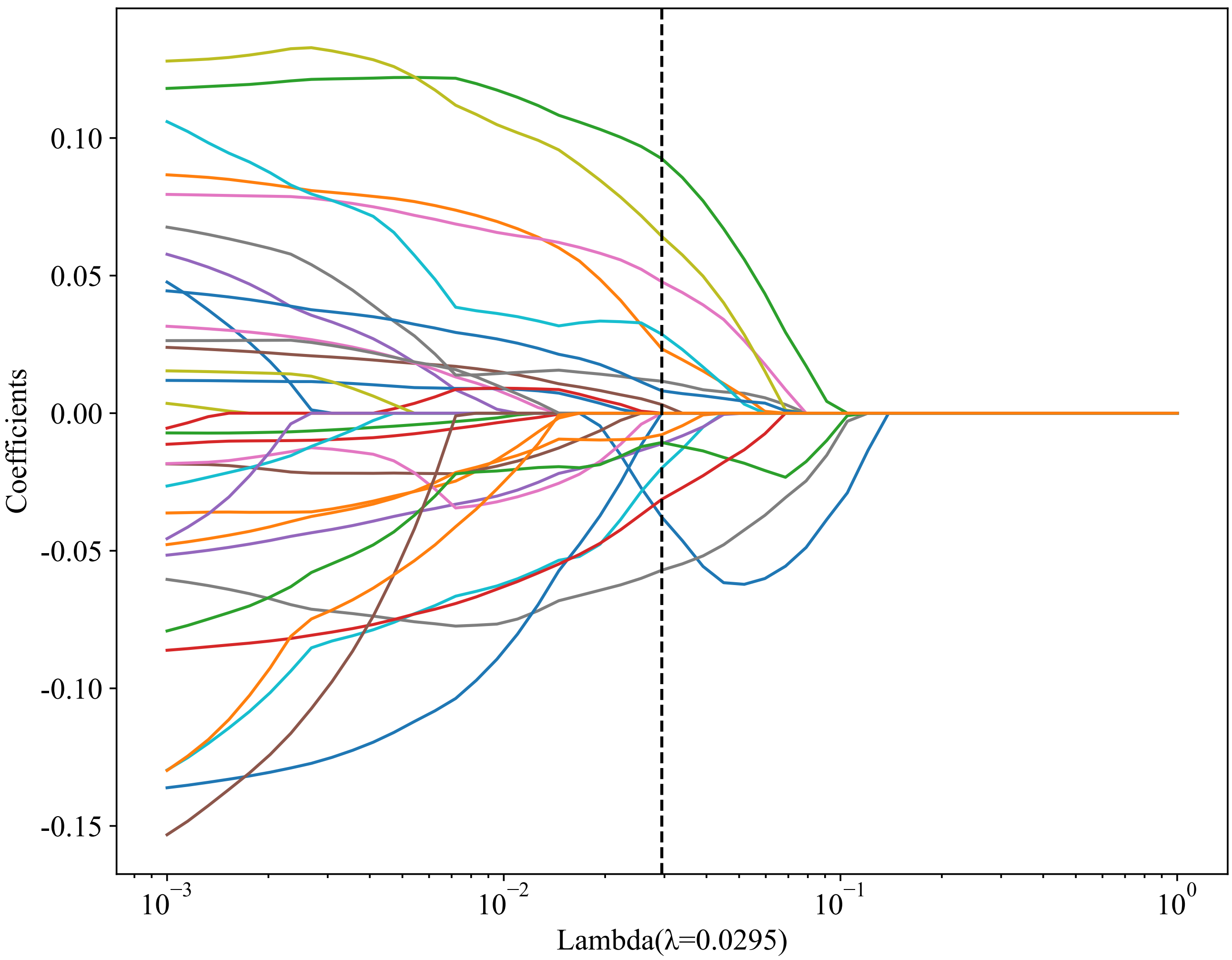

0.10
0.05
0.00
-0.05
-0.10
-0.15
Coefficients
10^{-3}
10^{-2}
10^{-1}
10^{0}
Lambda(λ=0.0295)

a

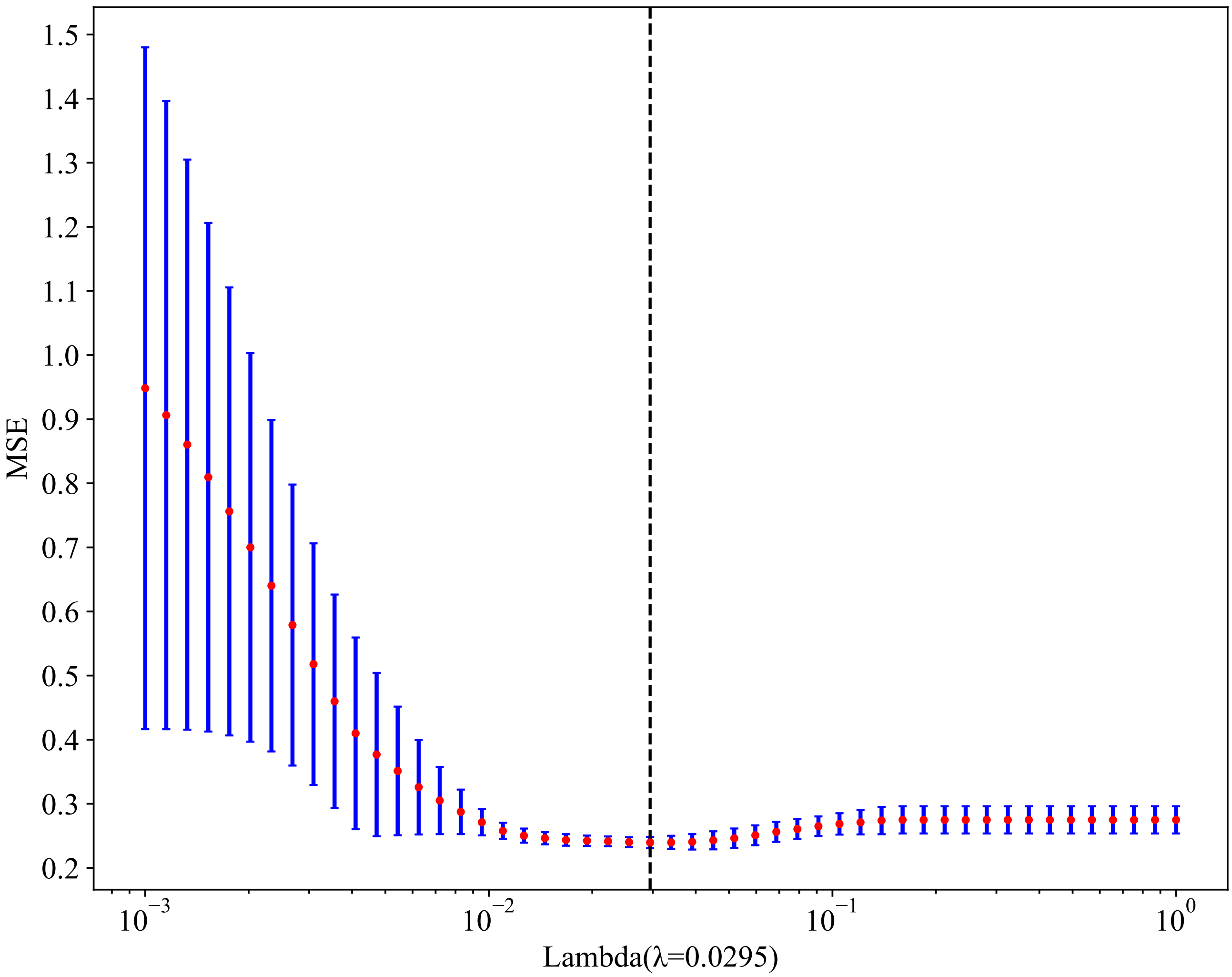
MSE
Lambda(λ=0.0295)
1.5
1.4
1.3
1.2
1.1
1.0
0.9
0.8
0.7
0.6
0.5
0.4
0.3
0.2
10^{-3}
10^{-2}
10^{-1}
10^{0}

b

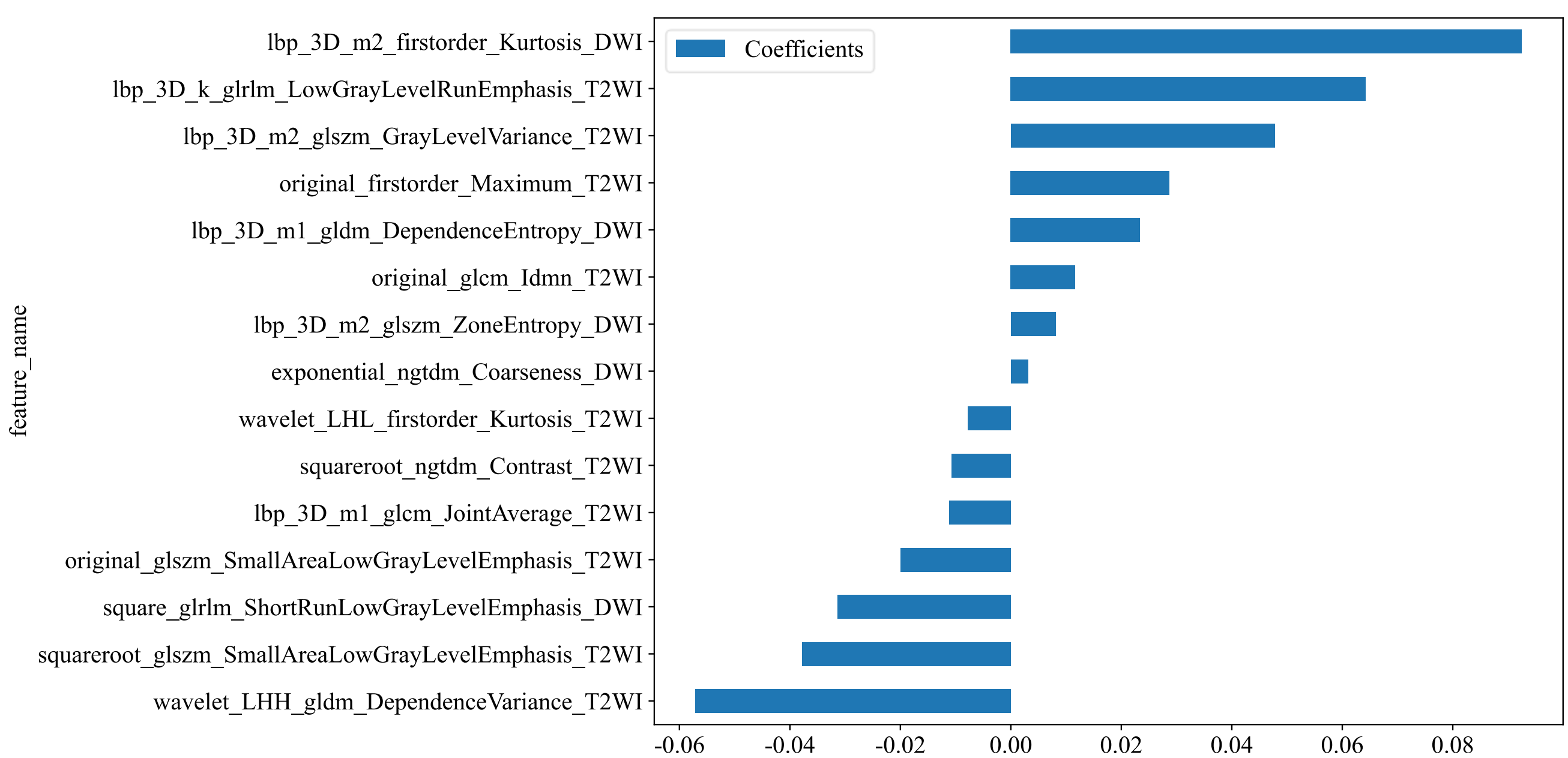
Coefficients
feature_name
lbp_3D_m2_firstorder_Kurtosis_DWI
lbp_3D_k_glrlm_LowGrayLevelRunEmphasis_T2WI
lbp_3D_m2_glszm_GrayLevelVariance_T2WI
original_firstorder_Maximum_T2WI
lbp_3D_m1_gldm_DependenceEntropy_DWI
original_glcm_Idmn_T2WI
lbp_3D_m2_glszm_ZoneEntropy_DWI
exponential_ngtdm_Coarseness_DWI
wavelet_LHL_firstorder_Kurtosis_T2WI
squareroot_ngtdm_Contrast_T2WI
lbp_3D_m1_glcm_JointAverage_T2WI
original_glszm_SmallAreaLowGrayLevelEmphasis_T2WI
square_glrlm_ShortRunLowGrayLevelEmphasis_DWI
squareroot_glszm_SmallAreaLowGrayLevelEmphasis_T2WI
wavelet_LHH_gldm_DependenceVariance_T2WI
-0.06
-0.04
-0.02
0.00
0.02
0.04
0.06
0.08

c

Figure S18. Coefficients of 10-fold cross-validation (a), MSE of 10-fold cross-validation (b), the histogram of the Rad-score based on the selected features (c) for Peri5mm.

model_name	Accuracy	AUC	95% CI	Sensitivity	Specificity	PPV	NPV	Cohort
SVM	0.657	0.709	0.634 - 0.784	0.461	0.867	0.788	0.600	train
SVM	0.608	0.697	0.578 - 0.817	0.490	0.840	0.857	0.457	val
SVM	0.591	0.639	0.504 - 0.774	0.278	0.967	0.909	0.527	test
RandomForest	0.785	0.856	0.800 - 0.911	0.798	0.771	0.789	0.780	train
RandomForest	0.662	0.751	0.637 - 0.865	0.592	0.800	0.853	0.500	val
RandomForest	0.606	0.635	0.500 - 0.770	0.333	0.933	0.857	0.538	test
ExtraTrees	0.802	0.876	0.826 - 0.926	0.775	0.831	0.831	0.775	train
ExtraTrees	0.703	0.720	0.598 - 0.842	0.735	0.640	0.800	0.552	val
ExtraTrees	0.667	0.666	0.530 - 0.801	0.833	0.467	0.652	0.700	test
XGBoost	0.994	1.000	1.000 - 1.000	0.989	1.000	1.000	0.988	train
XGBoost	0.676	0.752	0.637 - 0.867	0.653	0.720	0.821	0.514	val
XGBoost	0.667	0.658	0.523 - 0.794	0.472	0.900	0.850	0.587	test
LightGBM	0.762	0.841	0.783 - 0.898	0.697	0.831	0.816	0.719	train
LightGBM	0.689	0.731	0.602 - 0.860	0.633	0.800	0.861	0.526	val
LightGBM	0.652	0.669	0.537 - 0.802	0.694	0.600	0.676	0.621	test

Table S6. Model performance of different machine learning algorithms (Peri5mm).

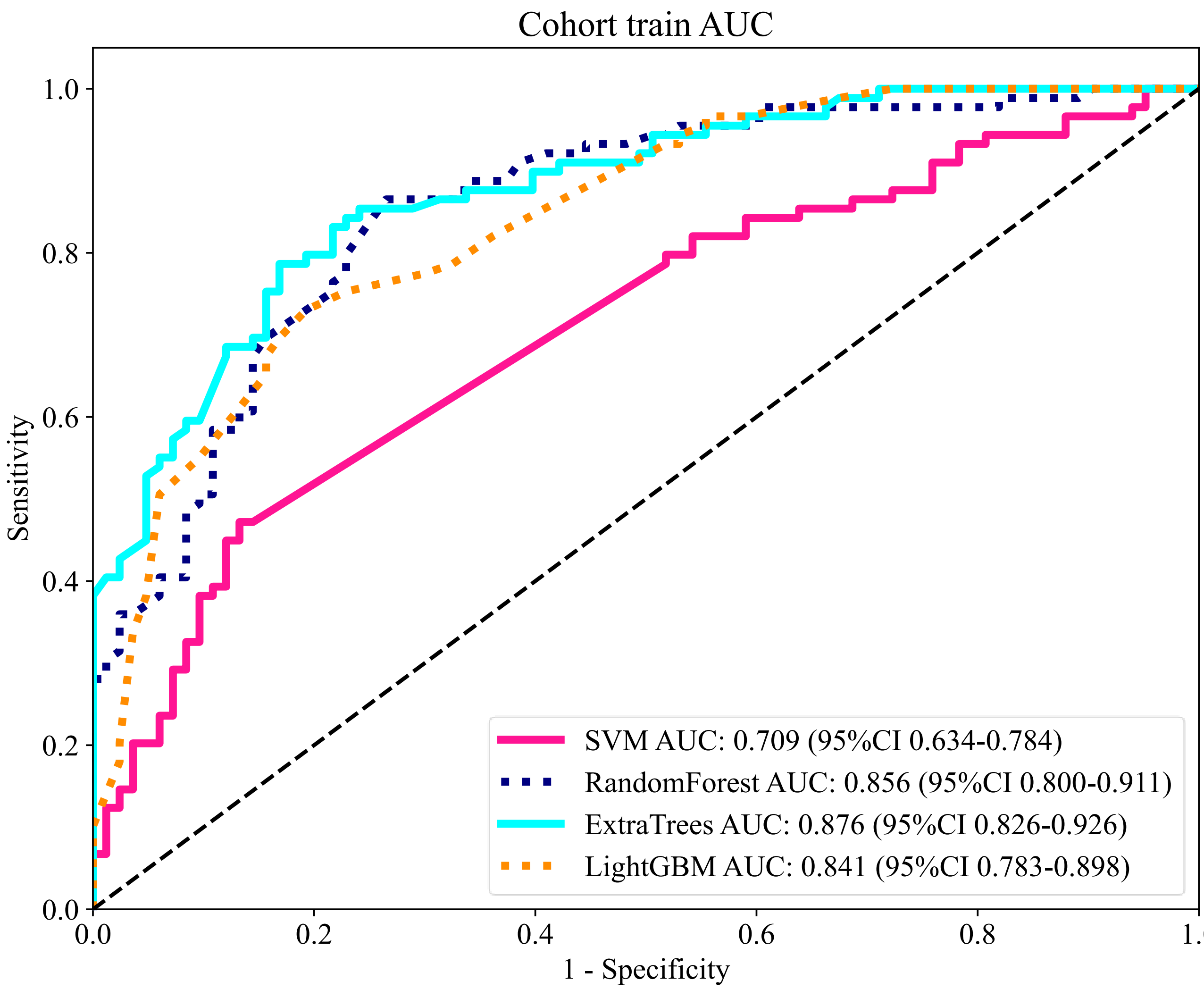
Cohort train AUC
1.0
0.8
0.6
0.4
0.2
0.0
Sensitivity
0.0
0.2
0.4
0.6
0.8
1.0
1 - Specificity
SVM AUC: 0.709 (95%CI 0.634-0.784)
RandomForest AUC: 0.856 (95%CI 0.800-0.911)
ExtraTrees AUC: 0.876 (95%CI 0.826-0.926)
LightGBM AUC: 0.841 (95%CI 0.783-0.898)

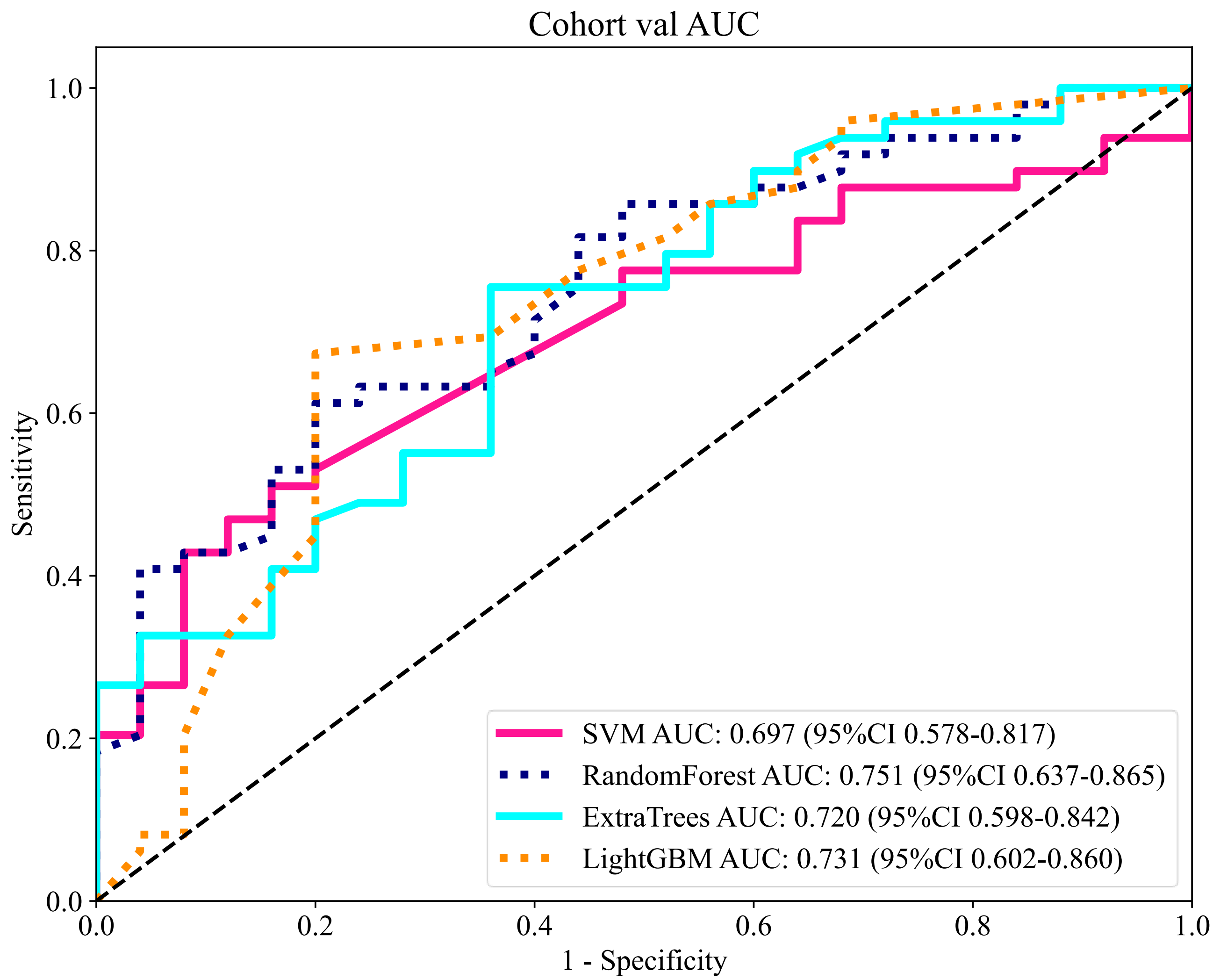

Cohort val AUC
Sensitivity
1 - Specificity
0.0
0.2
0.4
0.6
0.8
1.0
SVM AUC: 0.697 (95%CI 0.578-0.817)
RandomForest AUC: 0.751 (95%CI 0.637-0.865)
ExtraTrees AUC: 0.720 (95%CI 0.598-0.842)
LightGBM AUC: 0.731 (95%CI 0.602-0.860)

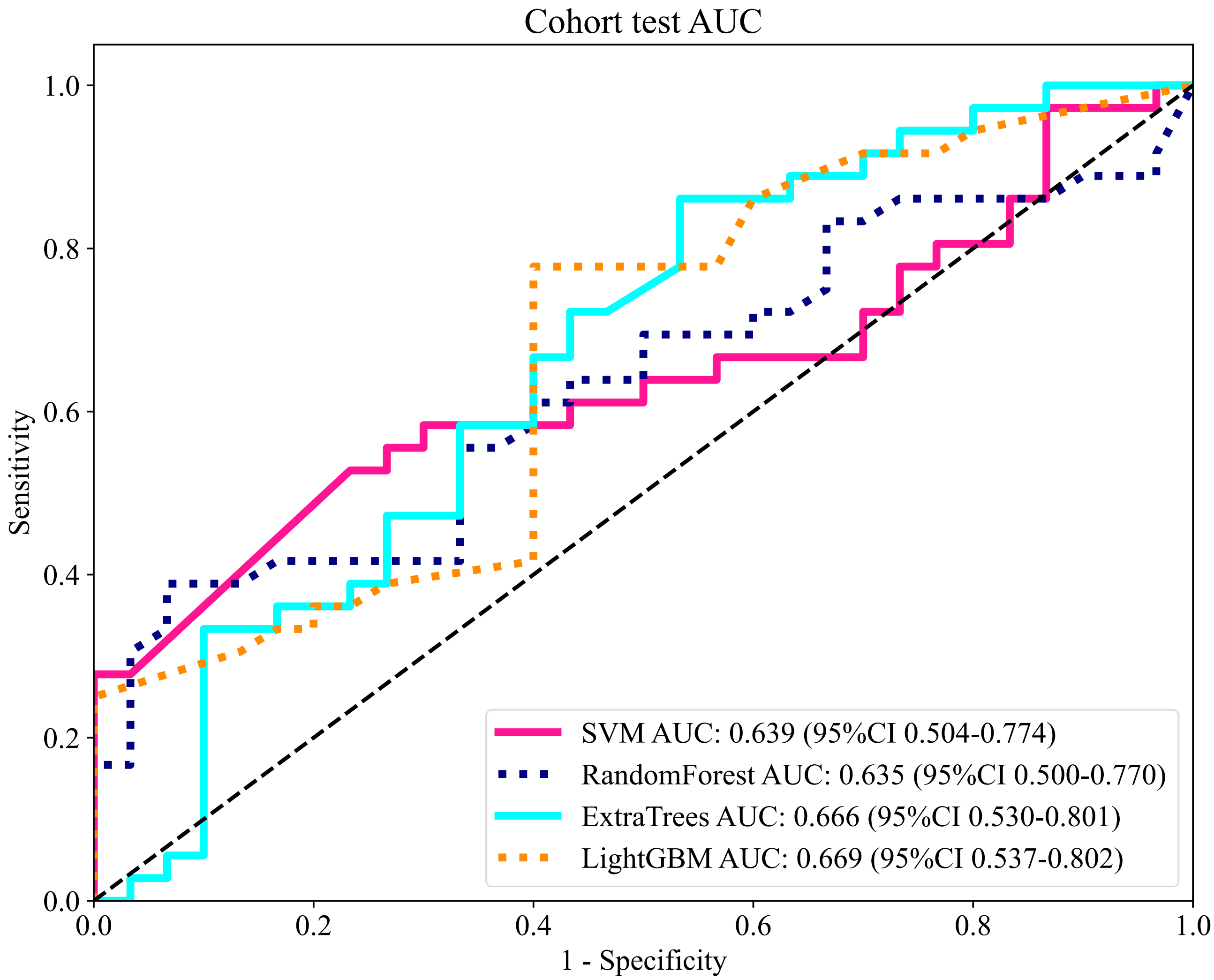

Figure S19. ROC curves of different machine learning models for Peri5mm.

Supplementary Figure (file)

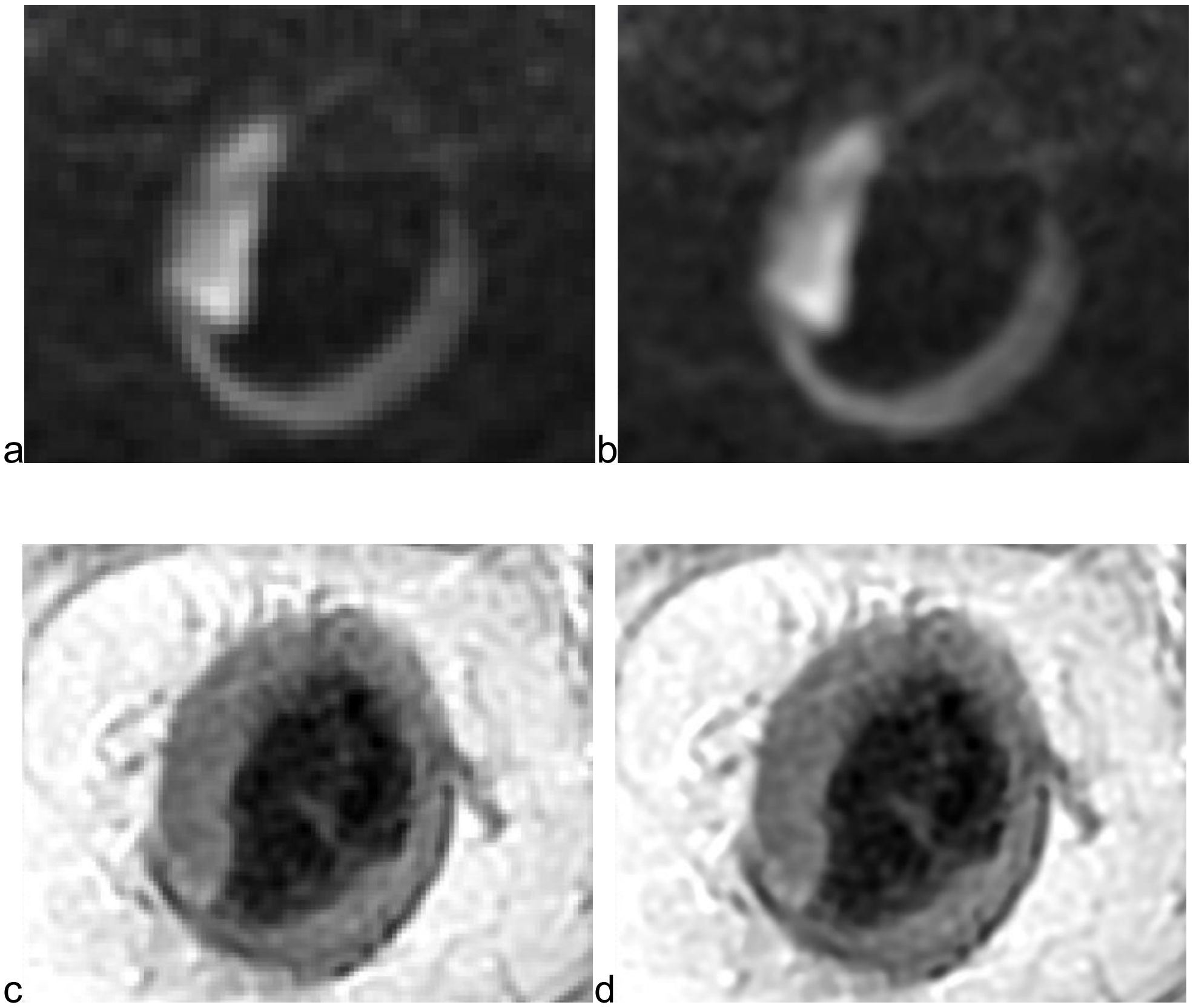

Supplementary Figure 1 Visual comparison of GAN-enhanced super-resolution MRI reconstruction demonstrates structural fidelity improvements between conventional acquisitions and processed outputs: **(a)** high-resolution diffusion-weighted imaging (HR-DWI) versus **(b)** super-resolution DWI (SR-DWI); **(c)** high-resolution T2-weighted imaging (HR-T2WI) **(d)** super-resolution T2WI (SR-T2WI). The SR-reconstructed sequences exhibit enhanced capacity for structural demarcation, particularly in peripheral tissue boundaries and neural-vascular interfaces, whereas conventional high-resolution modalities fail to resolve these

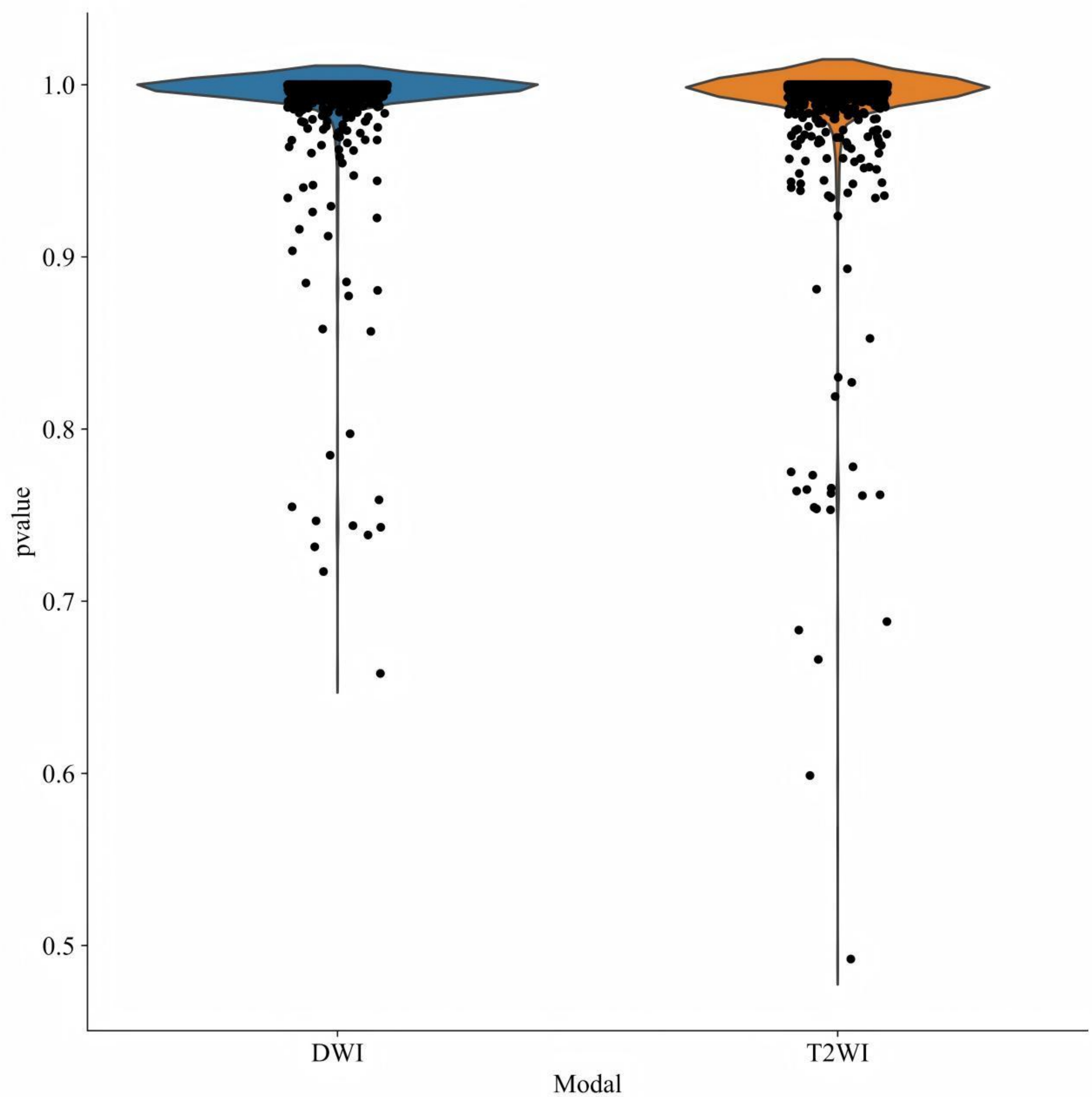

microarchitectural details

Supplementary Figure 2. Distribution of Intraclass Correlation Coefficients (ICC) for extracted radiomic features. The plot displays the density and range of ICC values for DWI features (blue) and T2WI features (orange). The median ICC for both sequences is approximately 0.999, confirming high reproducibility.

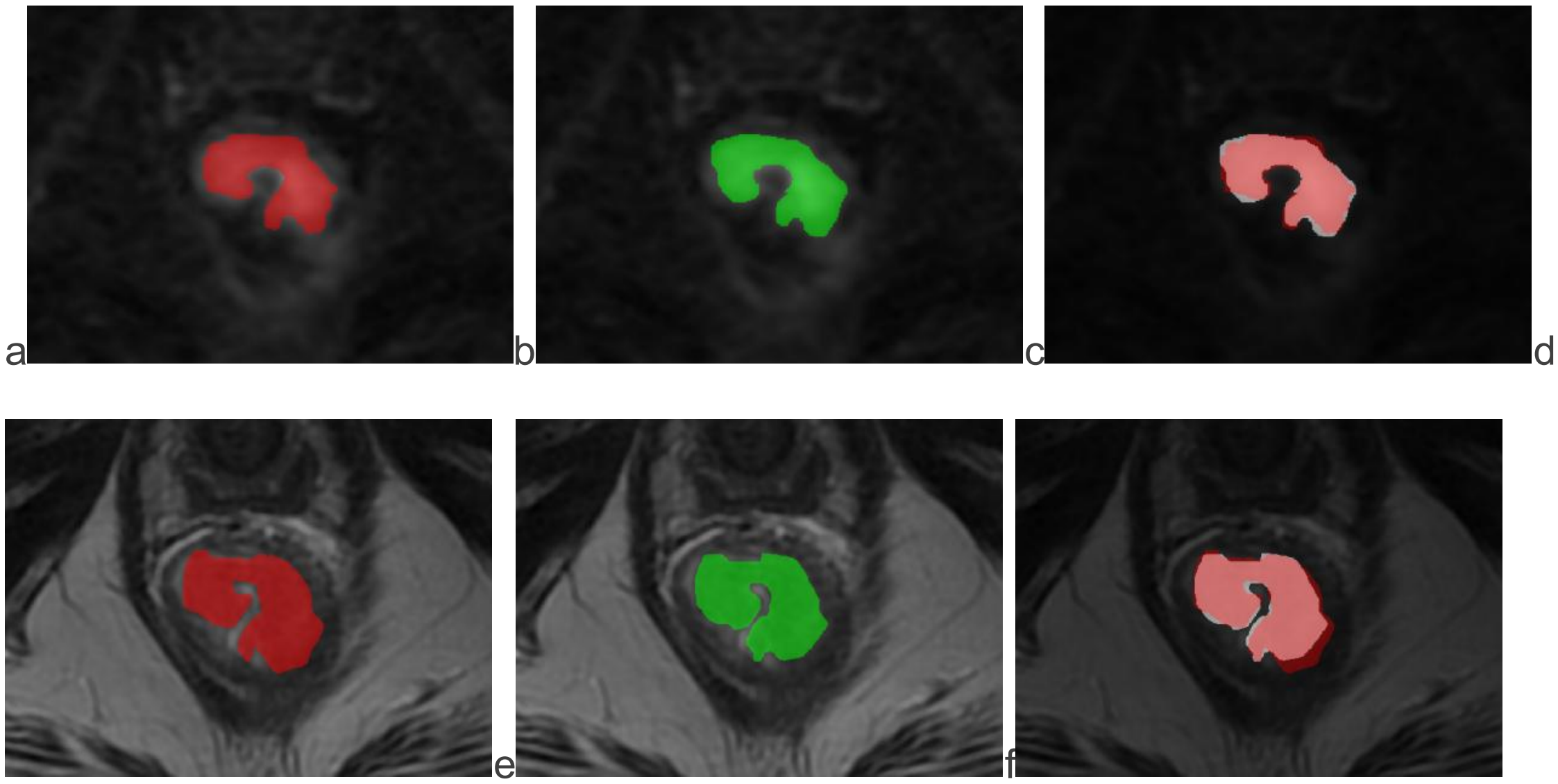

Supplementary Figure 3. Visual assessment of inter-reader segmentation agreement. Representative axial images illustrating the reproducibility of Region of Interest (ROI) delineation on super-resolution reconstructed images. (a–c) Diffusion-Weighted Imaging (DWI): (a) ROI delineated by Reader 1 (red contour); (b) ROI delineated by Reader 2 (green contour); (c) Overlay of both ROIs showing substantial spatial overlap and consistency. (d–f) T2-Weighted Imaging (T2WI): (d) ROI delineated by Reader 1 (red contour); (e) ROI delineated by Reader 2 (green contour); (f) Overlay of both ROIs demonstrating high agreement in tumor boundary definition.

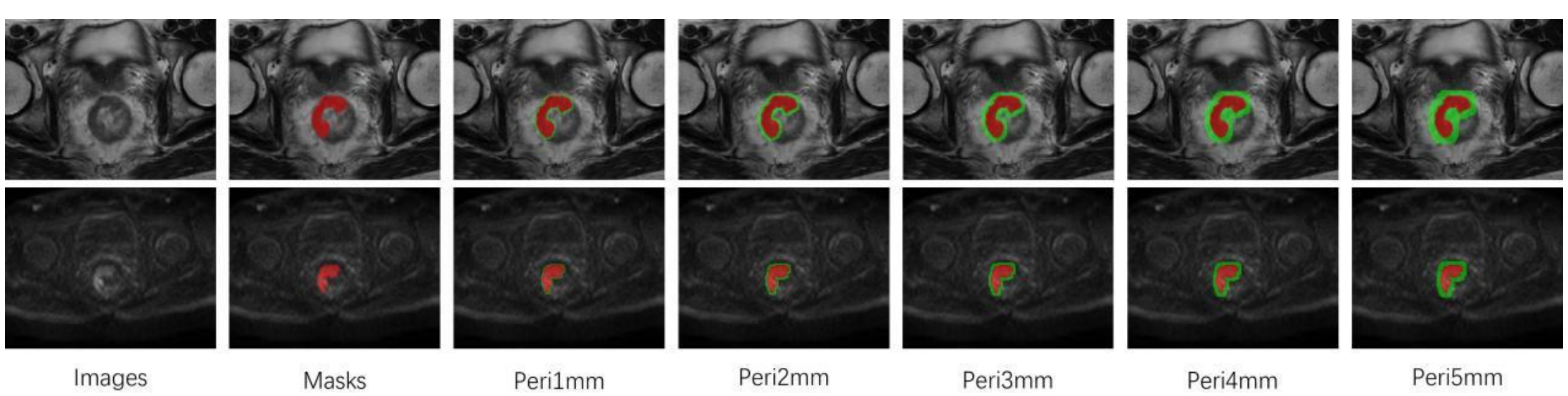

Supplementary Figure 4 Peritumoral expansion from 1 mm to 5 mm

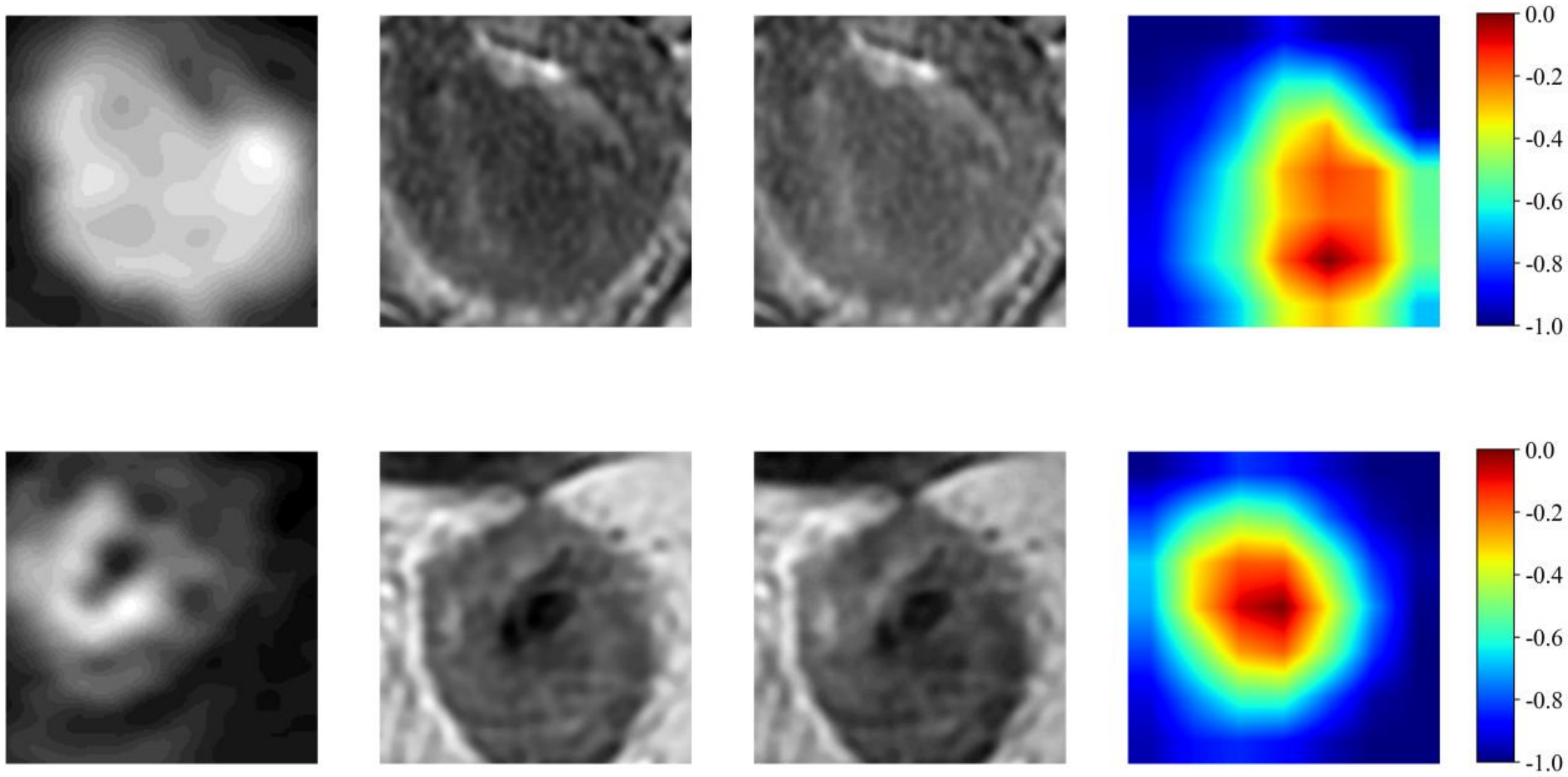

Supplementary Figure 5 Exemplary cases showcased the attention patterns specific to each modality, including DWI, T2WI, and Mixup-synthesized images. The spatial activation heatmaps exposed distinct foci on the perineural microenvironments and tumor margins. This not only verified the model's decision-making rationale but also its clinical interpretability.

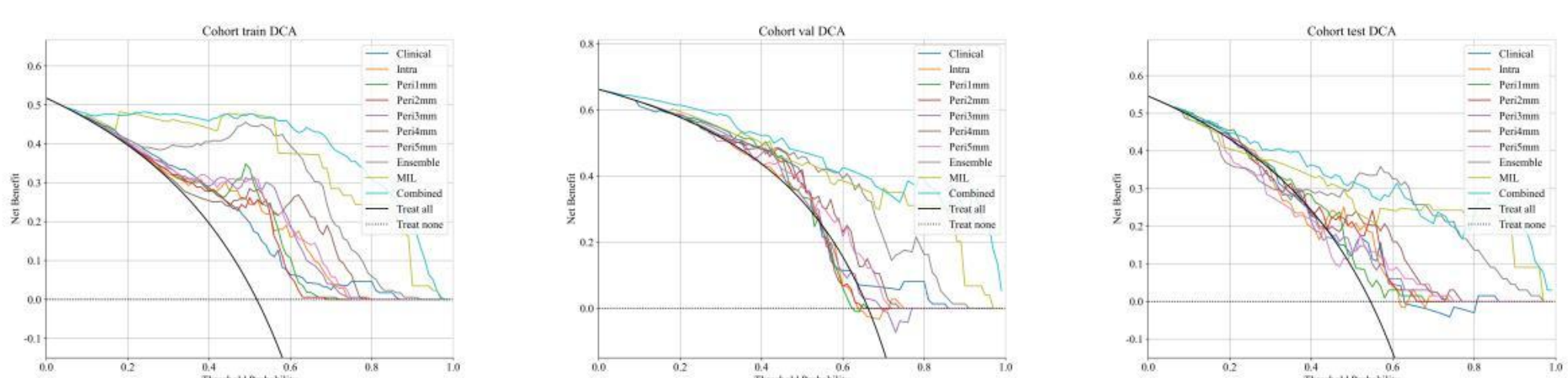

a b c

Supplementary Figure 6 The Decision curve analysis(DCA) of the all models in the training(a), internal validation(b), and external testing cohorts(c).

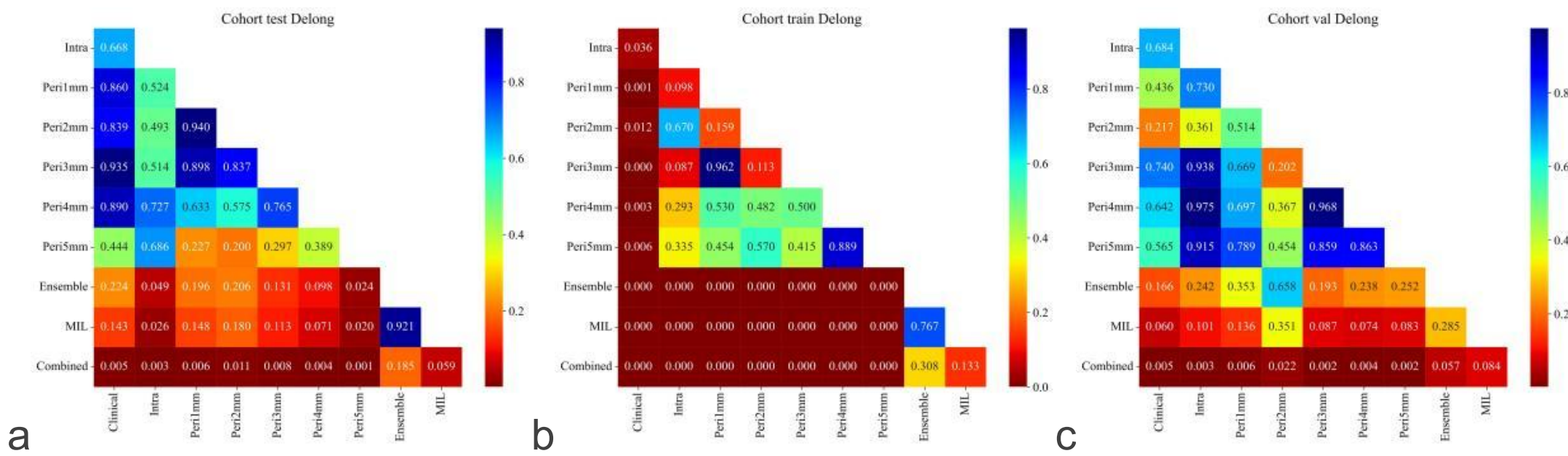

Supplementary Figure 7 Heatmaps displaying the P-values from DeLong tests comparing the AUCs of different models across cohorts. Red to blue colors indicate the P-value magnitude. In the Validation Cohort, the Combined Model showed significant differences compared to the Clinical (p=0.005), Intra (p=0.003), and Peri2mm (p=0.022) models. In the External Testing Cohort, the Combined Model also significantly outperformed the Clinical (p=0.005), Intra (p=0.003), and Peri2mm (p=0.011) models.

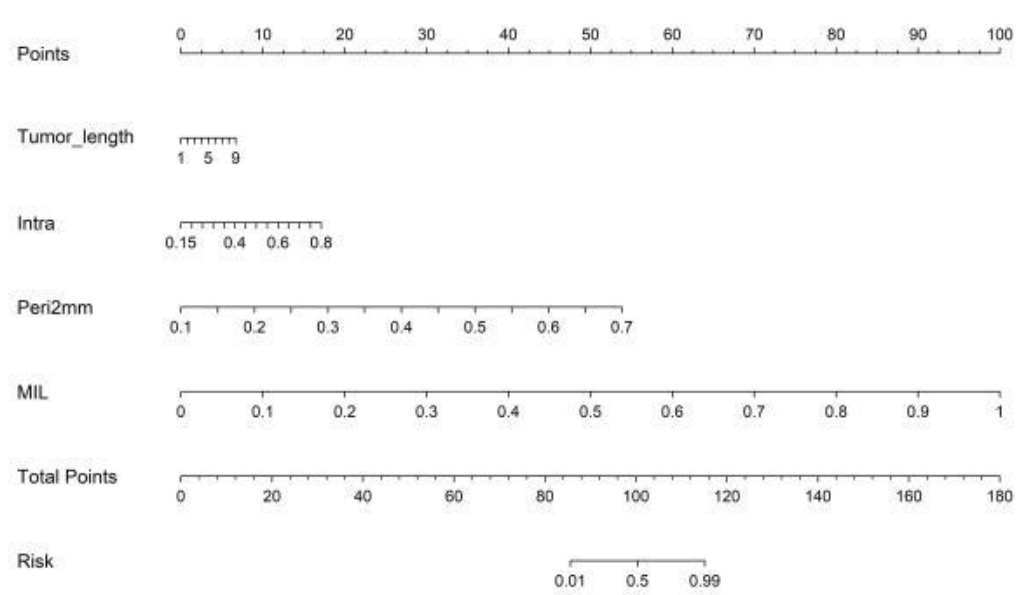

Supplementary Figure 8 Final nomogram incorporating tumor length, intra, peri2mm, and MIL for predicting PNI status.

Abbreviations: AUC: area under the curve; CI: confidence interval; Intra: intratumoral; PeriXmm: “ X ” denotes the peritumoral region; MIL:multi-instance learning
