# Supplementary material for: Super-resolution MRI and 2.5D deep learning for intratumoral-peritumoral radiomics in preoperative prediction of rectal cancer perineural invasion
Source: Insights Imaging. 2026 Aug 22;17:205. doi: 10.1186/s13244-026-02365-7 (PMC13499786; doi:10.1186/s13244-026-02365-7)
Supplement: Supplementary file 2 [file 13244_2026_2365_MOESM2_ESM.pdf]

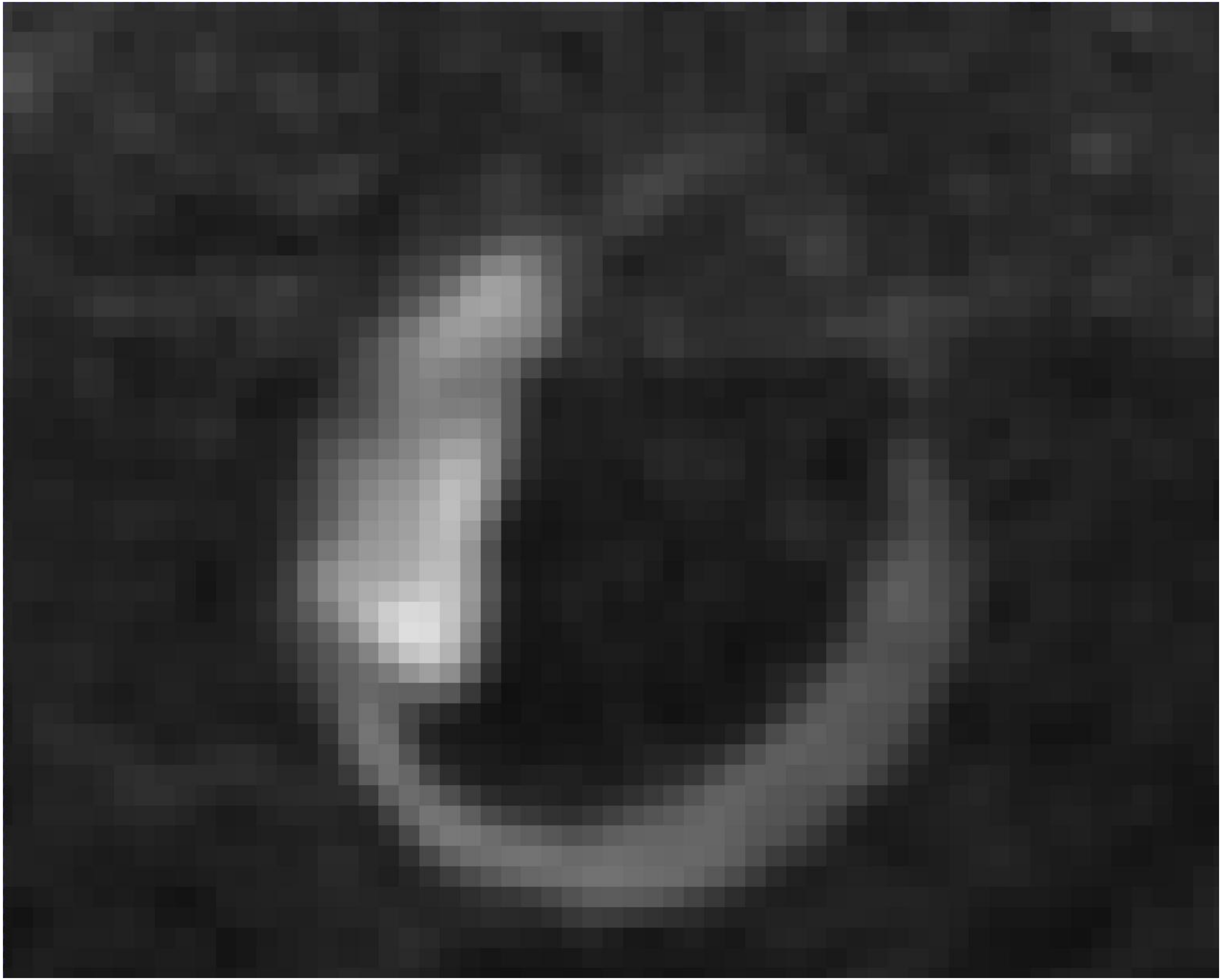

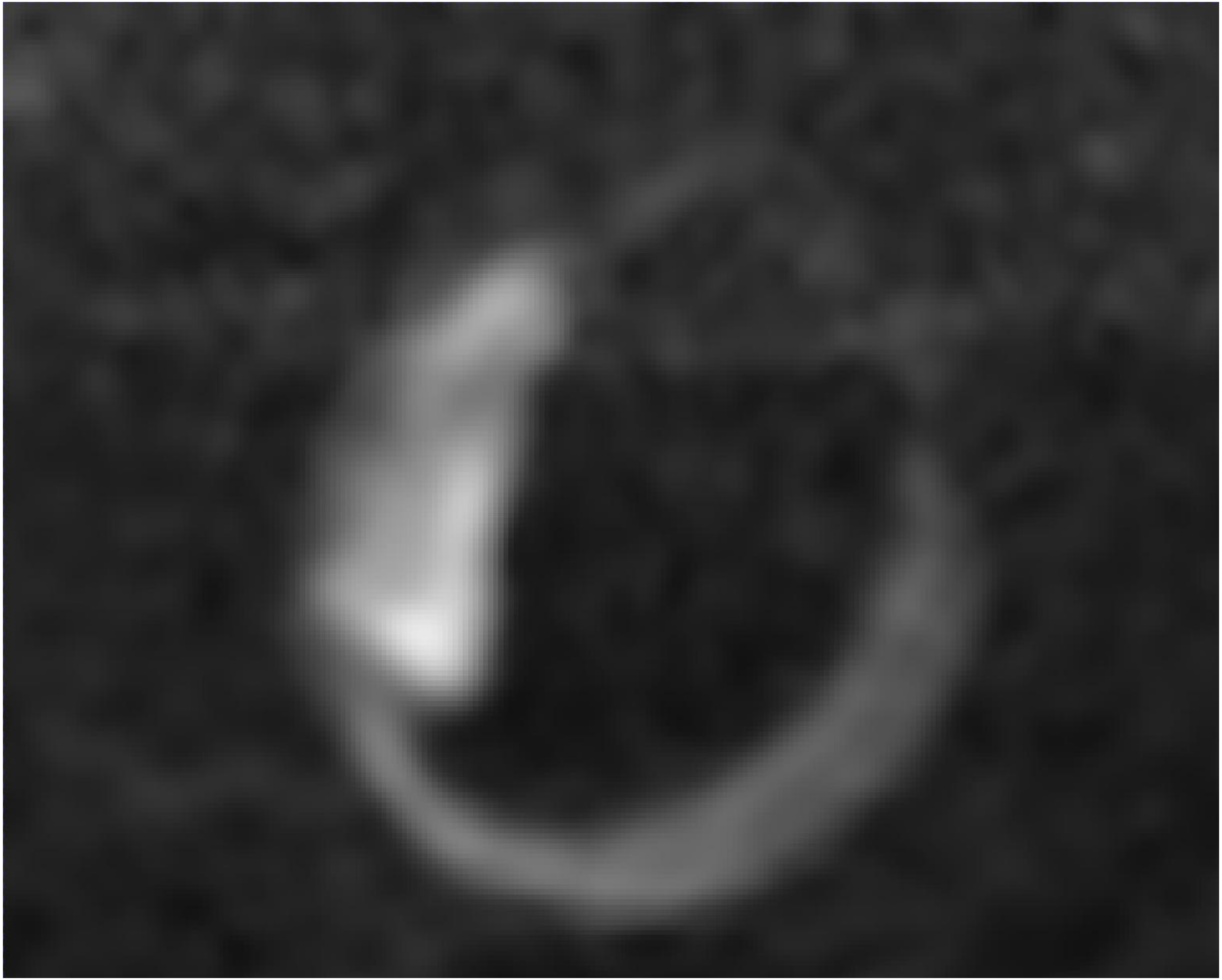

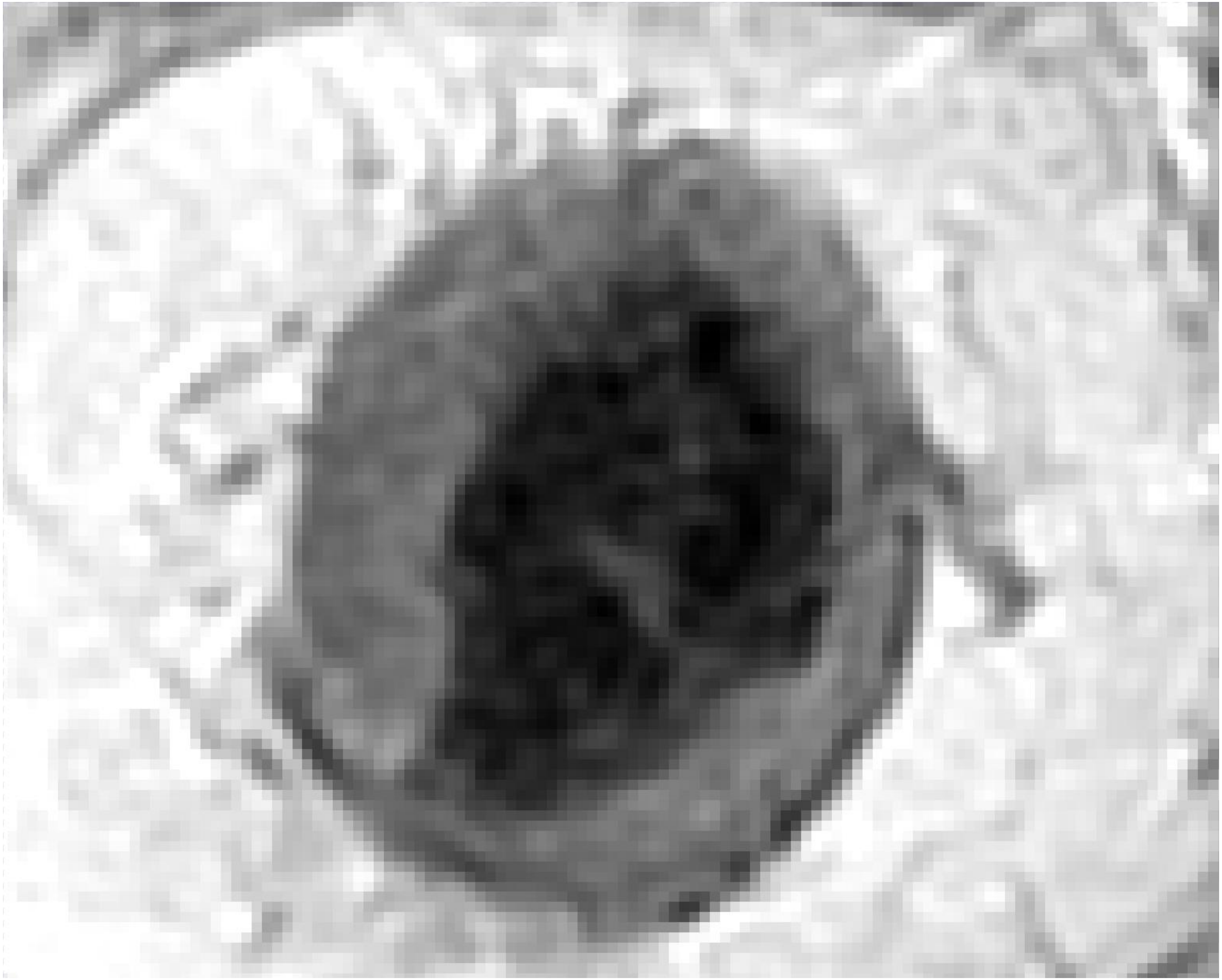

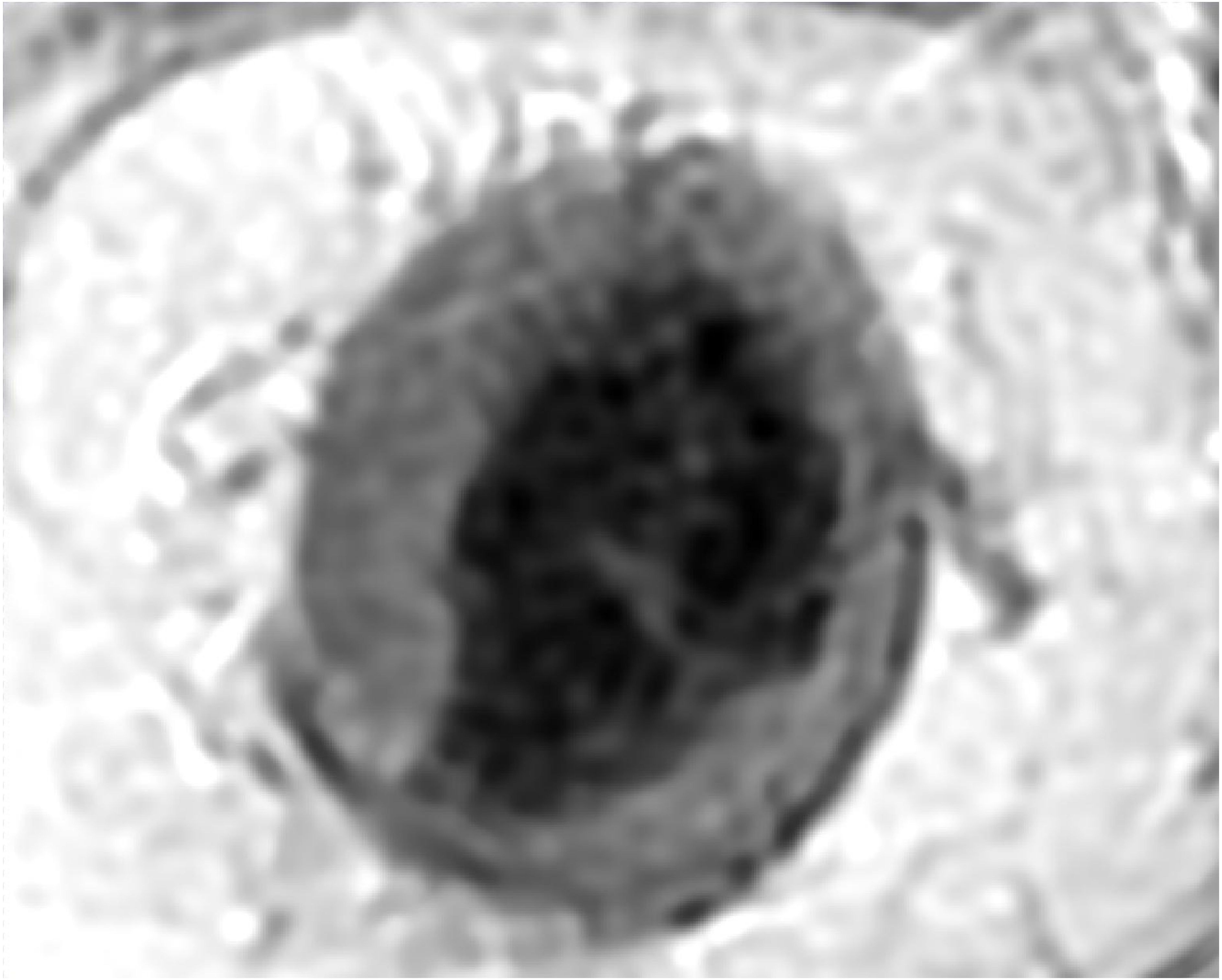

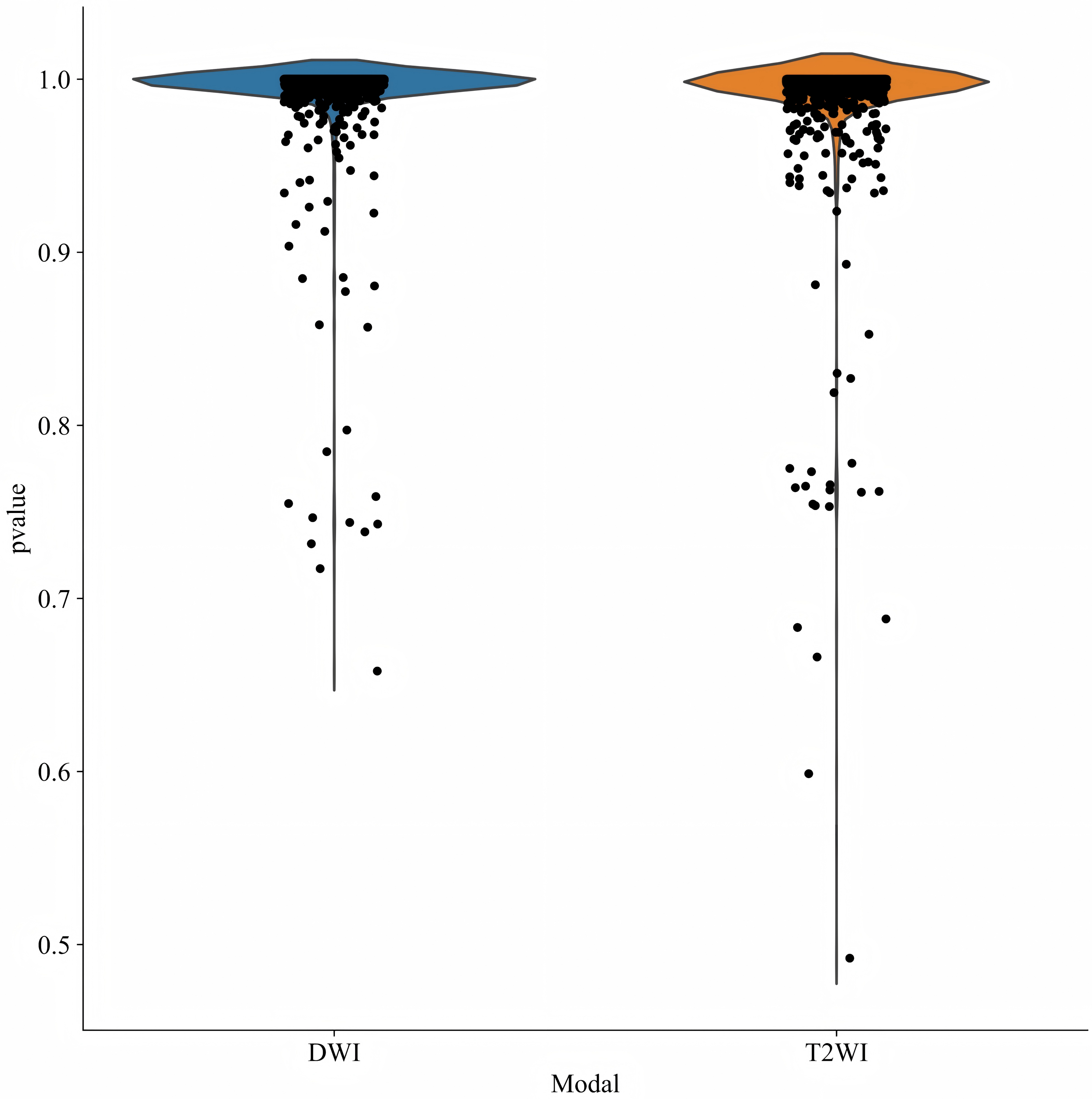

pvalue
1.0
0.9
0.8
0.7
0.6
0.5
DWI
T2WI
Modal

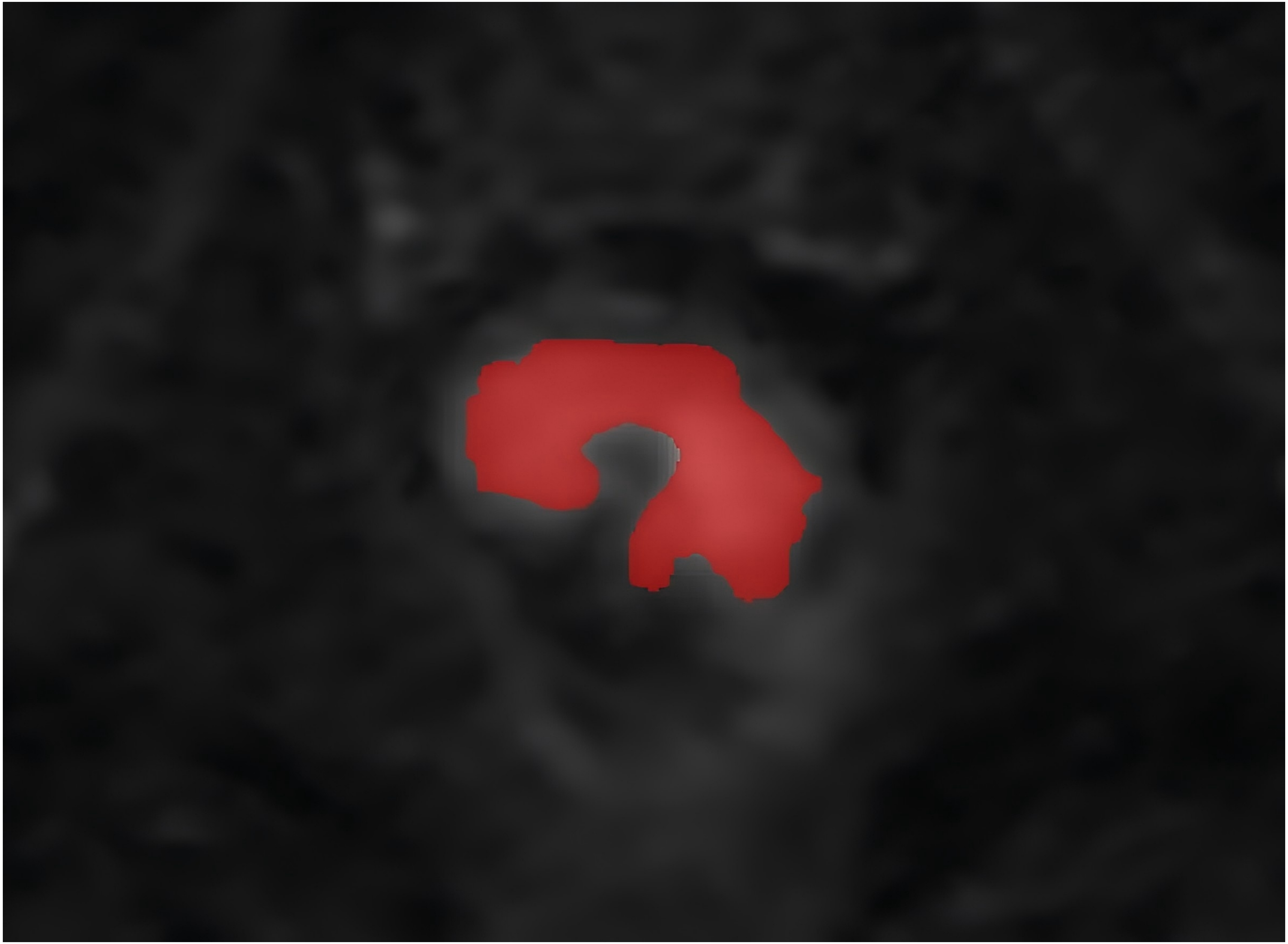

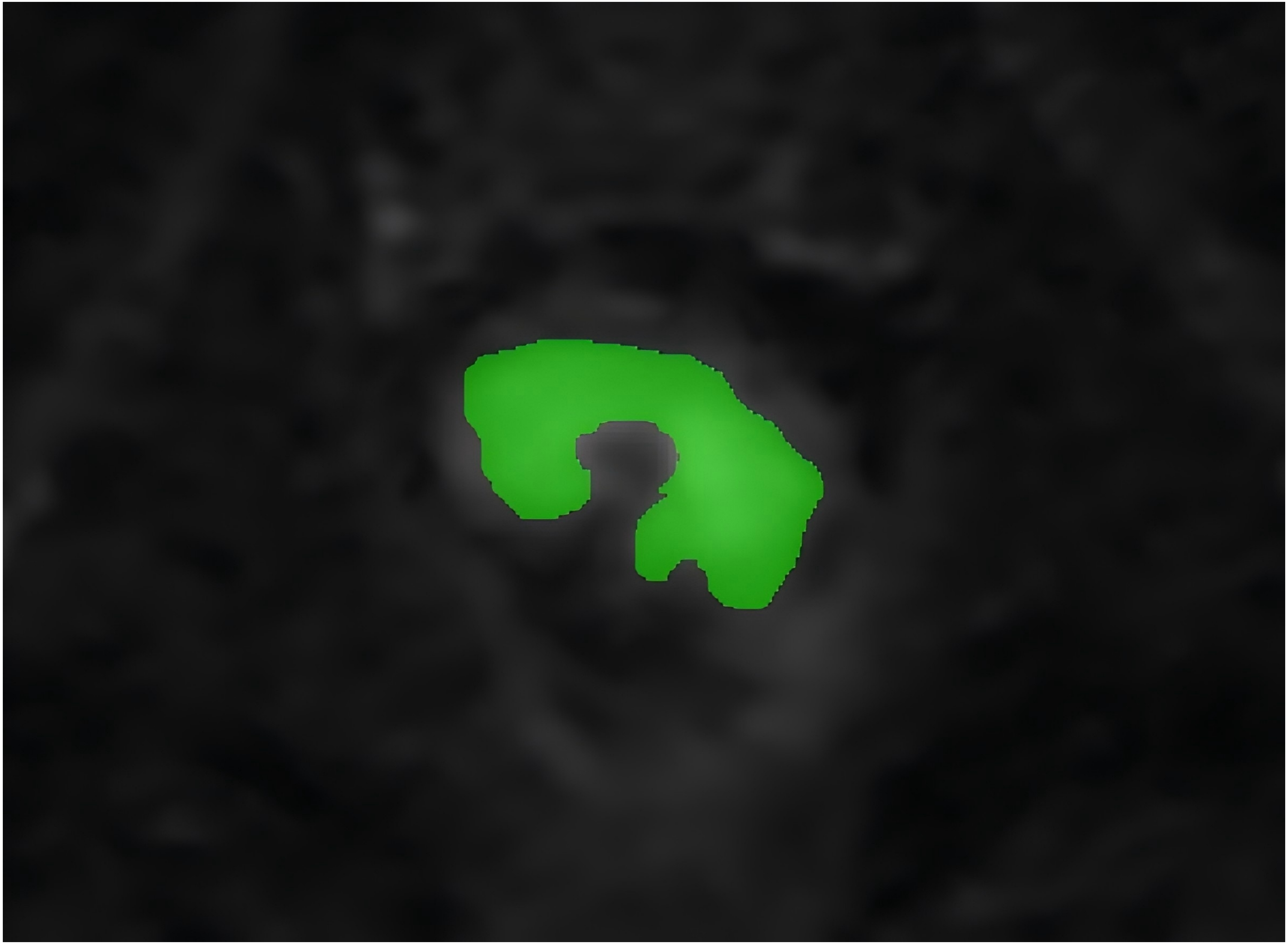

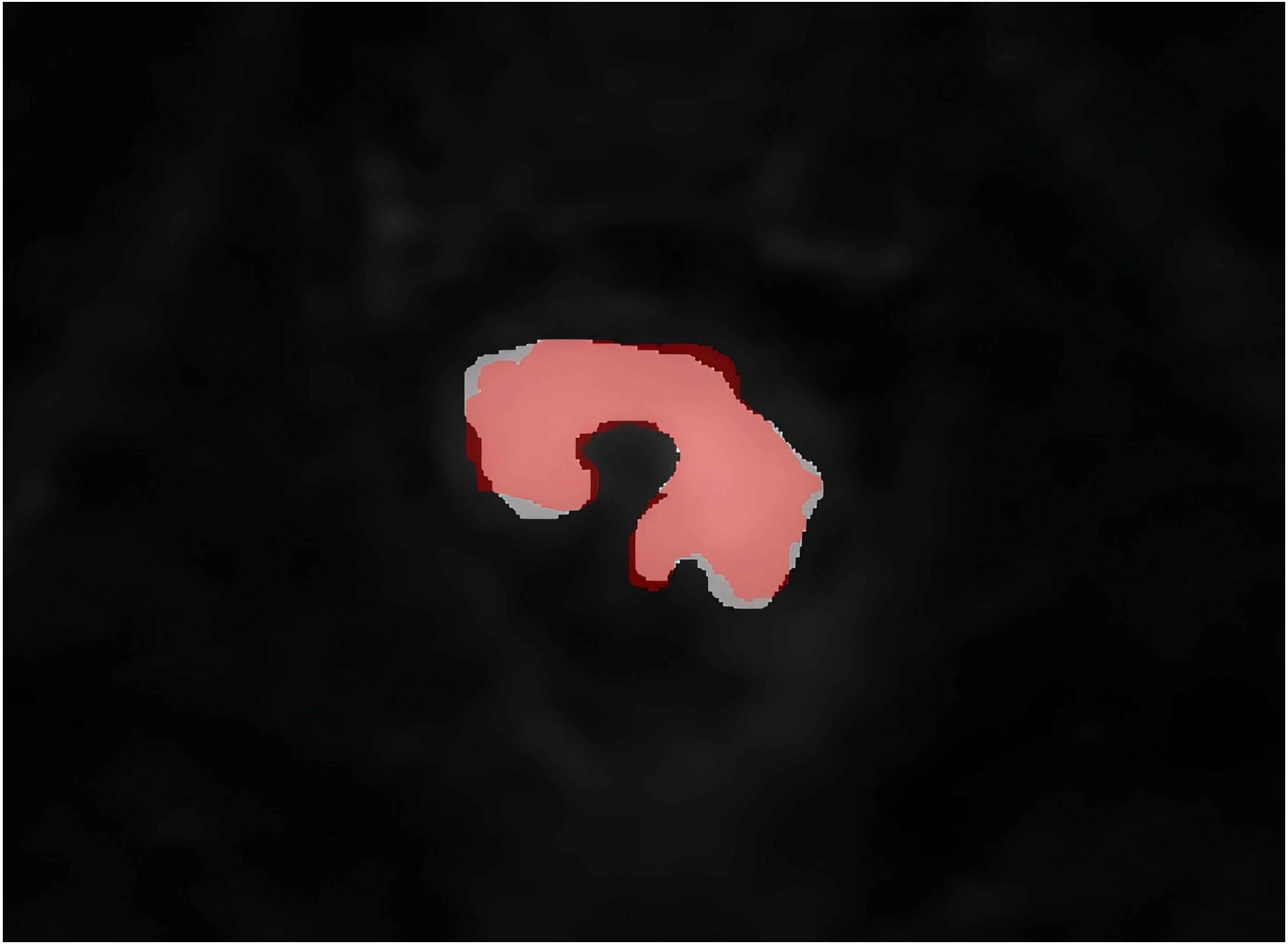

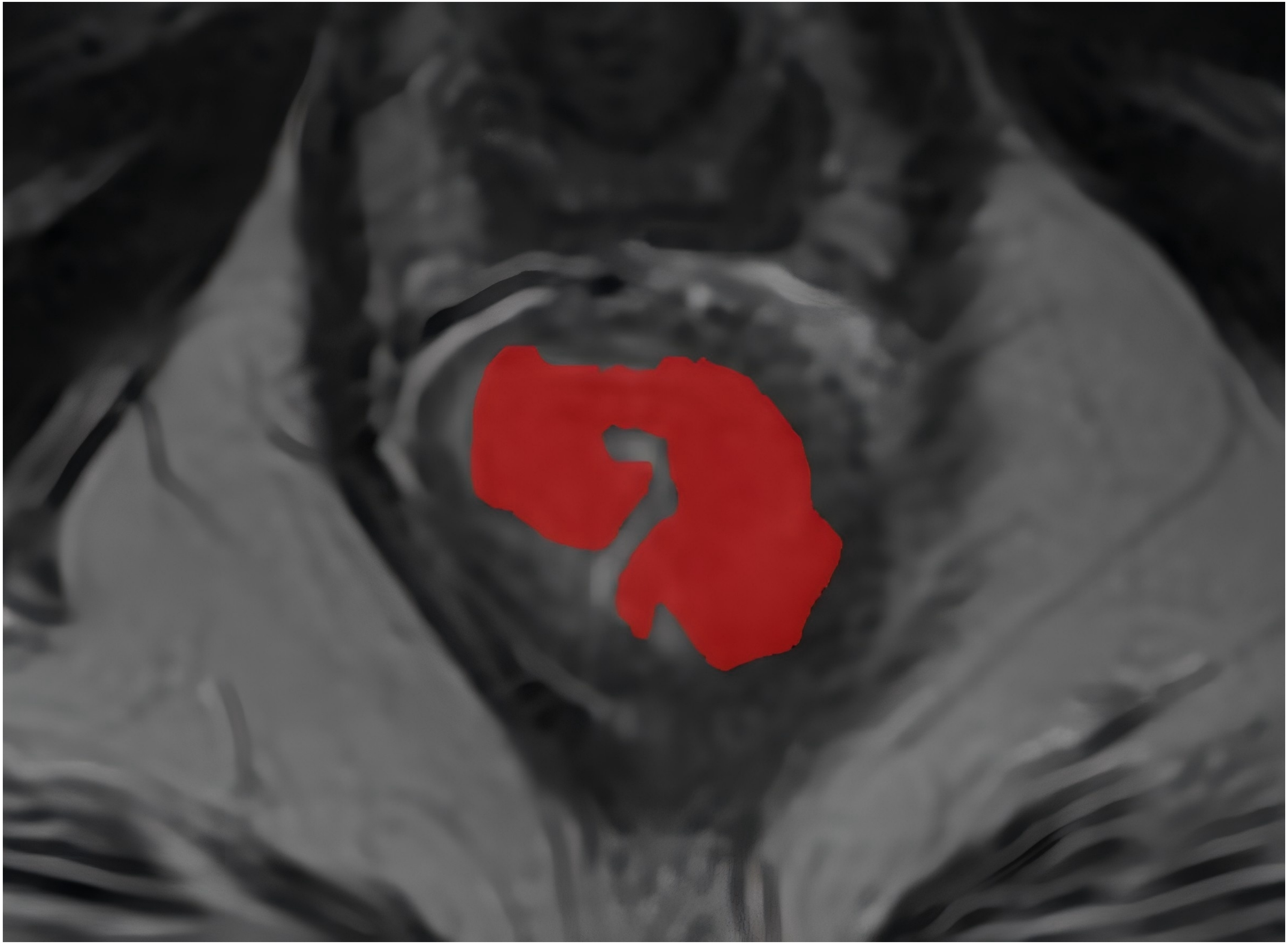

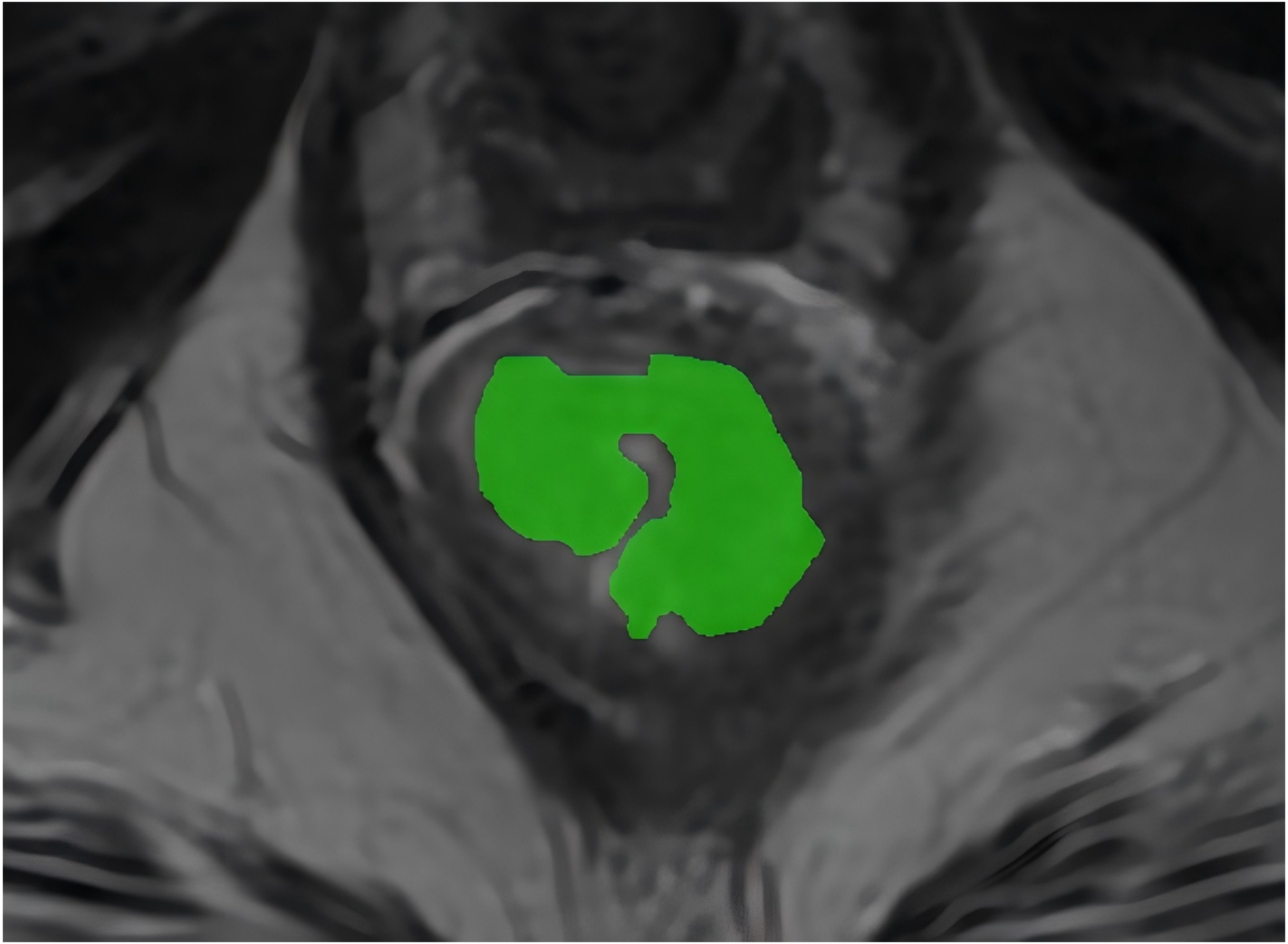

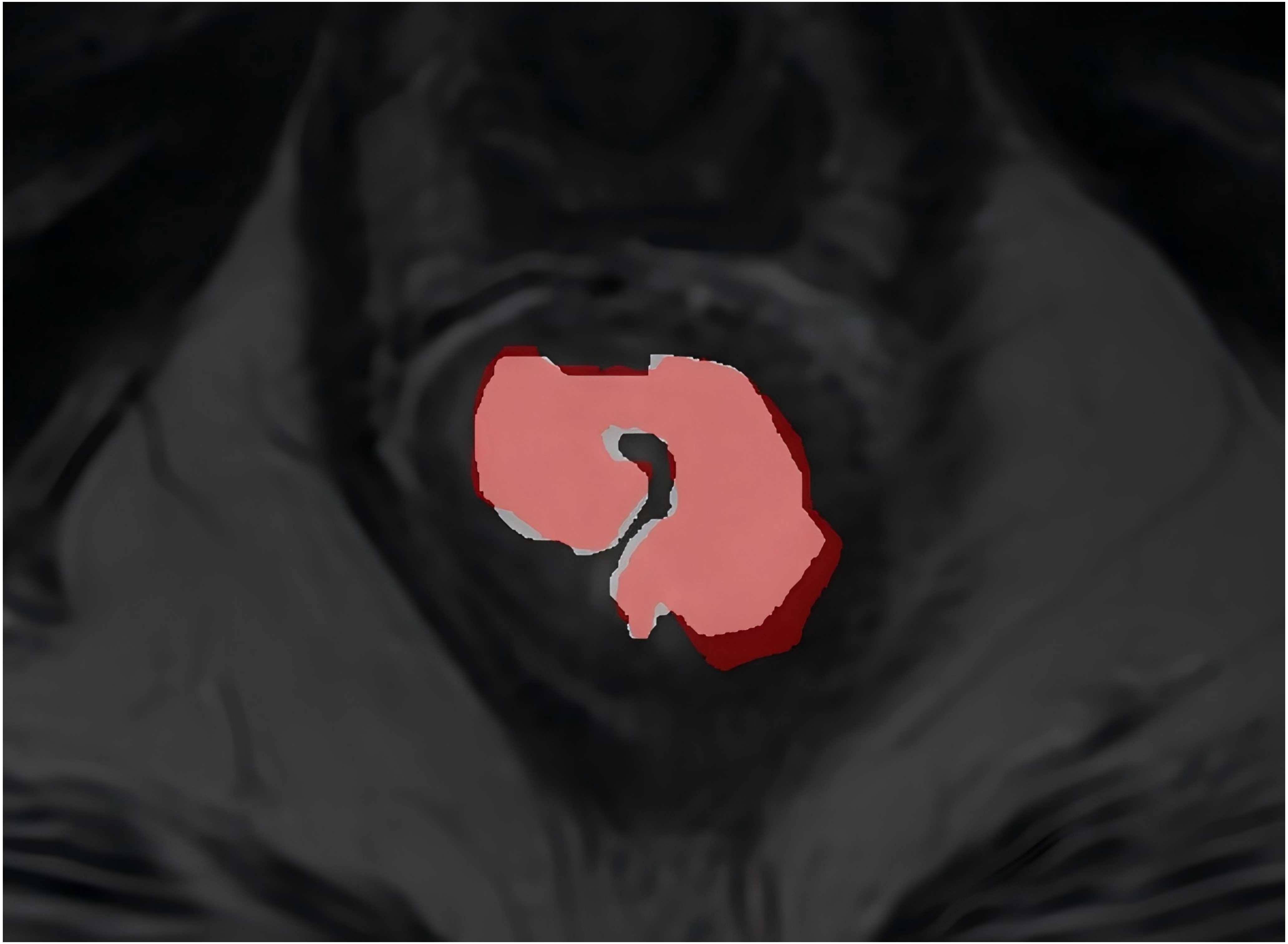

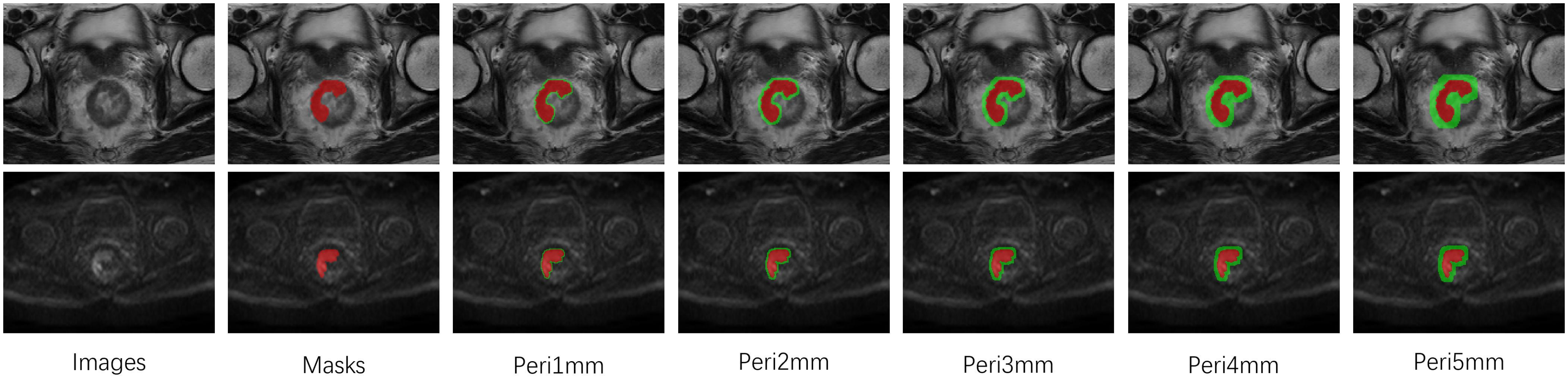

Images
Masks
Peri1mm
Peri2mm
Peri3mm
Peri4mm
Peri5mm

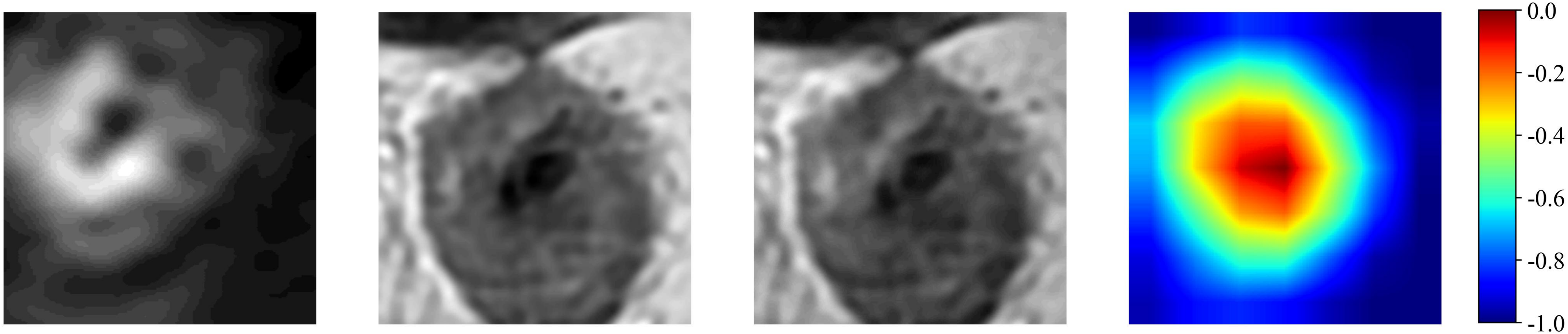
0.0
-0.2
-0.4
-0.6
-0.8
-1.0

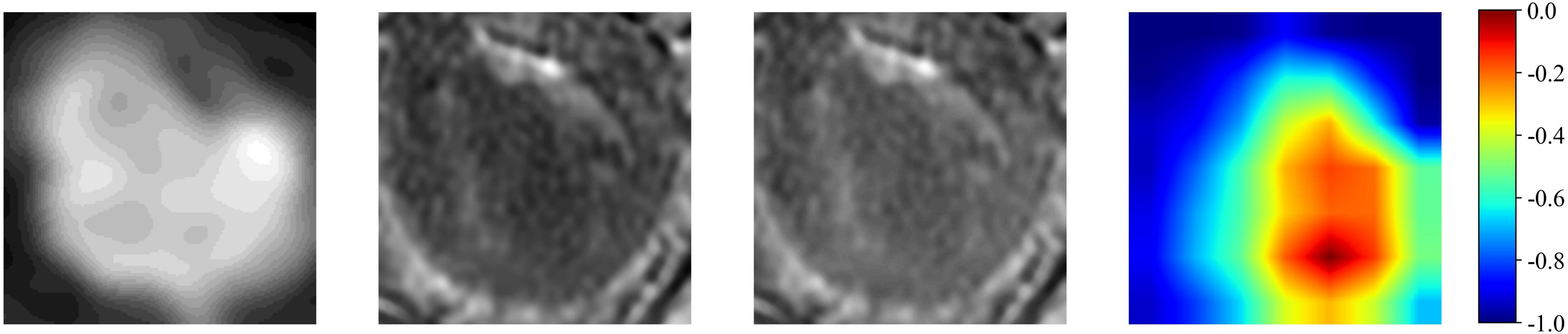
0.0
-0.2
-0.4
-0.6
-0.8
-1.0

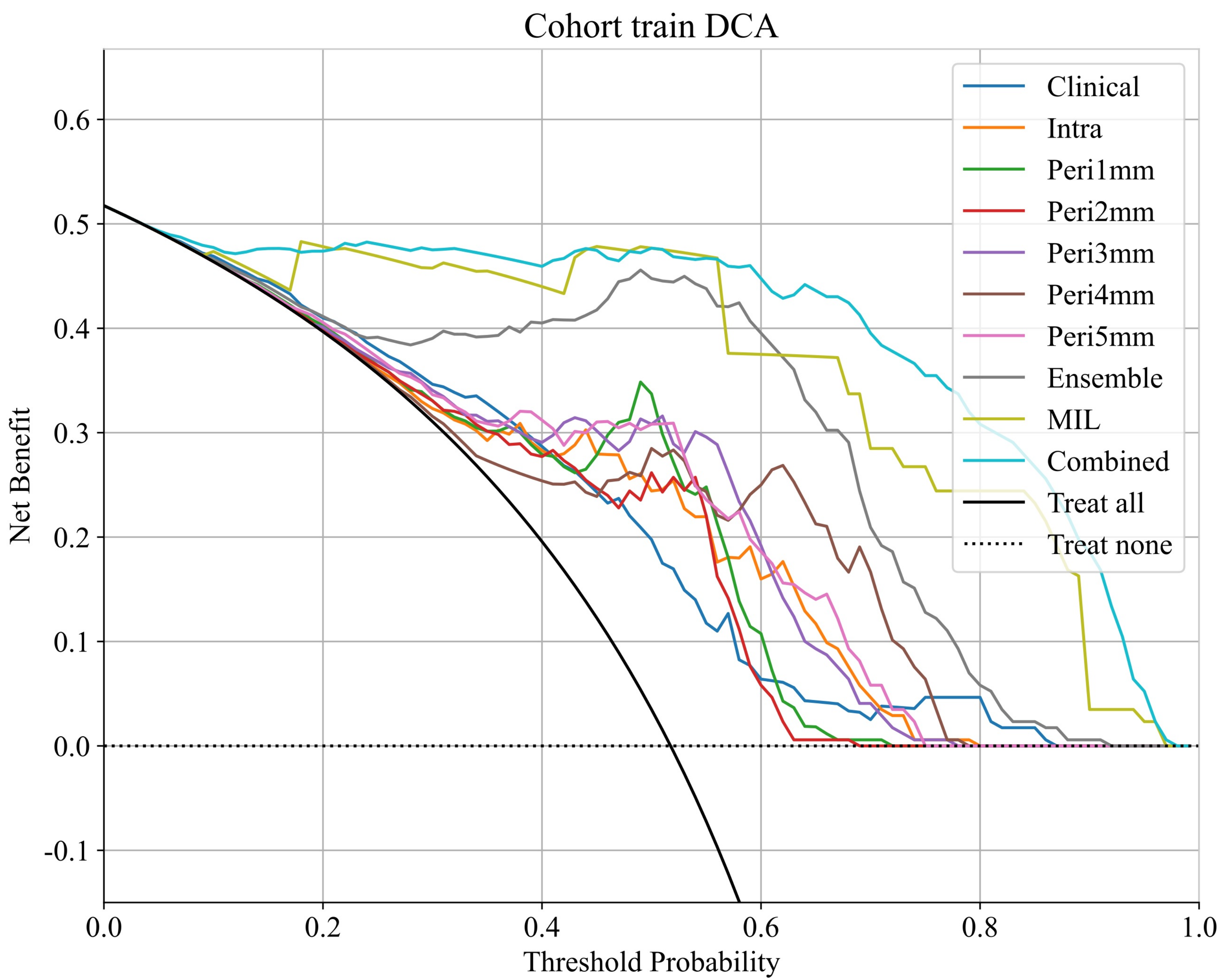

Cohort train DCA
Clinical
Intra
Peri1mm
Peri2mm
Peri3mm
Peri4mm
Peri5mm
Ensemble
MIL
Combined
Treat all
Treat none
Net Benefit
Threshold Probability
0.6
0.5
0.4
0.3
0.2
0.1
0.0
-0.1
0.0
0.2
0.4
0.6
0.8
1.0

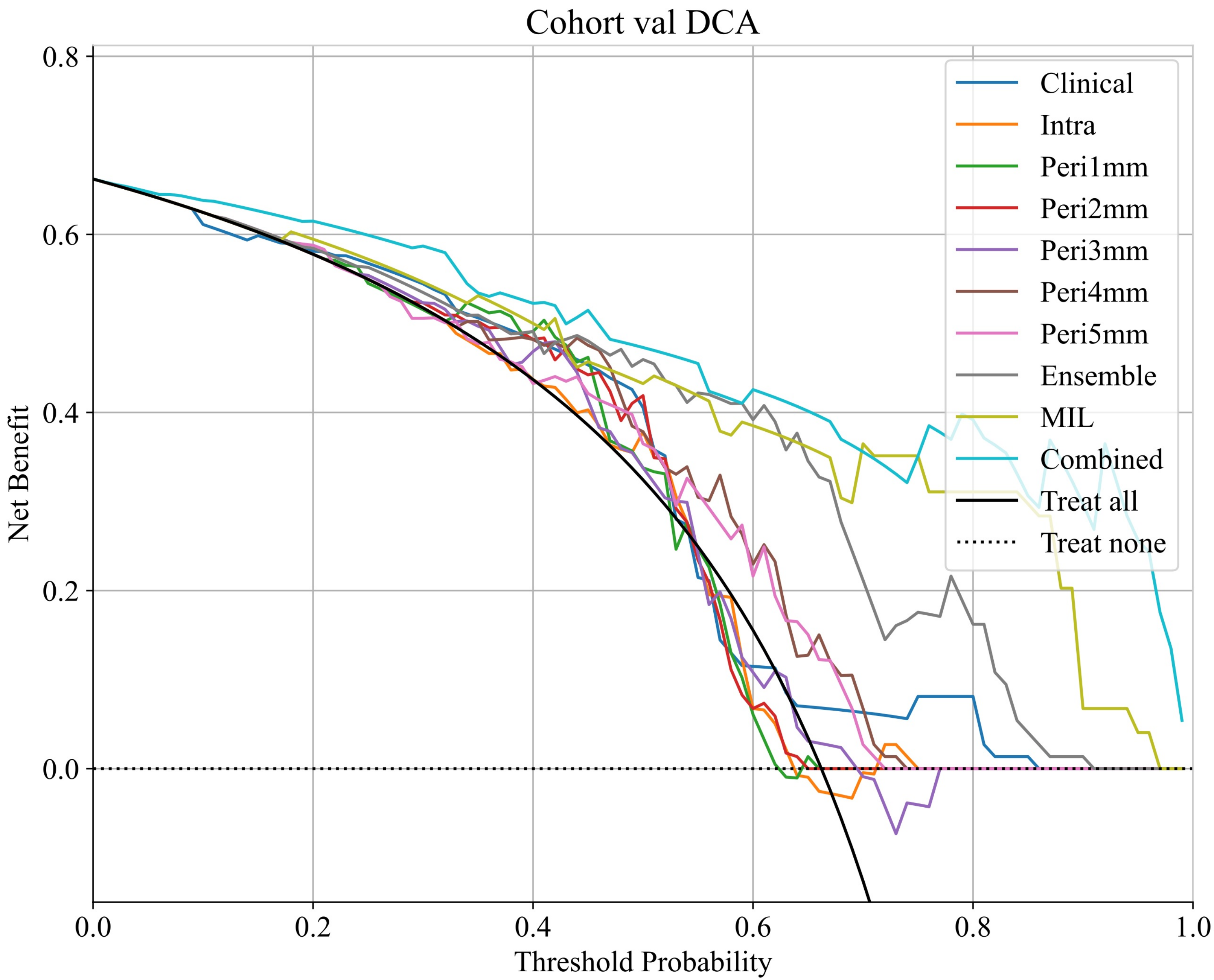

Cohort val DCA
Net Benefit
Threshold Probability
0.8
0.6
0.4
0.2
0.0
0.0
0.2
0.4
0.6
0.8
1.0
Clinical
Intra
Peri1mm
Peri2mm
Peri3mm
Peri4mm
Peri5mm
Ensemble
MIL
Combined
Treat all
Treat none

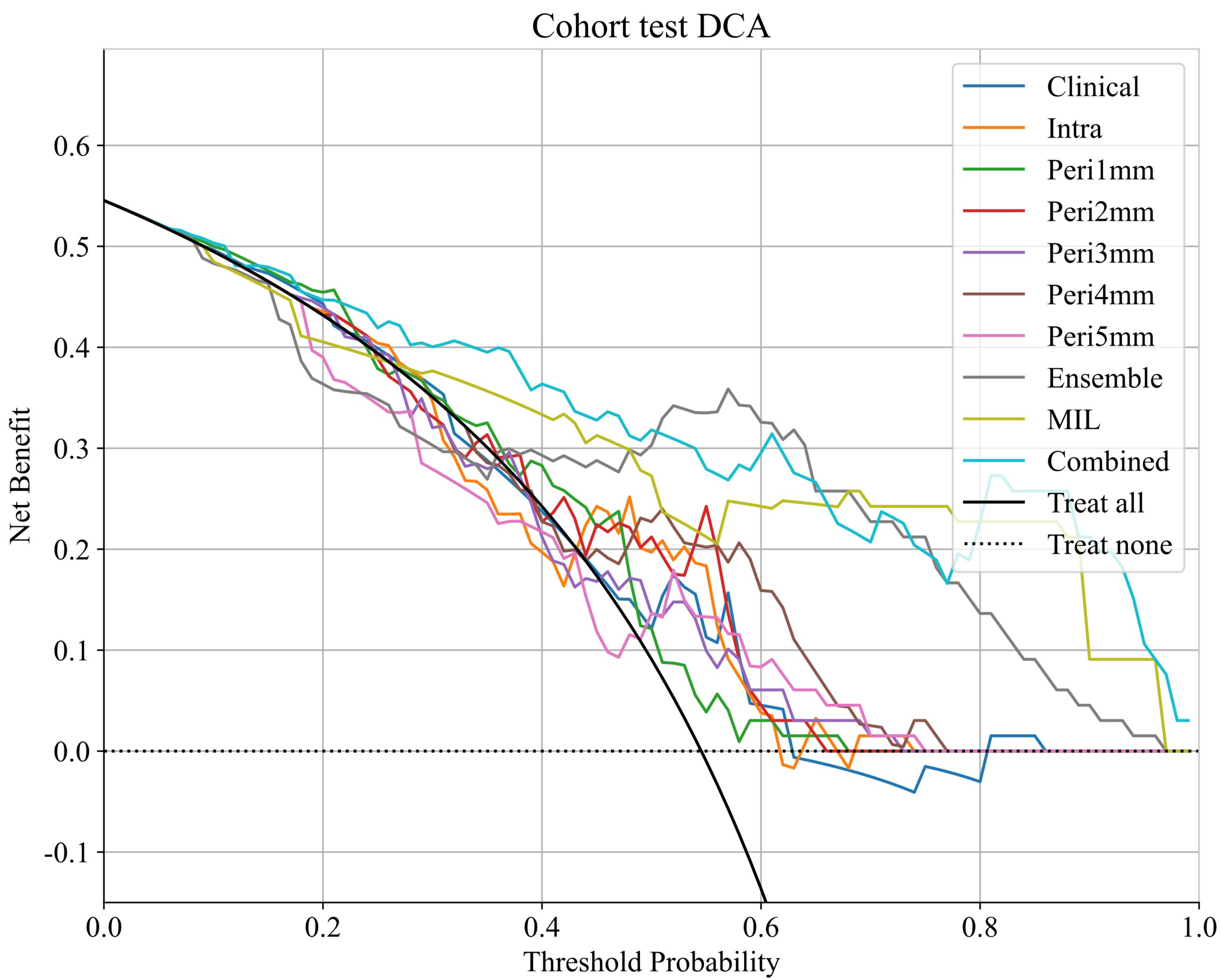
Cohort test DCA
Clinical
Intra
Peri1mm
Peri2mm
Peri3mm
Peri4mm
Peri5mm
Ensemble
MIL
Combined
Treat all
Treat none
Net Benefit
Threshold Probability
0.6
0.5
0.4
0.3
0.2
0.1
0.0
-0.1
0.0
0.2
0.4
0.6
0.8
1.0

Cohort train Delong

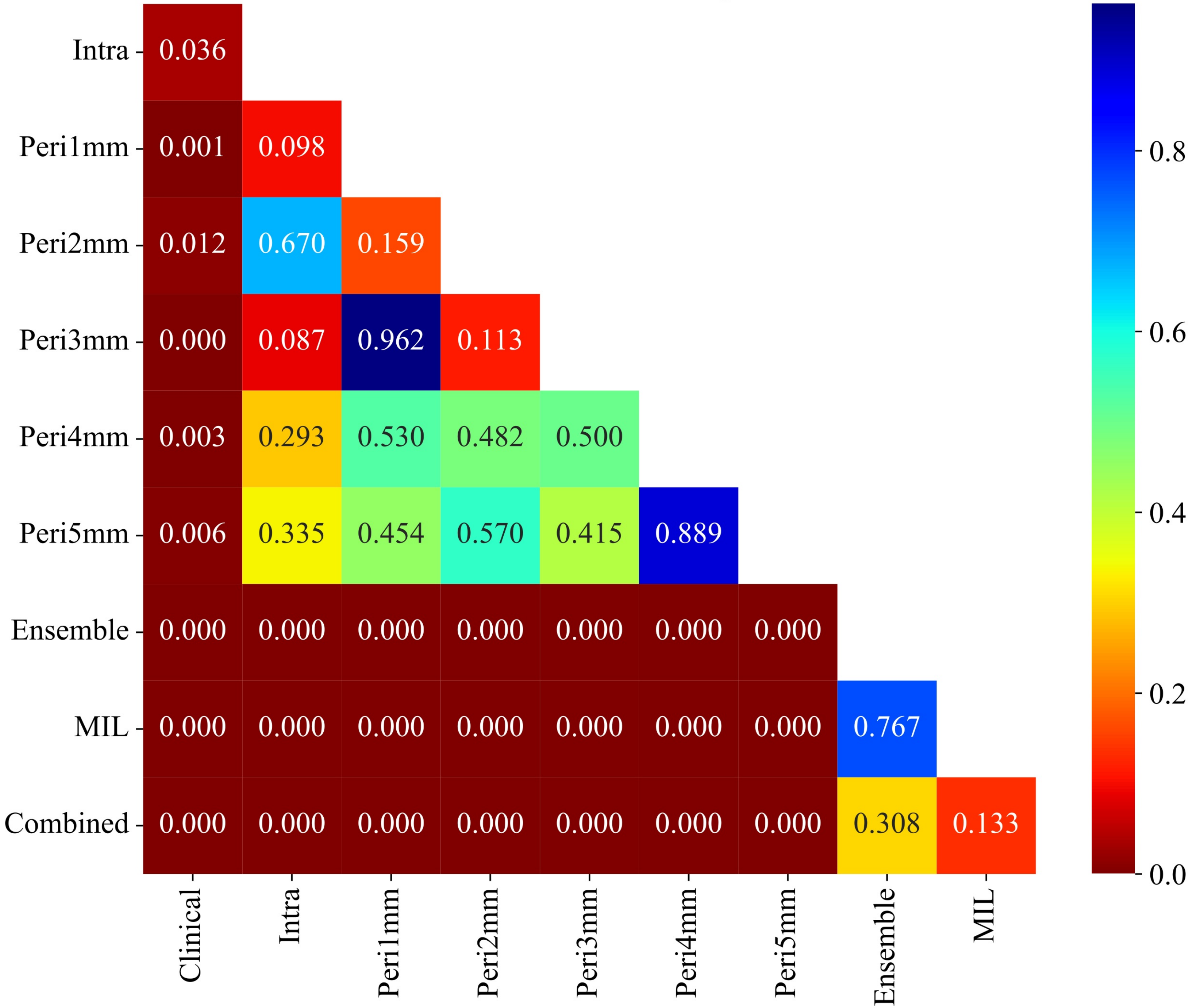

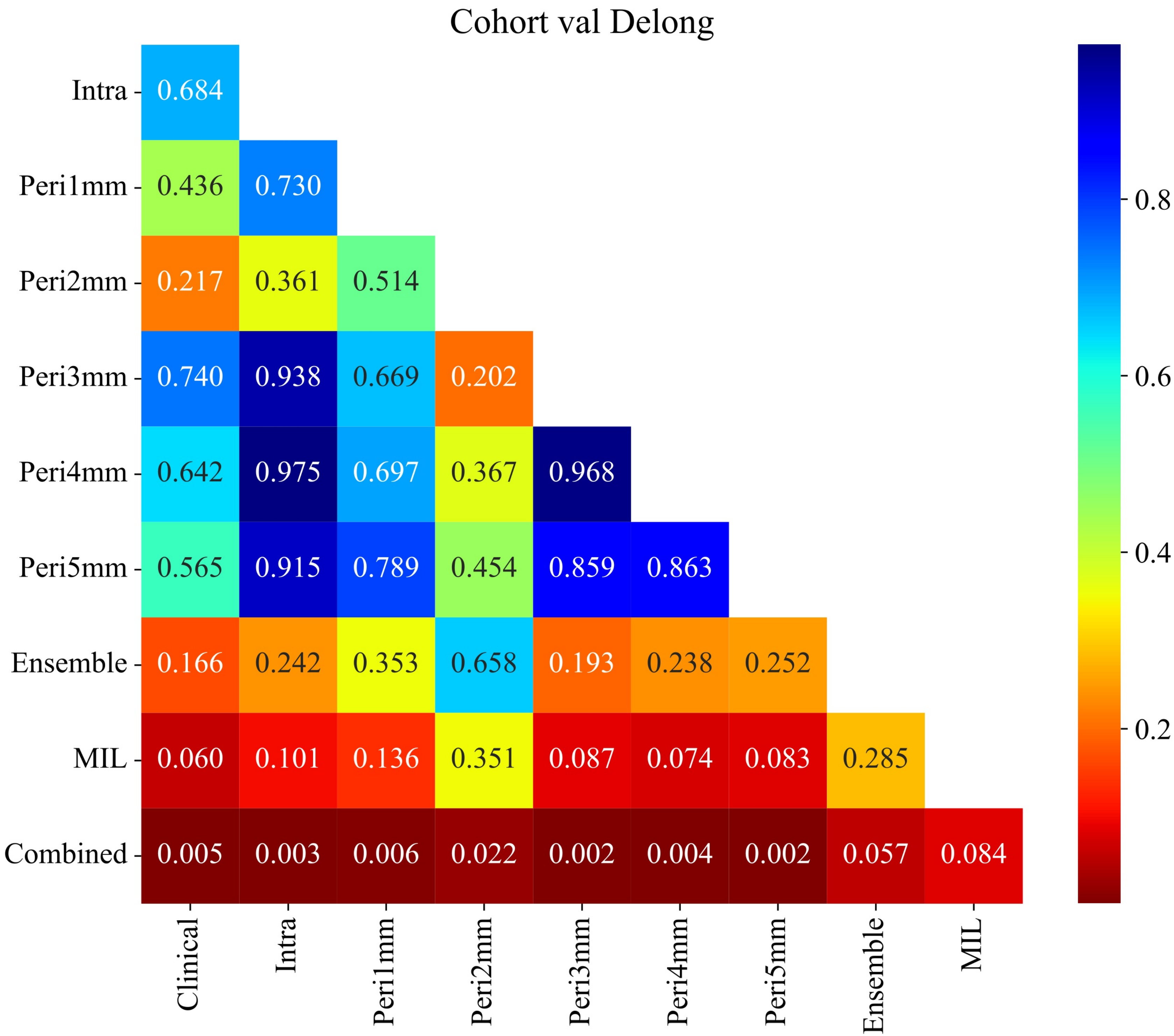

Cohort val Delong
Intra
Peri1mm
Peri2mm
Peri3mm
Peri4mm
Peri5mm
Ensemble
MIL
Combined
0.684
0.436 0.730
0.217 0.361 0.514
0.740 0.938 0.669 0.202
0.642 0.975 0.697 0.367 0.968
0.565 0.915 0.789 0.454 0.859 0.863
0.166 0.242 0.353 0.658 0.193 0.238 0.252
0.060 0.101 0.136 0.351 0.087 0.074 0.083 0.285
0.005 0.003 0.006 0.022 0.002 0.004 0.002 0.057 0.084
Clinical
Intra
Peri1mm
Peri2mm
Peri3mm
Peri4mm
Peri5mm
Ensemble
MIL
0.8
0.6
0.4
0.2

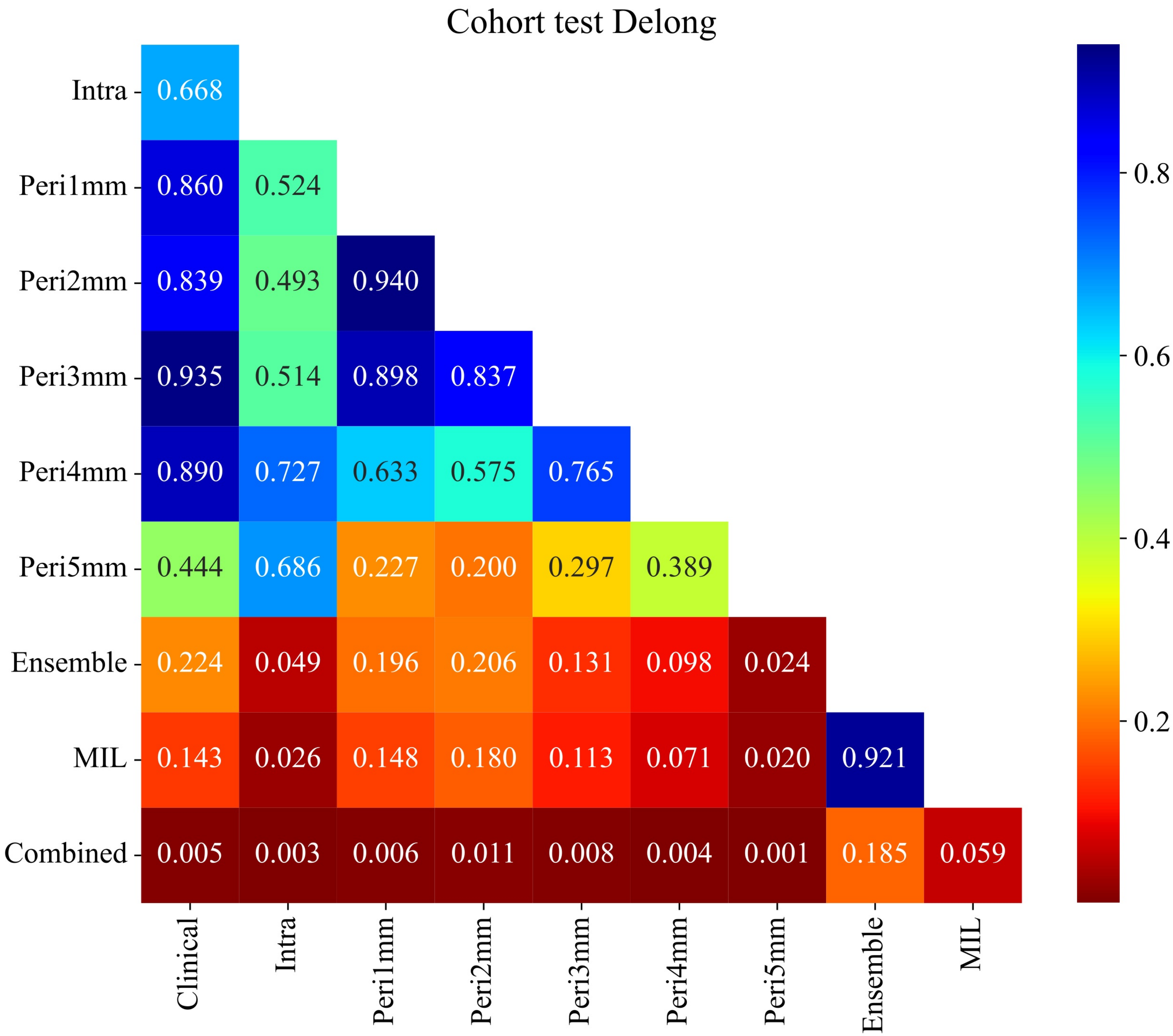
Cohort test Delong
Intra 0.668
Peri1mm 0.860 0.524
Peri2mm 0.839 0.493 0.940
Peri3mm 0.935 0.514 0.898 0.837
Peri4mm 0.890 0.727 0.633 0.575 0.765
Peri5mm 0.444 0.686 0.227 0.200 0.297 0.389
Ensemble 0.224 0.049 0.196 0.206 0.131 0.098 0.024
MIL 0.143 0.026 0.148 0.180 0.113 0.071 0.020 0.921
Combined 0.005 0.003 0.006 0.011 0.008 0.004 0.001 0.185 0.059
Clinical
Intra
Peri1mm
Peri2mm
Peri3mm
Peri4mm
Peri5mm
Ensemble
MIL
0.8
0.6
0.4
0.2

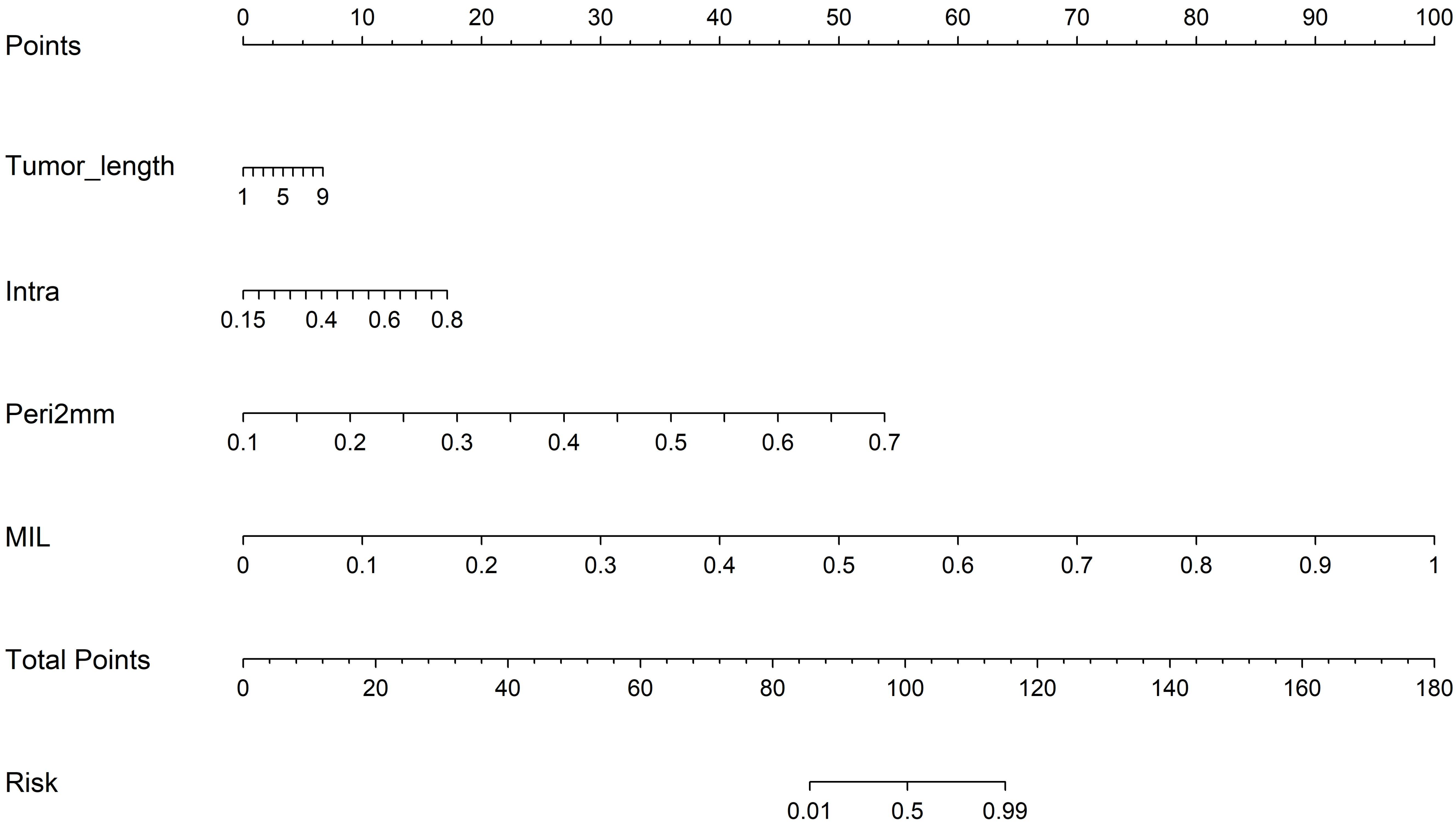

Points
0 10 20 30 40 50 60 70 80 90 100
Tumor_length
1 5 9
Intra
0.15 0.4 0.6 0.8
Peri2mm
0.1 0.2 0.3 0.4 0.5 0.6 0.7
MIL
0 0.1 0.2 0.3 0.4 0.5 0.6 0.7 0.8 0.9 1
Total Points
0 20 40 60 80 100 120 140 160 180
Risk
0.01 0.5 0.99
